# YTHDC1 Is Essential for Postnatal Liver Development and Homeostasis

**DOI:** 10.1002/advs.202505725

**Published:** 2025-06-19

**Authors:** Xinzhi Li, Xueying Li, Chunhong Liu, Zhenzhi Li, Kaixin Ding, Yuxin Wang, Ning Gu, Liwei Xie, Zheng Chen

**Affiliations:** ^1^ HIT Center for Life Sciences School of Life Science and Technology State Key Laboratory of Matter Behaviors in Space Environment Frontier Science Center for Interaction between Space Environment and Matter Zhengzhou Research Institute Harbin Institute of Technology Harbin 150001 China; ^2^ NHC Key Laboratory of Cell Transplantation The First Affiliated Hospital of Harbin Medical University Harbin 150001 China; ^3^ Guangdong Provincial Key Laboratory of Microbial Culture Collection and Application State Key Laboratory of Applied Microbiology Southern China Institute of Microbiology Guangdong Academy of Sciences Guangzhou 510070 China

**Keywords:** FOXA1, FOXA2, liver, liver injury, m^6^A modification, postnatal development, YTHDC1

## Abstract

Hepatocytes play a crucial role in liver function, with their maturation occurring during postnatal development. However, the molecular mechanisms underlying this maturation are not yet fully understood. In this study, YTHDC1 is identified as a key positive regulator of hepatocyte maturation and an essential factor in maintaining liver homeostasis. YTHDC1 expression increases in the liver after birth, and hepatocyte‐specific deletion of *Ythdc1* impairs hepatocyte maturation, leading to reduced liver weight, liver injury, inflammation, and fibrosis. These defects contribute to the development of nonalcoholic steatohepatitis and hepatocellular carcinoma in mice. YTHDC1 supports postnatal hepatocyte maturation and liver function by enhancing the expression of key transcription factors, FOXA1 and FOXA2, at the posttranscriptional level through m^6^A recognition. Restoring FOXA1 or FOXA2 expression mitigates the phenotypic defects observed in *Ythdc1*‐HKO mice. Mechanistically, YTHDC1 binds to the m^6^A regions of *Foxa1* and *Foxa2* mRNAs, promoting their expression. These findings reveal a mechanism by which YTHDC1 regulates hepatocyte maturation.

## Graphical abstract

This study identifies YTHDC1 as a key regulator of postnatal liver development and disease. Hepatocyte‐specific deletion of *Ythdc1* impairs hepatocyte maturation, causing liver injury, contributing to nonalcoholic steatohepatitis and hepatocellular carcinoma. YTHDC1 promotes hepatocyte maturation by enhancing FOXA1 and FOXA2 expression via m^6^A modification recognition, offering new insights into its role in liver development and disease pathogenesis.

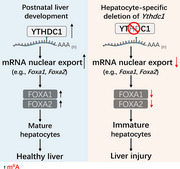

## 1 Introduction

The liver, the largest organ in the body, performs over 500 critical functions, including the production of albumin, bile acids, and essential nutrients; regulation of blood glucose, amino acid, and lipid levels; and detoxification of harmful substances from the bloodstream.^[^ ^1^, ^2^ ^]^ Hepatocytes are central to these functions, and their maturation during postnatal development is essential. Impaired hepatocyte maturation can lead to liver injury and inflammation, which, in turn, promote liver diseases such as nonalcoholic fatty liver disease (NAFLD), nonalcoholic steatohepatitis (NASH), and hepatocellular carcinoma (HCC).^[^ ^2^ ^]^ Over the past 30 years, several transcription factors—such as the FoxA family (HNF3, FOXA1/2/3), the Cuthomeodomain family (HNF6), the bZIP family (CEBPα), the Pouhomeodomain family (HNF1α), orphan receptors (HNF4α), the GATA family (GATA4/5/6), and histone modification‐related proteins like EZH1/2—have been identified as key regulators of liver development.^[^ ^1^, ^2^ ^]^ Downregulation or deletion of any of these factors disrupts liver development and causes liver diseases.^[^ ^3^, ^4^ ^]^ Despite these insights, the molecular mechanisms driving hepatocyte maturation during postnatal development remain unclear, highlighting the need to identify key factors that regulate this process.

In addition to transcription factors that govern gene transcription, RNA‐binding proteins (RBPs)—a group of over 1500 proteins—play a critical role in RNA processing, including mRNA capping, polyadenylation, modification, splicing, stabilization, localization, and translation.^[^ ^5^ ^]^ Emerging evidence suggests that certain RBPs are essential for liver function. For instance, the RBPs NONO, Lin28a/b, and A1CF are crucial for regulating hepatic glucose homeostasis.^[^ ^6^ ^]^ Additionally, N6‐methyladenosine (m^6^A) writer proteins, such as METTL3 and WTAP, have been shown to maintain liver homeostasis through both m^6^A‐dependent and ‐independent mechanisms.^[^ ^7^, ^8^ ^]^ Therefore, identifying RBPs that regulate hepatocyte maturation is vital.

Through RNA‐seq analysis of liver samples from mice at various postnatal ages, we identified 856 RBPs with differential expression, including YTH domain‐containing 1 (YTHDC1). Initially named YT521‐B, YTHDC1 was found to localize to nuclear speckles.^[^ ^9^ ^]^ Subsequent research demonstrated that YTHDC1 binds to single‐stranded RNA sequence motifs and recognizes m^6^A modifications as one of the m^6^A reader proteins, thereby regulating RNA splicing, nuclear RNA export, and RNA decay.^[^ ^10^, ^11^, ^12^, ^13^, ^14^ ^]^ More recently, YTHDC1 has also been shown to bind to DNA and regulate chromatin accessibility.^[^ ^14^, ^15^ ^]^ Deletion of *Ythdc1* is embryonically lethal, underscoring its essential role in embryonic development in mice.^[^ ^16^ ^]^ We have recently shown that brown adipocyte‐specific deletion of *Ythdc1* impairs postnatal development of brown adipose tissue,^[^ ^17^ ^]^ and islet β cell specific deletion *Ythdc1* leads to β cell failure and diabetes.^[^ ^18^ ^]^ However, the role of YTHDC1 in liver development and disease has remained largely unexplored.

In this study, we identify YTHDC1 as a critical regulator of liver development and disease. YTHDC1 expression increases postnatally, and hepatocyte‐specific deletion of *Ythdc1* results in impaired liver development, liver injury, inflammation, and fibrosis, contributing to the pathogenesis of NASH and HCC. YTHDC1 promotes postnatal hepatocyte maturation by enhancing the expression of liver transcription factors FOXA1 and FOXA2, partially through its recognition of m^6^A modifications. Restoring FOXA1 and FOXA2 expression alleviates the phenotypes observed in *Ythdc1*‐HKO mice. Mechanistically, YTHDC1 binds to the m^6^A‐modified regions of *Foxa1* and *Foxa2* mRNAs, enhancing their expression and facilitating their nuclear‐to‐cytosolic translocation. These findings provide new insights into the mechanism by which YTHDC1 regulates hepatocyte maturation.

## 2 Results

### 2.1 Global Gene Expression Profiles in the Liver during Postnatal Development Reveal YTHDC1 as a Potential Regulator of Liver Development

**Figure 1:**
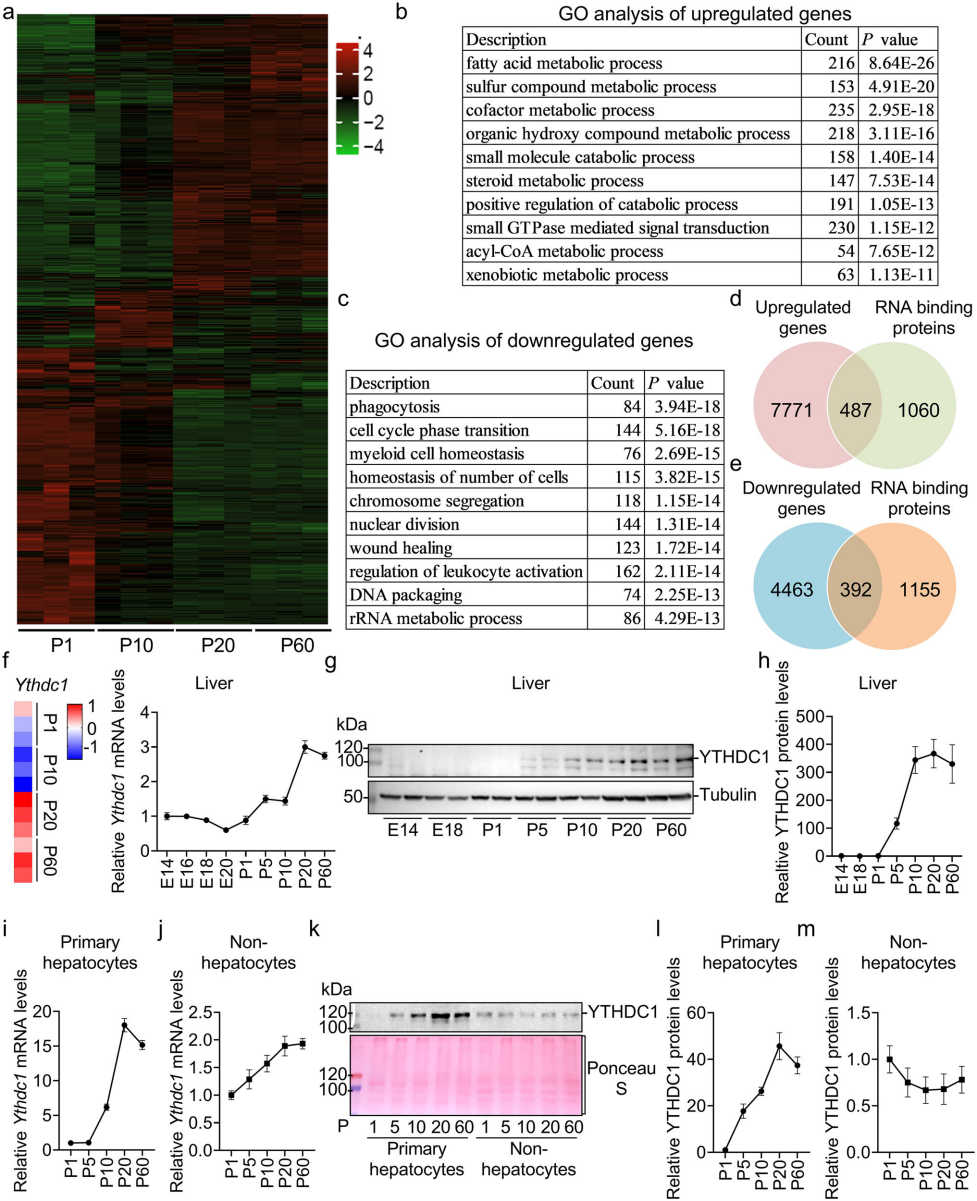
Global gene expression profiles in the liver during postnatal development reveal YTHDC1 as a potential regulator of liver development. a) RNA‐seq analysis was performed on mouse livers at postnatal days 1, 10, 20, and 60. Differentially expressed genes (DEGs) were identified between P10 and P1, P20 and P10, and P60 and P20. These DEGs were combined and visualized using a heatmap. b) Differentially upregulated genes were identified by RNA‐seq analysis between P10 and P1, P20 and P10, and P60 and P20. These upregulated genes were then combined, and Gene ontology (GO) analysis was performed. c) Differentially downregulated genes were identified by RNA‐seq analysis between P10 and P1, P20 and P10, and P60 and P20. These downregulated genes were then combined, and GO analysis was performed. d) A Venn diagram revealed that 487 RNA‐binding proteins (RBPs) were upregulated in the liver during postnatal development. The upregulated genes were identified in panels (a) and (b). e) A Venn diagram showed that 392 RNA‐binding proteins (RBPs) were downregulated in the liver during postnatal development. The downregulated genes were identified in panels (a) and (c). f) A heatmap showed that *Ythdc1* was significantly upregulated in the liver during postnatal development. *Ythdc1* mRNA levels at embryonic day 14, 16, 18, 20, postnatal day 1, 5, 10, 20, and 60 were measured by RT‐qPCR (*n* = 7 per group). g,h) YTHDC1 protein levels at embryonic day 14, 18, postnatal day 1, 5, 10, 20, and 60 were measured by immunoblotting and quantified using ImageJ (*n* = 2 for representative, *n* = 8 for quantification). i–m) Hepatocytes and nonhepatocytes were isolated from mice at 1, 5, 10, 20, and 60 days old, respectively. i,j) *Ythdc1* mRNA levels in hepatocytes and nonhepatocytes were measured by RT‐qPCR (*n* = 4 per group). k–m) YTHDC1 protein levels in hepatocytes and nonhepatocytes were measured by immunoblotting and quantified using ImageJ (*n* = 2 for representative, *n* = 4 for quantification). *n* was the number of biologically independent mice. Data represent the mean ± SEM.

To comprehensively analyze gene expression changes in the liver during postnatal development, we performed RNA‐seq on liver samples collected at different postnatal stages (P1, P10, P20, and P60). As shown in **Figure** **1**a; Figure S1a–d and Table S1 (Supporting Information), we identified 8258 genes (subclusters 1 and 4) that were gradually upregulated and 4855 genes (subclusters 2 and 3) that were downregulated during postnatal development. Gene ontology (GO) analysis revealed that the upregulated genes were associated with processes such as fatty acid metabolism, sulfur compound metabolism, cofactor metabolism, organic hydroxy compound metabolism, small molecule catabolism, steroid metabolism, positive regulation of catabolism, small GTPase‐mediated signal transduction, acyl‐CoA metabolism, and xenobiotic metabolism (Figure 1b). In contrast, the downregulated genes were linked to processes including phagocytosis, cell cycle phase transition, myeloid cell homeostasis, cellular homeostasis, chromosome segregation, nuclear division, wound healing, regulation of leukocyte activation, DNA packaging, and rRNA metabolism (Figure 1c).

Upon further analysis, we observed that, compared to postnatal day 1 (P1), 2575 genes were upregulated on postnatal day 10 (P10), primarily associated with metabolic processes such as fatty acid metabolism, organic acid catabolism, cellular lipid catabolism, and steroid metabolism (Figure S2a,d, Supporting Information). Conversely, 2649 genes were downregulated, related to processes including rRNA processing, ribosome biogenesis, chromosome segregation, ribonucleoprotein complex biogenesis, erythrocyte homeostasis, and cytoplasmic translation (Figure S2a,d, Supporting Information). When comparing P20 to P10, 3288 genes were upregulated, linked to xenobiotic metabolism, protein polyubiquitination, acyl‐CoA metabolism, thioester metabolism, lipid biosynthesis, macroautophagy, and positive regulation of glucose import. Additionally, 3072 genes were downregulated, associated with processes such as negative regulation of peptidase activity, leukocyte differentiation, phagocytosis, positive regulation of cytokine production, cell activation, T cell activation, and leukocyte migration (Figure S2b,e, Supporting Information). Finally, comparing P60 to P20, we identified 1410 upregulated genes associated with cellular hormone metabolism, retinoid metabolism, xenobiotic metabolism, and synaptic vesicle transport. In contrast, 894 downregulated genes were linked to chromosome segregation, nuclear division, DNA‐dependent DNA replication, and cell cycle phase transition (Figure S2c,f, Supporting Information). These results suggest that at each postnatal time point, specific liver functions are achieved by inducing the expression of genes related to processes such as fatty acid metabolism, organic acid catabolism, cellular lipid catabolism, steroid metabolism, xenobiotic metabolism, macroautophagy, positive regulation of glucose import, and cellular hormone metabolism. In contrast, fetal liver functions, such as hemopoiesis, are suppressed during postnatal development as a result of the downregulation of specific gene expression. Thus, these findings imply that postnatal liver development is intricately regulated.

Given that both transcription factors (TFs) and RBPs control the expression of many genes, they are likely involved in this complex regulation of postnatal liver development. We then analyzed the expression of TFs and RBPs during this period. As shown in Figure 1d,e and Table S2 (Supporting Information), 554 TFs and 487 RBPs were upregulated, while 208 TFs and 392 RBPs were downregulated throughout postnatal liver development. Upregulated RBPs and TFs may cooperatively promote postnatal liver development. The m^6^A modification‐related proteins (METTL3, METTL14, and WTAP) are known to play key roles in liver development and liver diseases.^[^ ^7^, ^8^, ^19^, ^20^, ^21^ ^]^ Notably, the m^6^A reader proteins YTHDC1 and YTHDF2/3 were among the upregulated RBPs (Figure 1d,f; Figure S3a, Supporting Information). RT‐qPCR validation confirmed these findings (Figure 1f; Figure S3b, Supporting Information), while immunoblotting showed that YTHDC1 and YTHDF2/3 protein levels were undetectable at embryonic stage E16 but increased by postnatal day 10, reaching their peak levels by day 20 after birth (Figure 1g,h; Figure S3c,d, Supporting Information). These results suggest that these proteins may play a crucial role in regulating postnatal liver development.

Among the three m^6^A reader proteins, YTHDC1 exhibits the most significant upregulation (more than 300‐fold vs 10–30‐fold) (Figure 1g,h; Figure S3c,d, Supporting Information) and is the only one localized to the nucleus,^[^ ^9^ ^]^ where it likely plays a critical role in postnatal liver development by facilitating RNA transport from the nucleus to the cytosol.^[^ ^13^ ^]^ As a result, we focused on investigating the role of YTHDC1 in this process. YTHDC1 is expressed in the liver as well as other tissues (Figure S4a, Supporting Information). It is highly expressed in hepatocytes compared to nonhepatocytes (Figure S4b, Supporting Information). The upregulation of YTHDC1 during postnatal liver development primarily occurs in hepatocytes, rather than in nonhepatocytes (Figure 1i–m). These findings further suggest that YTHDC1 plays a crucial role in hepatocyte maturation and postnatal liver development.

### 2.2 Hepatocyte‐Specific Deletion of *Ythdc1* Impairs Liver Development and Leads to Liver Injury, Inflammation, and Fibrosis

**Figure 2:**
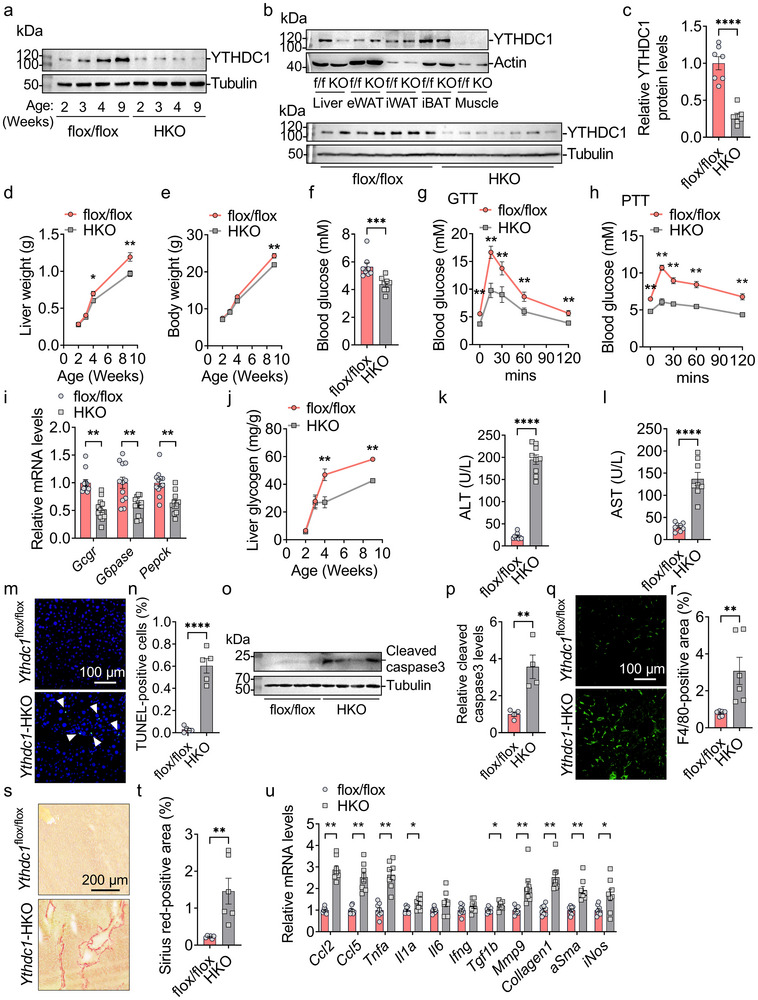
Hepatocyte‐specific deletion of *Ythdc1* impairs liver development and causes liver injury, inflammation, and fibrosis. a) YTHDC1 and Tubulin protein levels were measured by immunoblotting in the livers of male *Ythdc1* ^flox/flox^ and *Ythdc1‐*HKO mice at 2, 3, 4, and 9 weeks old. b) Top: YTHDC1 and Actin protein levels were measured by immunoblotting in the liver, iBAT, eWAT, iWAT, and skeletal muscle of male *Ythdc1* ^flox/flox^ and *Ythdc1‐*HKO mice at 9 weeks old. Bottom: YTHDC1 and Tubulin protein levels in the livers of these mice were measured by immunoblotting and quantified using ImageJ. c) Immunoblotting data from (b) bottom were quantified (*n* = 7 per group). d,e) Liver and body weights were measured in male *Ythdc1* ^flox/flox^ and *Ythdc1‐*HKO mice at 2, 3, 4, and 9 weeks old (*n* = 7–16 per group). f) Fasting blood glucose was measured in male *Ythdc1* ^flox/flox^ and *Ythdc1‐*HKO mice at 8 weeks old (*n* = 8 per group). g,h) Glucose tolerance tests (GTT) and pyruvate tolerance tests (PTT) were conducted in male *Ythdc1* ^flox/flox^ and *Ythdc1‐*HKO mice at 8 weeks old (*n* = 8 per group). i) Relative mRNA levels were measured by RT‐qPCR (*n* = 12 per group). j) Liver glycogen levels were measured in male *Ythdc1* ^flox/flox^ and *Ythdc1‐*HKO mice at 2, 3, 4, and 9 weeks old (*n* = 9 per group). k,l) Serum ALT and AST activities were determined in male *Ythdc1* ^flox/flox^ and *Ythdc1‐*HKO mice at 9 weeks old (*n* = 8–9 per group). m,n) TUNEL staining was performed on liver sections from male *Ythdc1* ^flox/flox^ and *Ythdc1‐*HKO mice at 9 weeks old (*n* = 5 per group). o,p) Cleaved caspase 3 protein levels in male *Ythdc1* ^flox/flox^ and *Ythdc1‐*HKO livers were measured by immunoblotting and quantified using ImageJ (*n* = 4 per group). q,r) F4/80 immunostaining was conducted on liver sections from male *Ythdc1* ^flox/flox^ and *Ythdc1‐*HKO mice at 9 weeks old (*n* = 6 per group). s,t) Sirius Red staining was performed on liver sections from male *Ythdc1* ^flox/flox^ and *Ythdc1‐*HKO mice at 9 weeks old (*n* = 6 per group). u) RT‐qPCR analysis of mRNA levels was conducted in the livers of male *Ythdc1* ^flox/flox^ and *Ythdc1‐*HKO mice at 9 weeks old (*n* = 8 per group). *n* was the number of biologically independent mice. Data represent the mean ± SEM. The Shapiro–Wilk test was employed to assess the normality of the data. When both groups were normally distributed (*P* > 0.05), the parametric two‐tailed Student's *t*‐tests were used to detect the statistical differences between the two groups. When at least one of the two groups were not normally distributed (*P* < 0.05), the nonparametric Mann–Whitney test was adopted to compare the statistical differences between the two groups. *, *P* < 0.05. **, *P* < 0.01. ***, *P* < 0.001. ****, *P* < 0.0001.

To investigate the role of YTHDC1 in postnatal liver development, we generated hepatocyte‐specific *Ythdc1* knockout mice by crossing *Ythdc1* ^flox/flox^ mice with hepatocyte‐specific *Albumin* promoter‐driven Cre recombinase (*Alb*‐Cre) transgenic mice. The genotype of *Ythdc1*‐HKO mice was *Ythdc1* ^flox/flox^ *Alb*‐Cre^+/‐^. *Alb* mRNA is first detected at embryonic Day 10.5 (E10.5) in the hepatic primordial,^[^ ^22^ ^]^ and *Alb*‐Cre transgene efficiently deletes *Ythdc1* floxed alleles by 3 weeks of age (**Figure** **2**a). YTHDC1 was specifically deleted in liver, but not in other tissues such as epididymal white adipose tissue (eWAT), inguinal white adipose tissue (iWAT), interscapular brown adipose tissue (iBAT), and skeletal muscle, in 8‐week‐old *Ythdc1*‐HKO mice (Figure 2b,c). No differences in body weight (Figure S5a, Supporting Information) or liver weight (Figure S5b, Supporting Information) were observed between *Ythdc1* ^flox/flox^ and *Alb*‐Cre mice. Therefore, *Ythdc1* ^flox/flox^ mice were used as controls for the *Ythdc1*‐HKO mice in subsequent experiments. Interestingly, the liver weight of *Ythdc1*‐HKO mice was significantly lower than that of *Ythdc1* ^flox/flox^ mice starting at 4 weeks of age (Figure 2d), which was accompanied by reduced body weight gain in *Ythdc1*‐HKO mice during postnatal development (Figure 2e). These findings suggest that YTHDC1 is essential for postnatal liver development.

Maintaining glucose homeostasis is one of the liver's key functions. To determine whether hepatic YTHDC1 is necessary for glucose homeostasis, we measured fasting blood glucose in *Ythdc1* ^flox/flox^ and *Ythdc1*‐HKO mice. As shown in Figure 2f, fasting blood glucose was lower in *Ythdc1*‐HKO mice compared to *Ythdc1* ^flox/flox^ mice. Hepatic gluconeogenesis was impaired, as evidenced by decreased glucose levels following glucose or pyruvate injections (Figure 2g,h) and by reduced expression of gluconeogenesis‐related genes (*Gcgr*, *G6pase*, and *Pepck*) (Figure 2i). Additionally, liver glycogen levels were lower in *Ythdc1*‐HKO mice starting at 4 weeks of age (Figure 2j). These results suggest that hepatocyte‐specific deletion of *Ythdc1* disrupts glucose homeostasis in the liver.

The liver also plays a crucial role in lipid homeostasis. To determine whether hepatic YTHDC1 is essential for lipid homeostasis, we measured serum free fatty acids (FFAs), triacylglycerol (TAG), and liver TAG levels in *Ythdc1* ^flox/flox^ and *Ythdc1*‐HKO mice. As shown in Figure S5c–e (Supporting Information), no significant differences were observed in serum FFAs, serum TAG, or liver TAG levels between *Ythdc1* ^flox/flox^ and *Ythdc1*‐HKO mice, suggesting that hepatic YTHDC1 is not required for maintaining lipid homeostasis.

To assess whether hepatocyte‐specific deficiency of *Ythdc1* leads to liver injury and inflammation, we measured serum alanine aminotransferase (ALT) and aspartate aminotransferase (AST) activities, TUNEL‐positive cells, cleaved caspase 3, and liver injury‐related markers in *Ythdc1* ^flox/flox^ and *Ythdc1*‐HKO mice. Serum ALT and AST activities were significantly elevated in *Ythdc1*‐HKO mice (Figure 2k,l), suggesting that hepatocyte‐specific deletion of *Ythdc1* causes liver injury and inflammation. TUNEL assays revealed a significant increase in TUNEL‐positive cells in the livers of *Ythdc1*‐HKO mice (Figure 2m,n), indicating enhanced hepatocyte apoptosis. Furthermore, cleaved caspase 3 levels were significantly elevated in *Ythdc1*‐HKO livers (Figure 2o,p). This cell death also triggered immune cell infiltration, as evidenced by a marked increase in F4/80‐positive cells (Figure 2q,r). Additionally, liver sections from *Ythdc1*‐HKO mice displayed significantly larger Sirius Red‐positive areas compared to *Ythdc1* ^flox/flox^ mice (Figure 2s,t). Consistent with these observations, the expression of fibrosis markers (*Collagen 1*, *aSma*, and *Mmp9*), profibrogenic factor *Tgfb1*, and proinflammatory cytokines (*Ccl2*, *Ccl5*, *Tnfa*, *Il1a*, *iNos*) were significantly upregulated in the livers of *Ythdc1*‐HKO mice (Figure 2u). These findings demonstrate that hepatocyte‐specific deletion of *Ythdc1* results in liver injury, inflammation, and fibrosis.

To determine whether liver injury and inflammation are the primary causes of impaired liver function in *Ythdc1*‐HKO mice, we assessed serum ALT activity and the number of TUNEL‐positive cells in 3‐ and 4‐week‐old *Ythdc1*‐HKO and *Ythdc1* ^flox/flox^ mice. As shown in Figure S6a,b (Supporting Information), both *Ythdc1*‐HKO and *Ythdc1* ^flox/flox^ mice exhibited normal serum ALT activity and similar numbers of TUNEL‐positive cells. These findings suggest that liver injury and inflammation are not the primary contributors to the impaired liver function observed in *Ythdc1*‐HKO mice.

### 2.3 Adult‐Onset Hepatocyte‐Specific Deletion of *Ythdc1* Disrupts Glucose Homeostasis and Induces Liver Injury

**Figure 3:**
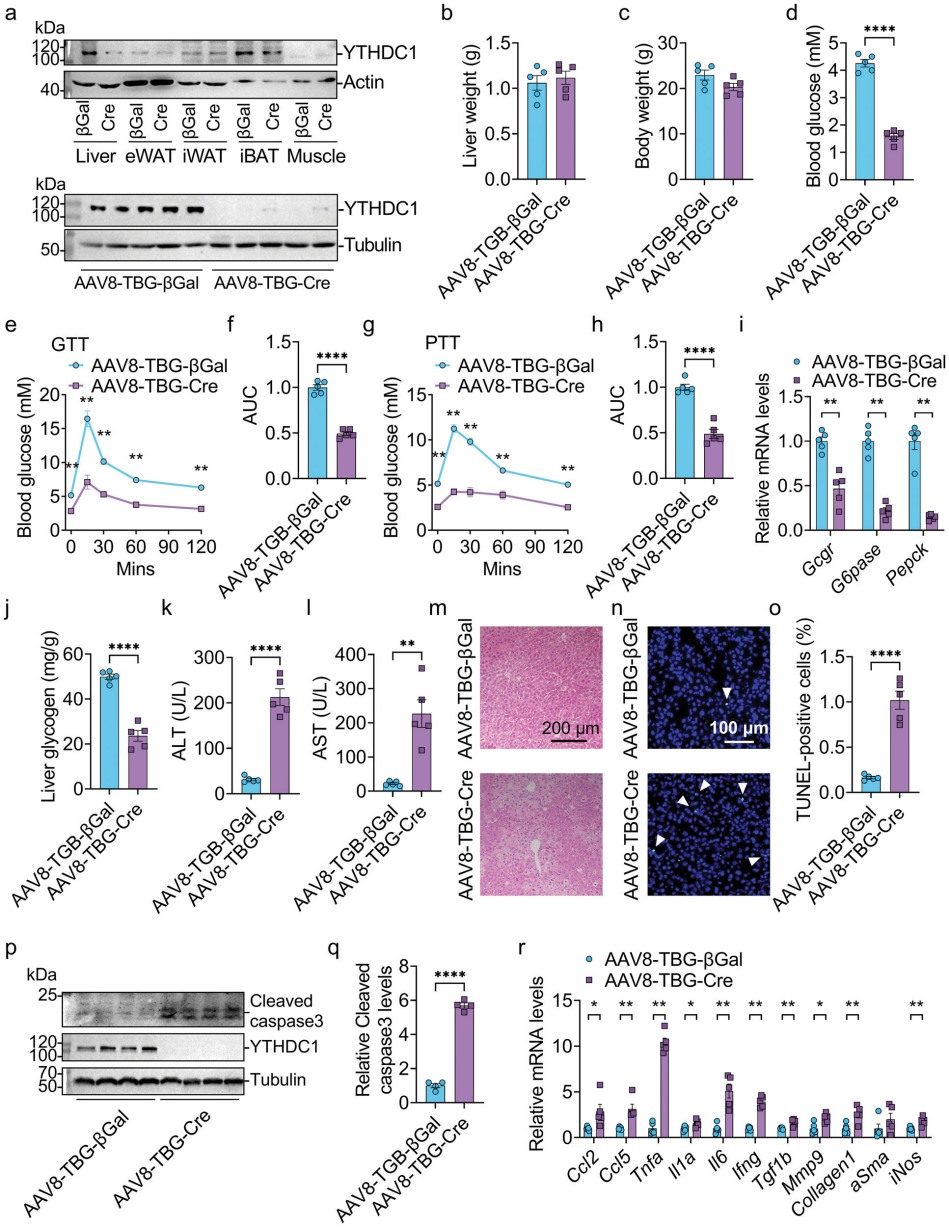
Adult‐onset hepatocyte‐specific deletion of *Ythdc1* impairs glucose homeostasis and causes liver injury. *Ythdc1* ^flox/flox^ mice at 8 weeks old were injected with AAV8–TBG–Cre or AAV8–TBG–βGal via tail vein for 16 days. a) Top: YTHDC1 and actin protein levels were measured in the liver, eWAT, iWAT, iBAT, and skeletal muscle of *Ythdc1* ^flox/flox^‐βGal and *Ythdc1‐*adultHKO mice at 16 days post‐AAV injection by immunoblotting. Bottom: YTHDC1 and Tubulin protein levels in the livers of these mice were also measured. b) Liver weight was measured (*n* = 5 per group). c) Body weight was measured (*n* = 5 per group). d) Fasting blood glucose levels were measured (*n* = 5 per group). e–h) Glucose tolerance tests (GTT) and relative AUC, as well as pyruvate tolerance tests (PTT) and relative AUC, were measured (*n* = 5 per group). i) Relative *Gcgr*, *G6pase*, and *Pepck* mRNA levels were measured by RT‐qPCR (*n* = 5 per group). j) Liver glycogen levels were measured (*n* = 5 per group). k,l) Serum ALT and AST activities were determined (*n* = 5 per group). m) H&E staining was performed. Representative images were shown. n,o) TUNEL‐positive cells in liver sections were measured (*n* = 5 per group). p,q) Cleaved caspase 3 protein levels were measured by immunoblotting and quantified using ImageJ (*n* = 4 per group). r) RT‐qPCR analysis of mRNA levels in the livers (*n* = 5 per group). *n* was the number of biologically independent mice. Data represent the mean ± SEM. The Shapiro–Wilk test was employed to assess the normality of the data. When both groups were normally distributed (*P* > 0.05), the parametric two‐tailed Student's *t*‐tests were used to detect the statistical differences between the two groups. When at least one of the two groups were not normally distributed (*P* < 0.05), the nonparametric Mann–Whitney test was adopted to compare the statistical differences between the two groups. *, *P* < 0.05.**, *P* < 0.01. ****, *P* < 0.0001.

To investigate whether YTHDC1 is essential for maintaining liver function in adult mice, we generated adult‐onset hepatocyte‐specific *Ythdc1* knockout (*Ythdc1*‐adultHKO) mice by tail‐vein injection of purified AAV8–TBG–Cre virus into adult *Ythdc1* ^flox/flox^ mice. Adult *Ythdc1* ^flox/flox^ mice injected with an equal amount of AAV8–TBG–βGal virus served as controls. As expected, YTHDC1 was specifically deleted in the liver, with no deletion observed in other tissues such as eWAT, iWAT, iBAT, and skeletal muscle (**Figure** **3**a). Although adult‐onset hepatic deficiency of *Ythdc1* did not affect liver or body weights (Figure 3b,c), it significantly impaired glucose homeostasis (Figure 3d–j). *Ythdc1*‐adultHKO mice exhibited markedly lower blood glucose levels (Figure 3d). Hepatic gluconeogenesis was impaired, as evidenced by decreased glucose levels following injection of glucose or pyruvate (Figure 3e–h) and by reduced expression of gluconeogenesis‐related genes (*Gcgr*, *G6pase*, and *Pepck*) (Figure 3i). Additionally, *Ythdc1*‐adultHKO mice displayed reduced liver glycogen levels (Figure 3j). These findings suggest that adult‐onset hepatocyte‐specific deletion of *Ythdc1* impairs hepatic glucose homeostasis.

To determine whether adult‐onset hepatocyte‐specific deletion of *Ythdc1* induces liver injury and inflammation, we assessed serum ALT and AST activities, TUNEL‐positive cells, cleaved caspase 3, and liver inflammation‐related markers in *Ythdc1* ^flox/flox^‐βGal and *Ythdc1*‐adultHKO mice. Serum ALT and AST activities were significantly elevated in *Ythdc1*‐adultHKO mice (Figure 3k,l), indicating that adult‐onset hepatocyte‐specific deletion of *Ythdc1* causes liver injury and inflammation. H&E staining revealed severe liver injury in *Ythdc1*‐adultHKO mice (Figure 3m). TUNEL assays showed a significant increase in TUNEL‐positive cells in the livers of *Ythdc1*‐adultHKO mice (Figure 3n,o). Additionally, cleaved caspase 3 levels were significantly elevated in *Ythdc1*‐adultHKO livers (Figure 3p,q). Consistent with these findings, the expression of fibrosis markers (*Collagen 1* and *Mmp9*), the profibrogenic factor *Tgfb1*, and proinflammatory cytokines (*Ccl2*, *Ccl5*, *Tnfa*, *Il1a*, *Il6*, *Ifng*, and *iNos*) were significantly upregulated in the livers of *Ythdc1*‐adultHKO mice (Figure 3r). These data demonstrate that adult‐onset hepatocyte‐specific deletion of *Ythdc1* leads to liver injury and inflammation.

### 2.4 Hepatocyte‐Specific Deletion of *Ythdc1* Accelerates the Pathogenesis of NASH and HCC

**Figure 4:**
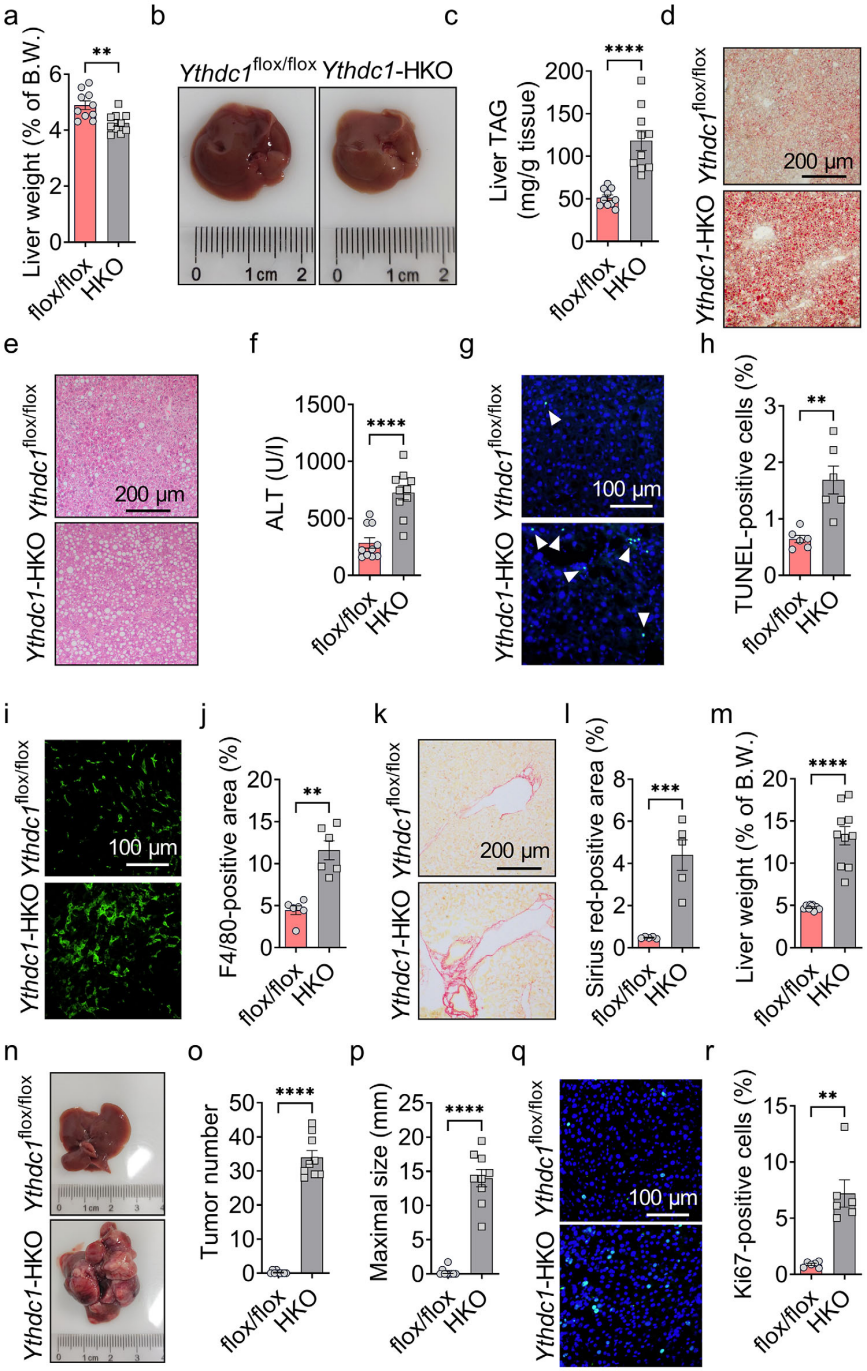
Hepatocyte‐specific deletion of *Ythdc1* accelerates pathogenesis of NASH and HCC. a) Liver weights of *Ythdc1* ^flox/flox^ and *Ythdc1‐*HKO mice fed an MCD diet for 10 days were measured (*n* = 10 per group). b) Representative pictures of livers from *Ythdc1* ^flox/flox^ and *Ythdc1‐*HKO mice fed an MCD diet for 10 days were shown. c) Liver TAG levels were measured in *Ythdc1* ^flox/flox^ and *Ythdc1‐*HKO mice fed an MCD diet for 10 days (*n* = 10 per group). d) Oil red O staining was performed on liver sections from *Ythdc1* ^flox/flox^ and *Ythdc1‐*HKO mice fed an MCD for 10 days. e) H&E staining was performed on liver sections from *Ythdc1* ^flox/flox^ and *Ythdc1‐*HKO mice fed an MCD for 10 days. Representative images were shown. f) Serum ALT activity was measured in *Ythdc1* ^flox/flox^ and *Ythdc1‐*HKO mice fed an MCD diet for 10 days (*n* = 10 per group). g,h) TUNEL‐positive cells in liver sections from *Ythdc1* ^flox/flox^ and *Ythdc1‐*HKO mice fed an MCD diet for 10 days were quantified (*n* = 6 per group). i,j) F4/80 immunostaining was performed on liver sections from *Ythdc1* ^flox/flox^ and *Ythdc1‐*HKO mice fed an MCD diet for 10 days (*n* = 6 per group). k,l) Sirius Red staining was performed on liver sections from *Ythdc1* ^flox/flox^ and *Ythdc1‐*HKO mice fed an MCD diet for 10 days (*n* = 5 per group). m–r) At two weeks old, male *Ythdc1* ^flox/flox^ and *Ythdc1‐*HKO mice received a single intraperitoneal dose of DEN (50 mg kg^−1^) and were fed an NC diet. Mice were sacrificed at 8 months old for HCC examination. Liver weights were measured (*n* = 9–10 per group) (m). Representative liver images from the indicated genotype were shown (n). Tumor number and maximal tumor size were determined (*n* = 9 per group) (o,p). Representative Ki67 staining images were presented (q). The number of Ki67‐positive cells was counted (*n* = 6 per group) (r). *n* was the number of biologically independent mice. Data represent the mean ± SEM. The Shapiro–Wilk test was employed to assess the normality of the data. When both groups were normally distributed (*P* > 0.05), the parametric two‐tailed Student's *t*‐tests were used to detect the statistical differences between the two groups. When at least one of the two groups were not normally distributed (*P* < 0.05), the nonparametric Mann–Whitney test was adopted to compare the statistical differences between the two groups. *, *P* < 0.05. **, *P* < 0.01. ***, *P* < 0.001. ****, *P* < 0.0001.

Liver injury, inflammation, and fibrosis further promote pathogenesis of NASH and HCC.^[^ ^23^ ^]^ Upon examining published GEO datasets (GSE126848,^[^ ^24^ ^]^ GSE135251,^[^ ^25^ ^]^ GSE119340,^[^ ^26^ ^]^) we observed a reduction in *YTHDC1* mRNA levels in both human samples and mouse models of NASH (Figure S7a–c, Supporting Information), suggesting that YTHDC1 is associated with NASH. To investigate whether hepatocyte‐specific deletion of *Ythdc1* promotes NASH, *Ythdc1*‐HKO, and *Ythdc1* ^flox/flox^ mice were fed a methionine‐ and choline‐deficient diet (MCD) for 10 days. *Ythdc1*‐HKO mice exhibited decreased liver weight and smaller livers (**Figure** **4**a,b). These mice also showed exacerbated MCD‐induced NASH, as indicated by elevated liver TAG levels (Figure 4c), increased hepatic lipid droplets (Figure 4d,e), and higher serum ALT activity (Figure 4f) compared to *Ythdc1* ^flox/flox^ mice. The number of TUNEL‐positive cells was significantly increased in *Ythdc1*‐HKO mice (Figure 4g,h). Hepatocyte death led to immune cell infiltration and liver fibrosis, as evidenced by a marked increase in F4/80‐positive cells (Figure 4i,j). Moreover, liver sections from *Ythdc1*‐HKO mice displayed significantly larger Sirius Red‐positive areas compared to *Ythdc1* ^flox/flox^ mice (Figure 4k,l). These results indicate that *Ythdc1*‐HKO mice are more susceptible to MCD‐induced NASH.

To assess whether the immaturity of hepatocytes in *Ythdc1*‐HKO mice contributes to the pathogenesis of HCC in vivo, we administered a single dose of diethylnitrosamine (DEN) (50 mg kg^−1^) intraperitoneally to *Ythdc1*‐HKO and *Ythdc1* ^flox/flox^ mice at two weeks of age and evaluated liver tumors at 8 months of age. As shown in Figure 4m–p, *Ythdc1*‐HKO mice exhibited increased liver weight and developed a greater number of larger HCCs compared to *Ythdc1* ^flox/flox^ mice. Cell proliferation was significantly elevated in *Ythdc1*‐HKO mice, as indicated by a marked increase in Ki67‐positive cells (Figure 4q,r). These results demonstrate that hepatocyte‐specific deletion of *Ythdc1* accelerates HCC pathogenesis.

### 2.5 Hepatocyte‐Specific Deletion of *Ythdc1* Significantly Alters Gene Expression Profiles

**Figure 5:**
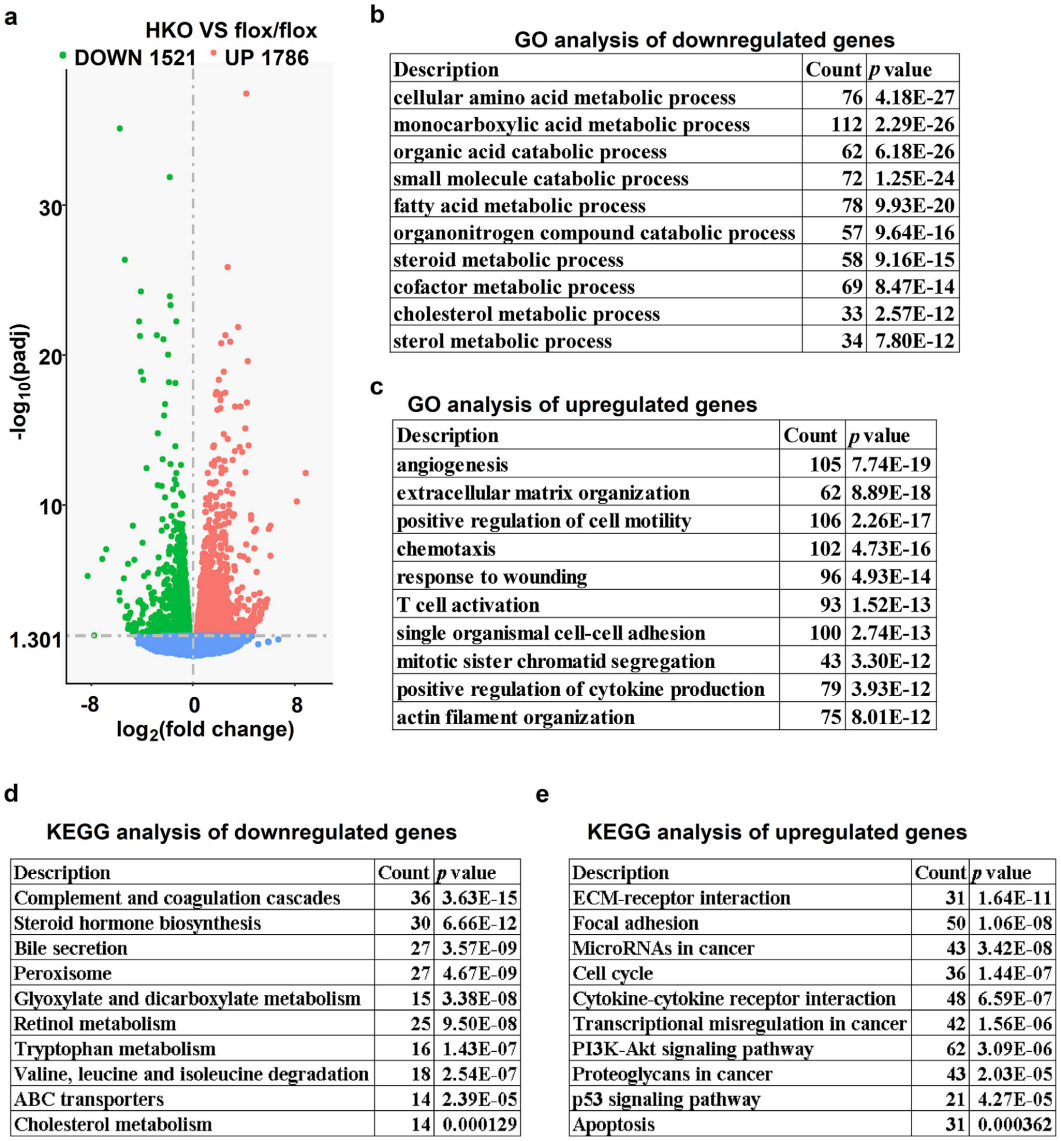
Hepatocyte‐specific deletion of *Ythdc1* significantly alters gene expression profiles. RNA‐seq analysis was performed on the livers of *Ythdc1* ^flox/flox^ and *Ythdc1‐*HKO mice at 9 weeks old (*n* = 3 per group). a) The differentially expressed genes (DEGs) between *Ythdc1‐*HKO and *Ythdc1* ^flox/flox^, including 1521 downregulated genes and 1786 upregulated genes, were illustrated in a volcano plot (|log2foldchange|>0 and pval<0.05). b,c) GO analysis were conducted for downregulated and upregulated genes, respectively. d,e) KEGG analysis was performed for the downregulated and upregulated genes, respectively.

To identify gene transcripts affected in postnatal livers deficient in *Ythdc1*, we performed RNA sequencing (RNA‐seq) analysis on 9‐week‐old *Ythdc1* ^flox/flox^ and *Ythdc1*‐HKO livers. As shown in **Figure** **5**a, 1786 genes were upregulated, while 1521 genes were downregulated. GO analysis revealed that the downregulated genes were associated with processes such as cellular amino acid metabolism, small molecule catabolism, fatty acid metabolism, steroid metabolism, and cholesterol metabolism (Figure 5b). In contrast, genes linked to responses in angiogenesis, extracellular matrix organization, positive regulation of cell motility, chemotaxis, wound healing, T cell activation, mitotic sister chromatid segregation, cytokine production, and actin filament organization were upregulated (Figure 5c). KEGG pathway analysis showed that genes involved in Complement and coagulation cascades, steroid hormone biosynthesis, bile secretion, peroxisome function, glyoxylate and dicarboxylate metabolism, retinol metabolism, ABC transporters, and cholesterol metabolism were downregulated (Figure 5d). Conversely, genes related to ECM–receptor interaction, focal adhesion, the cell cycle, cytokine–cytokine receptor interaction, the PI3K‐Akt signaling pathway, p53 signaling pathway, and apoptosis were significantly upregulated (Figure 5e), suggesting that these processes play an active role in the cellular response to disrupted hepatocyte maturation. These results demonstrate that *Ythdc1* deletion in hepatocytes profoundly alters gene expression profiles, leading to liver dysfunction, inflammation, and injury.

### 2.6 YTHDC1 Is Essential for Maintaining the Expression of Liver‐Enriched Transcription Factors, FOXA1 and FOXA2

**Figure 6:**
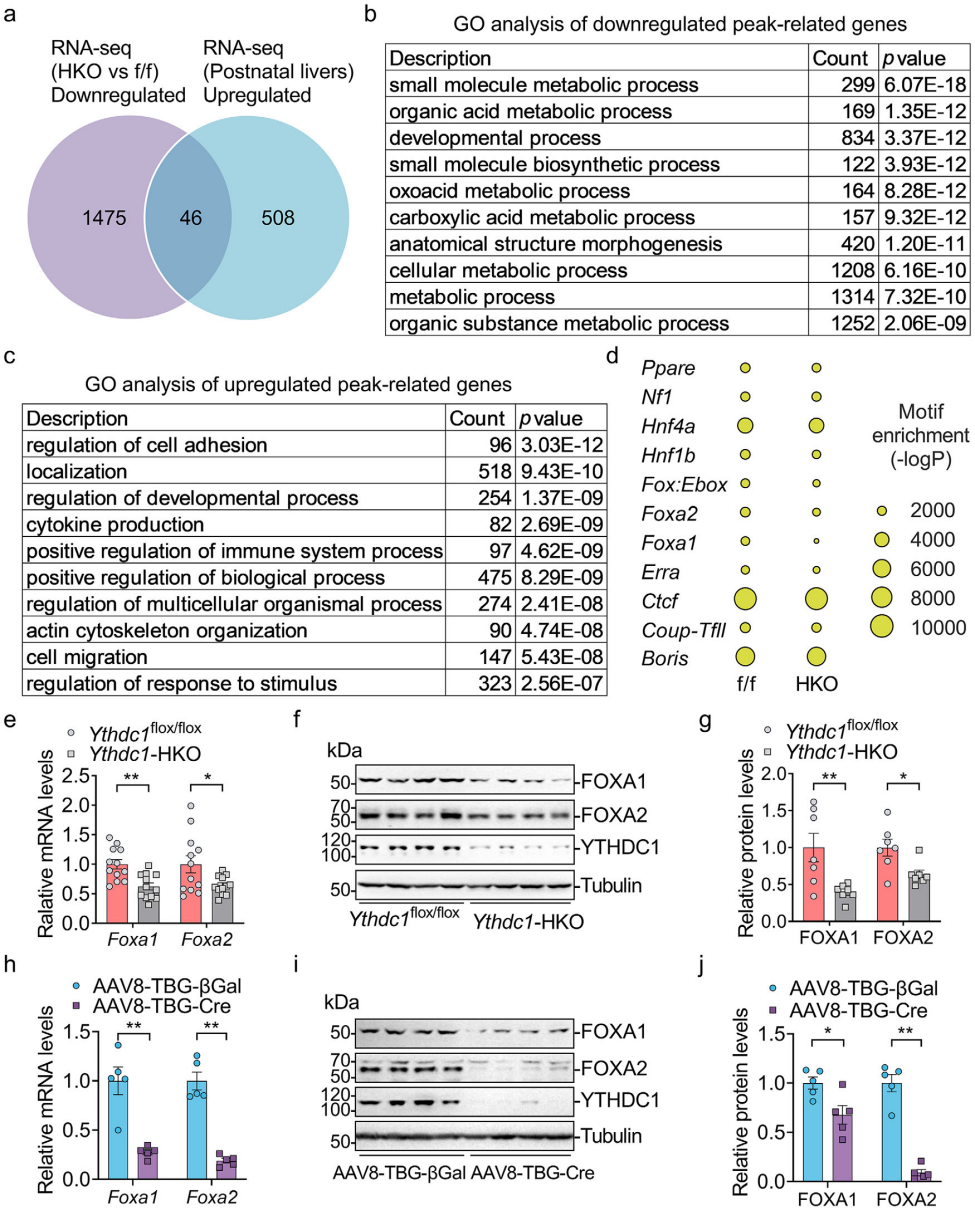
YTHDC1 is essential for maintaining the expression of liver‐enriched transcription factors FOXA1 and FOXA2. a) A Venn diagram showed that 46 out of the 554 upregulated transcription factors during postnatal development were downregulated in the livers of *Ythdc1‐*HKO mice. b,c) ATAC‐seq analysis was performed on 9‐week‐old *Ythdc1* ^flox/flox^ and *Ythdc1‐*HKO mice. The top GO biological process terms enriched in genes associated with downregulated and upregulated peaks were identified, respectively. d) Significantly differential transcription factor (TF) binding motifs were identified by analyzing ATAC‐seq data from livers of 9‐week‐old *Ythdc1* ^flox/flox^ and *Ythdc1‐*HKO mice. e) *Foxa1* and *Foxa2* mRNA levels in the livers of 9‐week‐old *Ythdc1* ^flox/flox^ and *Ythdc1‐*HKO mice were measured by RT‐qPCR (*n* = 12 per group). f,g) FOXA1, FOXA2, and Tubulin protein levels in the livers of 9‐week‐old *Ythdc1* ^flox/flox^ and *Ythdc1‐*HKO mice were measured by immunoblotting and quantified using ImageJ (*n* = 7 per group). h) *Foxa1* and *Foxa2* mRNA levels in the livers of *Ythdc1* ^flox/flox^‐βGal and *Ythdc1‐*adultHKO mice were measured by RT‐qPCR (*n* = 5 per group). i,j) FOXA1, FOXA2, and Tubulin protein levels in the livers of *Ythdc1* ^flox/flox^‐βGal and *Ythdc1‐*adultHKO mice were determined by immunoblotting and quantified using ImageJ (*n* = 5 per group). *n* was the number of biologically independent mice. Data represent the mean ± SEM. The Shapiro–Wilk test was employed to assess the normality of the data. When both groups were normally distributed (*P* > 0.05), the parametric two‐tailed Student's *t*‐tests were used to detect the statistical differences between the two groups. When at least one of the two groups were not normally distributed (*P* < 0.05), the nonparametric Mann–Whitney test was adopted to compare the statistical differences between the two groups. *, *P* < 0.05. **, *P* < 0.01.

Next, we investigate whether YTHDC1 influences specific transcription factors and their targeted genes. RNA‐seq analysis revealed that 46 out of the 554 upregulated transcription factors during postnatal development were downregulated in the livers of *Ythdc1‐*HKO mice (**Figure** **6**a). To identify which transcription factors are associated with the downregulated hepatic genes in the livers of *Ythdc1‐*HKO mice, we performed Assay for Transposase‐Accessible Chromatin with high‐throughput sequencing (ATAC‐seq) to map open chromatin in their livers. As expected, we observed strong enrichment of open chromatin at gene promoters and ATAC‐seq peaks near the transcription start sites, which are associated with gene transcription activation (Figure S8a,b, Supporting Information). ATAC‐seq data analysis identified 2357 upregulated peak‐related genes and 4467 downregulated peak‐related genes (Table S3, Supporting Information). GO analysis showed that downregulated peak‐related genes were linked to processes such as small molecule metabolism, organic acid metabolism, developmental processes, biosynthesis, and anatomical structure morphogenesis (Figure 6b). In contrast, upregulated peak‐related genes were associated with processes such as regulation of cell adhesion, locomotion, developmental processes, cytokine production, immune system regulation, actin cytoskeleton organization, cell migration, and response to stimuli (Figure 6c). Motif analysis using HOMER identified the binding motifs of several transcription factors (Figure 6d). Enrichment analysis revealed that the binding motifs of FOXA1 and FOXA2 were less abundant in *Ythdc1*‐HKO livers compared to *Ythdc1* ^flox/flox^ livers (Figure 6d), whereas the binding motif of HNF4α showed similar enrichment in both *Ythdc1*‐HKO and *Ythdc1* ^flox/flox^ livers (Figure 6d). Both RNA‐seq and ATAC‐seq analyses revealed that 326 downregulated peak‐associated genes exhibited decreased mRNA levels in *Ythdc1*‐HKO livers, while 236 upregulated peak‐associated genes showed increased mRNA levels (Figure S9a–d, Supporting Information). Of the 326 downregulated genes, 123 were FOXA1‐targeted genes, and 196 were FOXA2‐targeted genes (Figure S9a,b, Supporting Information). Among the 236 upregulated genes, 71 were FOXA1‐targeted genes, and 118 were FOXA2‐targeted genes (Figure S9c,d, Supporting Information). RT‐qPCR and immunoblotting confirmed the downregulation of both mRNA and protein levels of FOXA1 and FOXA2 in the livers of *Ythdc1*‐HKO mice (Figure 6e–g). Similarly, adult‐onset hepatocyte‐specific deletion of *Ythdc1* also significantly reduced both mRNA and protein levels of FOXA1 and FOXA2 (Figure 6h–j). Expression of HNF4α remained unchanged in both *Ythdc1*‐HKO and *Ythdc1*‐adultHKO livers (Figure S10a–f, Supporting Information), suggesting that YTHDC1 does not regulate HNF4α expression and that HNF4α may not be involved in YTHDC1‐mediated regulation of liver function. Interestingly, FOXA1 and FOXA2 were upregulated during postnatal liver development (Figure S11a–f, Supporting Information). The expression of *Foxa1*, *Foxa2*, the liver marker Albumin, gluconeogenesis‐related genes (e.g., *G6pase*, *Pepck*), and cytochrome P450 enzymes (e.g., Cyp2a4, Cyp2e1) in the livers of 3‐week‐old *Ythdc1*‐HKO mice was significantly decreased (Figure S12, Supporting Information), indicating that YTHDC1 plays a critical role in regulating gene expression as early as 3 weeks of age, highlighting its essential function at this developmental stage. These findings also suggest that the downregulation of FOXA1 and FOXA2 may contribute to the phenotypes observed in *Ythdc1*‐HKO mice.

### 2.7 Hepatocyte‐Specific Overexpression of FOXA1 or FOXA2 Rescues Impaired Liver Function in *Ythdc1*‐HKO Mice

**Figure 7:**
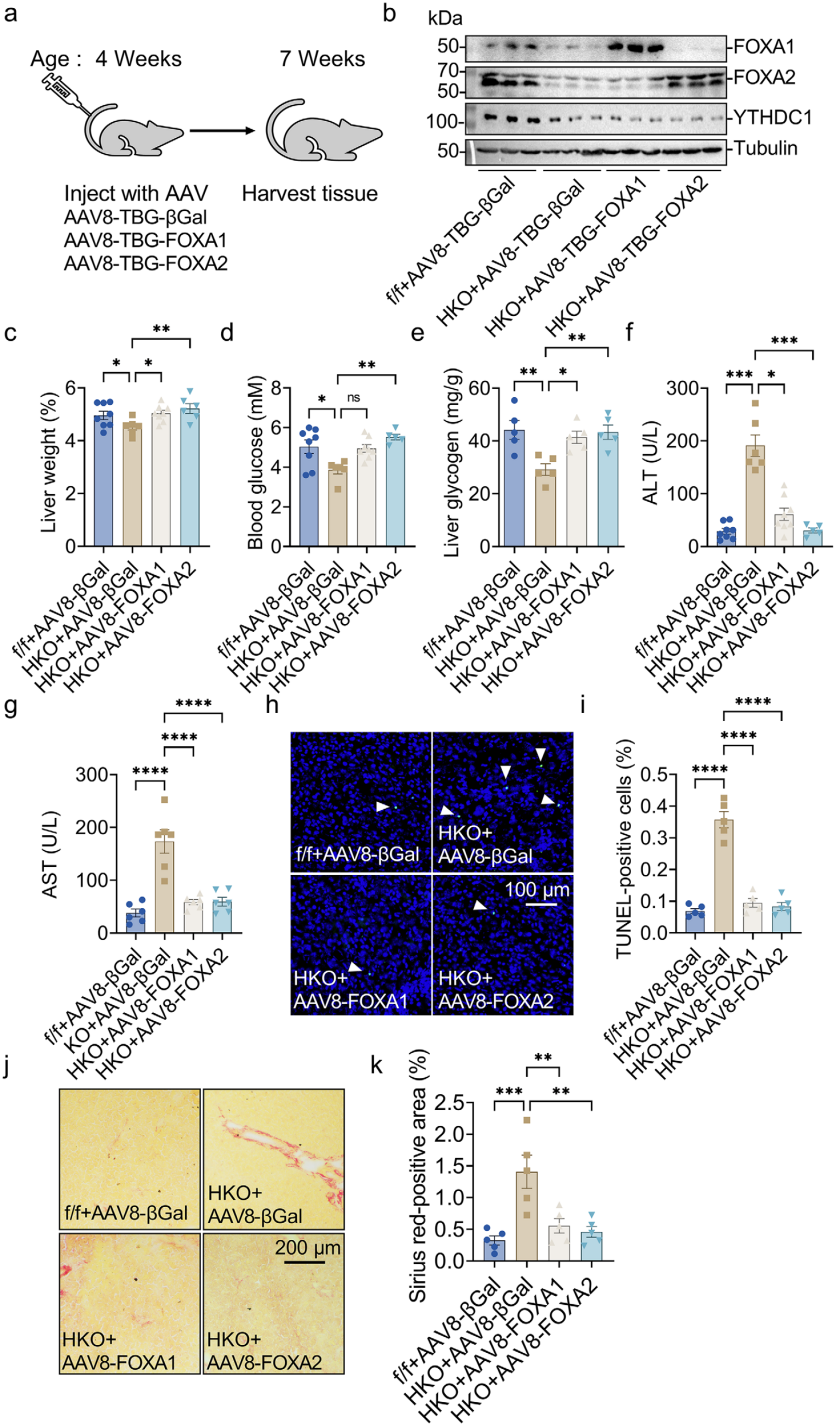
Hepatocyte‐specific overexpression of FOXA1 or FOXA2 rescues impaired liver function in *Ythdc1*‐HKO mice. a) Four‐week‐old male *Ythdc1* ^flox/flox^ mice were injected with AAV8‐TBG‐βGal (2 × 10^11^ vp per mouse) via tail vein. Four‐week‐old male *Ythdc1‐*HKO mice were injected with equal amounts of AAV8–TBG–βGal, AAV8–TBG–FOXA1, or AAV8–TBG–FOXA2 via tail vein. Phenotypes were measured, and mice were sacrificed three weeks later. b) FOXA1, FOXA2, YTHDC1, and Tubulin protein levels were measured by immunoblotting. c) Liver weights were measured (*n* = 6–8 per group). d,e) Blood glucose and liver glycogen levels were determined (*n* = 5–8 per group). f,g) Serum ALT and AST activities were measured (*n* = 6–8 per group). h,i) TUNEL‐positive cells were quantified (*n* = 5 per group). j,k) Sirius Red staining was performed on liver sections (*n* = 5 per group). *n* was the number of biologically independent mice. Data represent the mean ± SEM. The Shapiro–Wilk test was employed to assess the normality of the data. When all groups were normally distributed (*P* > 0.05), the parametric one‐factor analysis of variance (ANOVA), and Tukey was used to detect the statistical differences. When at least one of the two groups was not normally distributed (*P* < 0.05), the nonparametric Kruskal–Wallis and Dunn's was adopted to compare the statistical differences. *, *P* < 0.05. **, *P* < 0.01. ***, *P* < 0.001. ****, *P* < 0.0001.

To confirm that the observed defects were due to decreased expression of FOXA1 or FOXA2 in *Ythdc1*‐HKO mice, we evaluated whether re‐expressing FOXA1 or FOXA2 in the liver of 4‐week‐old *Ythdc1*‐HKO mice could rescue these phenotypes. We administered adeno‐associated virus (AAV) vectors expressing FOXA1, FOXA2, or a control βGal (AAV‐βGal) under a hepatocyte‐specific TBG promoter via tail‐vein injection (**Figure** **7**a), resulting in selective re‐expression of FOXA1 or FOXA2 in the liver (Figure 7b). Re‐expression of FOXA1 or FOXA2 in *Ythdc1*‐HKO mice significantly rescued liver phenotypes, as demonstrated by increased liver weight (Figure 7c), improved glucose homeostasis (elevated blood glucose and liver glycogen levels) (Figure 7d,e), restored expression of gluconeogenesis‐related genes (*Gcgr*, *G6pase*, and *Pepck*) (Figure S13a, Supporting Information), and a substantial reduction in liver injury and fibrosis, as indicated by decreased serum ALT and AST activities (Figure 7f,g), downregulated expression of inflammation‐related genes (e.g., *Cxcl5*, *Ccl22*, and *Il1b*) (Figure S13b, Supporting Information), reduced TUNEL‐positive cells (Figure 7h,i), and smaller Sirius Red‐positive areas (Figure 7j,k).

To further determine whether the downregulation of FOXA1 and FOXA2‐targeted genes could be rescued by overexpression of FOXA1 or FOXA2 in *Ythdc1*‐HKO livers, we conducted RNA‐seq analysis on four groups of mouse livers. As shown in Figure S13c,d (Supporting Information), 271 downregulated genes were restored in FOXA1‐overexpressing *Ythdc1*‐HKO livers, of which 73 were FOXA1‐targeted genes. In contrast, 1103 downregulated genes were rescued in FOXA2‐overexpressing *Ythdc1*‐HKO livers, with 448 of these being FOXA2‐targeted genes. RT‐qPCR analysis further validated these findings, confirming that the expression of FOXA1‐targeted genes, such as *C2*, *C6*, *C8b*, *Igf1*, *Nrp1*, and *Cy4a12a*, was restored in FOXA1‐overexpressing *Ythdc1*‐HKO livers (Figure S13e, Supporting Information). Similarly, the expression of FOXA2‐targeted genes, including *C8b*, *C9*, *Cfh*, *Sirt5*, *Rorc*, *F7*, and *Abcg8*, was restored in FOXA2‐overexpressing *Ythdc1*‐HKO livers (Figure S13f, Supporting Information). These results suggest that the downregulation of FOXA1 and FOXA2 contributes to the impaired postnatal liver development observed in *Ythdc1*‐HKO mice.

### 2.8 YTHDC1, But Not YTHDC1 W378A Mutation, Fully Rescues Impaired Liver Function in *Ythdc1*‐HKO Mice

**Figure 8:**
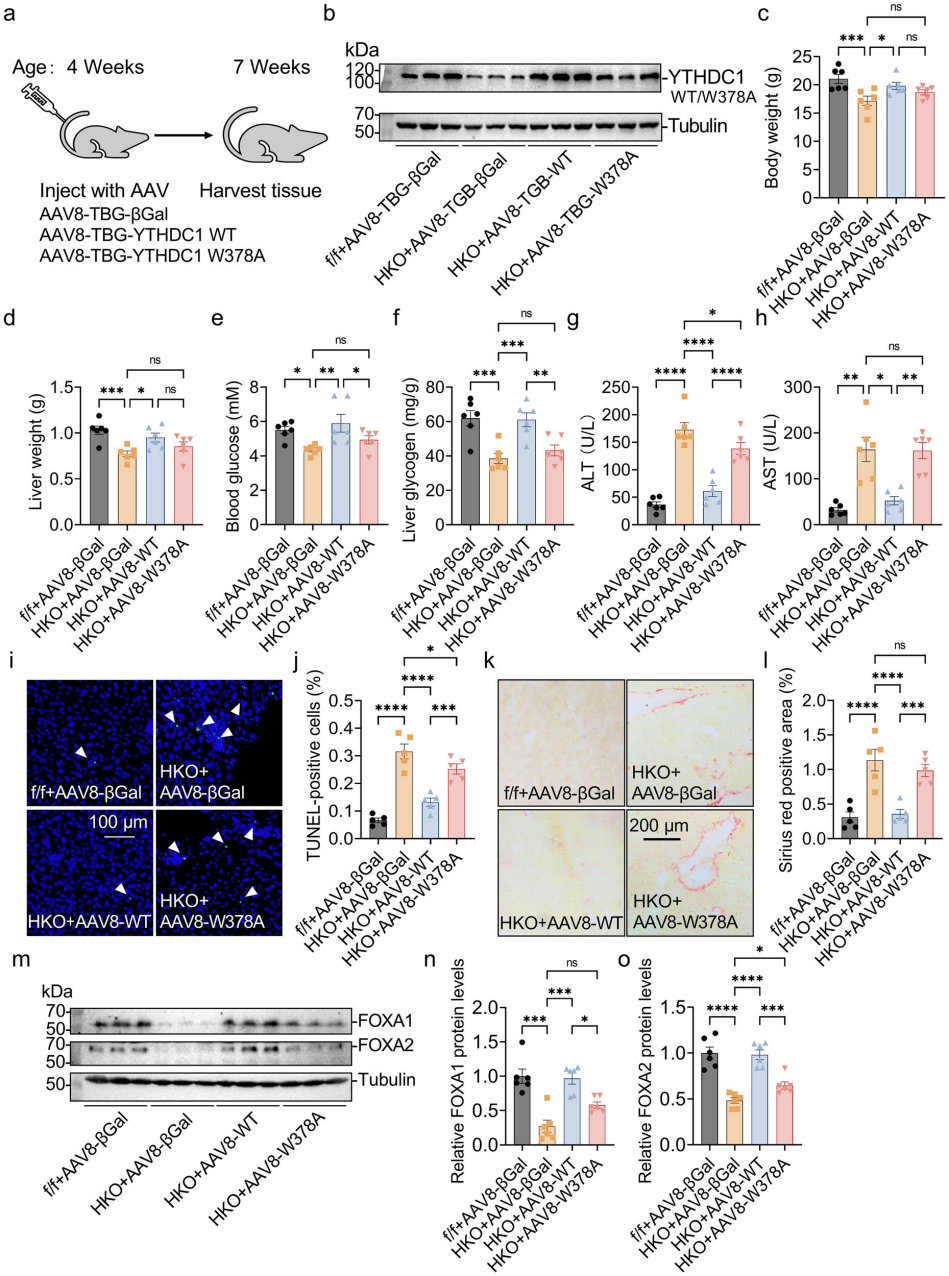
YTHDC1, but not YTHDC1 W378A mutation, fully rescues the impaired liver function in *Ythdc1*‐HKO mice. a) Four‐week‐old male *Ythdc1* ^flox/flox^ mice were injected with AAV8‐TBG‐βGal (2 × 10^11^ vp per mouse) via tail vein. Four‐week‐old male *Ythdc1‐*HKO mice were injected with equal amounts of AAV8–TBG–βGal, AAV8–TBG–YTHDC1 (WT), or AAV8–TBG–YTHDC1(W378A) via tail vein. Phenotypes were measured, and mice were sacrificed three weeks later. b) YTHDC1 and Tubulin protein levels were measured by immunoblotting (*n* = 3 per group). c,d) Body and liver weights were measured (*n* = 6 per group). e,f) Blood glucose and liver glycogen levels were determined (*n* = 6 per group). g,h) Serum ALT and AST activities were determined (*n* = 6 per group). i,j) TUNEL‐positive cells were quantified (*n* = 5 per group). k,l) Sirius Red staining was performed on liver sections (*n* = 5 per group). m–o) FOXA1, FOXA2, and Tubulin protein levels were measured by immunoblotting and quantified using ImageJ (*n* = 3 for representative, *n* = 6 for quantification). *n* was the number of biologically independent mice. Data represent the mean ± SEM. The Shapiro–Wilk test was employed to assess the normality of the data. When all groups were normally distributed (*P* > 0.05), the parametric one‐factor analysis of variance (ANOVA), and Tukey was used to detect the statistical differences. When at least one of the two groups was not normally distributed (*P* < 0.05), the nonparametric Kruskal–Wallis and Dunn's was adopted to compare the statistical differences. *, *P* < 0.05. **, *P* < 0.01. ***, *P* < 0.001. ****, *P* < 0.0001.

YTHDC1, a key RNA m^6^A reader protein, plays a crucial role in regulating various RNA processing steps.^[^ ^11^, ^12^, ^13^ ^]^ W377 residue in YTHDC1 (corresponding to W378 in mice) is essential for m^6^A recognition.^[^ ^12^ ^]^ It has been shown that the YTHDC1 W377A mutation disrupts m^6^A binding.^[^ ^12^, ^27^ ^]^ To assess whether m^6^A recognition by YTHDC1 is necessary for promoting postnatal liver development, we investigated whether re‐expressing YTHDC1 or the YTHDC1 W378A mutant in the liver of 4‐week‐old *Ythdc1*‐HKO mice could rescue the phenotypes. AAV vectors expressing YTHDC1 (AAV8–TBG–YTHDC1), YTHDC1 W378A (AAV8–TBG–W378A) or a control βGal (AAV8–TBG–βGal) were administered via tail‐vein injection (**Figure** **8**a), resulting in selective liver re‐expression (Figure 8b). Re‐expression of YTHDC1 in *Ythdc1*‐HKO mice significantly promoted postnatal liver development, as evidenced by increased body and liver weight (Figure 8c,d), elevated blood glucose and liver glycogen levels (Figure 8e,f), and reduced liver injury and fibrosis, indicated by decreased serum ALT and AST activities (Figure 8g,h), fewer TUNEL‐positive cells (Figure 8i,j), and reduced Sirius Red‐positive areas (Figure 8k,l). These improvements were likely due to the increased expression of FOXA1 and FOXA2 (Figure 8m–o). In contrast, re‐expression of the YTHDC1 W378A mutant in *Ythdc1*‐HKO mice only partially rescued liver development and function (Figure 8c–l), likely due to the partial restoration of FOXA1 and FOXA2 expression (Figure 8m–o). These findings suggest that m^6^A recognition by YTHDC1 is essential for proper postnatal liver development.

To further investigate the role of m^6^A recognition by YTHDC1 in maintaining liver function, we tested whether re‐expressing YTHDC1 or the YTHDC1 W378A mutant in adult‐onset hepatocyte‐specific *Ythdc1* knockout (*Ythdc1*‐adultHKO) mice could rescue the observed phenotypes. *Ythdc1*‐adultHKO mice were generated by tail‐vein injection of purified AAV8–TBG–Cre into adult *Ythdc1* ^flox/flox^ mice. Re‐expression of YTHDC1 or YTHDC1 W378A was achieved by tail‐vein injection of purified AAV8–TBG–YTHDC1 and AAV8–TBG–Cre or AAV8–TBG–YTHDC1(W378A) and AAV8–TBG–Cre, with AAV8–TBG–βGal used as a control (Figure S14a, Supporting Information). *Ythdc1*‐adultHKO mice displayed significantly decreased YTHDC1 protein levels, while AAV8–TBG–YTHDC1 or AAV8–TBG–YTHDC1(W378A) successfully re‐expressed YTHDC1 or the YTHDC1(W378A) mutation in *Ythdc1*‐adultHKO mice (Figure S14b,c, Supporting Information). Consistent with the data in Figure 3, *Ythdc1*‐adultHKO mice exhibited normal liver weights (Figure S14d, Supporting Information), but showed impaired glucose homeostasis and liver injury (Figure S14e–o, Supporting Information). Re‐expression of YTHDC1 in *Ythdc1*‐adultHKO mice largely restored liver function and prevented injury, as evidenced by increased blood glucose, enhanced hepatic glucose production, and elevated liver glycogen levels (Figure S14e–j, Supporting Information), along with reduced serum ALT activity (Figure S14k, Supporting Information), decreased serum AST activity (Figure S14l, Supporting Information), a smaller injury area (Figure S14m, Supporting Information), and a lower number of TUNEL‐positive cells (Figure S14n,o, Supporting Information), likely due to the upregulation of *Foxa1* and *Foxa2* expression (Figure S14p,q, Supporting Information). In contrast, re‐expression of the YTHDC1(W378A) mutant in *Ythdc1*‐adultHKO mice did not rescue liver function or prevent injury (Figure S14e–o, Supporting Information), as it failed to increase *Foxa1* and *Foxa2* expression (Figure S14p,q, Supporting Information). These results confirm that m^6^A recognition by YTHDC1 is essential for maintaining liver function.

### 2.9 YTHDC1 Directly Binds to m^6^A‐Modified *Foxa1*/*Foxa2* mRNA and Is Essential for Their mRNA Stability in the Nucleus and for Their Transport from the Nucleus to the Cytosol

**Figure 9:**
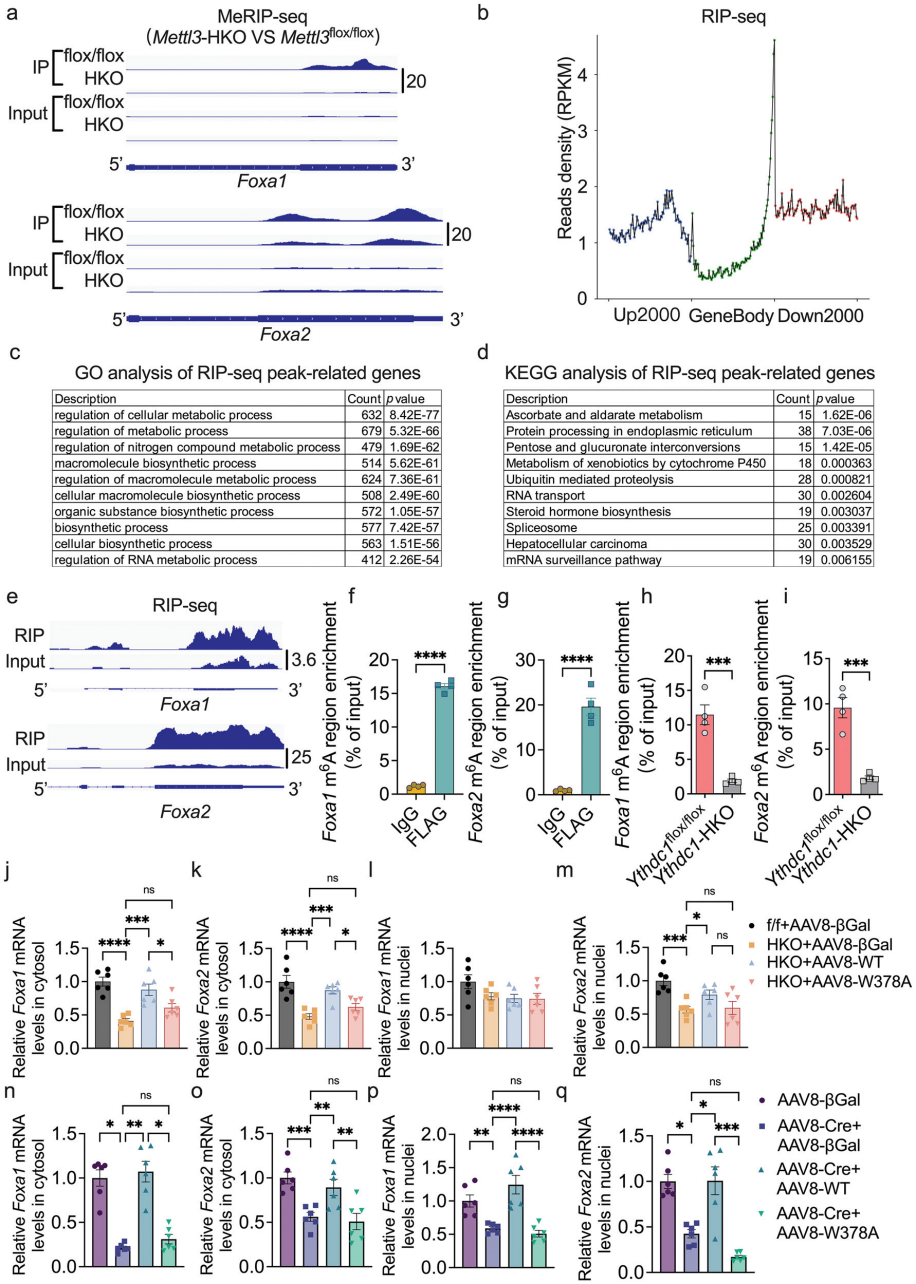
YTHDC1 directly binds to *Foxa1*/*Foxa2* mRNA and is required for nuclear–cytosolic translocation of *Foxa1/2* mRNA. a) The read density from MeRIP‐seq experiment comparing *Mettl3*‐HKO to *Mettl3* ^flox/flox^ and *Wtap*‐HKO to *Wtap* ^flox/flox^ shows the m^6^A peaks identified in *Foxa1* and *Foxa2* transcripts. b) Primary hepatocytes were infected overnight with Ad‐FLAG‐YTHDC1 adenovirus. Ribonucleoprotein immunoprecipitation sequencing (RIP‐seq) was conducted. The distribution of RIP‐seq peaks was analyzed in up2000, GeneBody, and Down2000 regions. c) GO analysis of RIP‐seq peak‐related genes was conducted. d) KEGG analysis of RIP‐seq peak‐related genes was performed. e) The read density from YTHDC1 RIP‐seq experiment showing the peaks identified in the *Foxa1* and *Foxa2* transcripts. f,g) Primary hepatocytes from C57BL/6 WT mice were infected overnight with Ad‐βGal or Ad‐FLAG‐YTHDC1. RIP‐RT‐qPCR was performed (*n* = 4 per group). h,i) Ribonucleoprotein immunoprecipitation (RIP) was conducted in *Ythdc1*‐HKO to *Ythdc1* ^flox/flox^ livers using anti‐YTHDC1 antibody. RIP‐RT‐qPCR was then performed (*n* = 4 per group). j–m) Four‐week‐old male *Ythdc1* ^flox/flox^ mice were injected with AAV8‐TBG‐βGal (2 × 10^11^ vp per mouse) via tail vein. Four‐week‐old male *Ythdc1‐*HKO mice were injected with equal amounts of AAV8–TBG–βGal, AAV8–TBG–YTHDC1 (WT), or AAV8–TBG–YTHDC1(W378A) via tail vein. Mice were sacrificed three weeks later, and liver tissues were collected. Cytosolic and nuclear RNA were isolated, and *Foxa1* and *Foxa2* mRNA levels were measured by RT‐qPCR (*n* = 6 per group). n–q) Adult‐onset hepatocyte‐specific *Ythdc1* knockout (*Ythdc1*‐adultHKO) mice were generated by tail‐vein injection of purified AAV8–TBG–Cre virus into adult *Ythdc1* ^flox/flox^ mice. Re‐expressing YTHDC1 or YTHDC1 W378A was achieved by tail‐vein injection of purified AAV8–TBG–YTHDC1 and AAV8–TBG–Cre or AAV8–TBG–YTHDC1(W378A) and AAV8–TBG–Cre virus into adult *Ythdc1* ^flox/flox^ mice. Adult *Ythdc1* ^flox/flox^ mice injected with equal amounts of AAV8–TBG–βGal virus served as the control. Sixteen days later, mice were sacrificed, and liver tissues were collected. Cytosolic and nuclear *Foxa1* and *Foxa2* mRNA levels were measured by RT‐qPCR (*n* = 6 per group). Data represent the mean ± SEM. The Shapiro–Wilk test was employed to assess the normality of the data. When all groups were normally distributed (*P* > 0.05), the parametric two‐tailed Student's *t*‐tests were used to detect the statistical differences between the two groups. The parametric one‐factor analysis of variance (ANOVA), and Tukey was used to detect the statistical differences among four groups. When at least one of the two groups were not normally distributed (*P* < 0.05), the nonparametric Mann–Whitney test was adopted to compare the statistical differences between the two groups. The nonparametric Kruskal–Wallis and Dunn's was adopted to compare the statistical differences among four groups. *, *P* < 0.05. **, *P* < 0.01.

The m^6^A modification of *Foxa1* and *Foxa2* transcripts appears crucial for maintaining their expression. The m^6^A writer proteins METTL3 and WTAP are likely essential for introducing m^6^A modification in these transcripts. We reanalyzed our previous MeRIP‐seq data comparing *Mettl3*‐HKO with *Mettl3* ^flox/flox^, as well as *Wtap*‐HKO with *Wtap* ^flox/flox^ livers.^[^ ^7^, ^8^ ^]^ As shown in **Figure** **9**a; Figure S15a (Supporting Information), m^6^A modifications were detected in the 3′‐UTR regions of both *Foxa1* and *Foxa2* transcripts. Hepatocyte‐specific deletion of *Mettl3* or *Wtap* resulted in reduced m^6^A modification levels in these regions (Figure 9a; Figure S15a, Supporting Information), which corresponded with the downregulation of *Foxa1* and *Foxa2* transcripts in both *Mettl3*‐HKO and *Wtap*‐HKO livers (Figure S15b,c, Supporting Information). YTHDC1 did not affect the expression of *Mettl3* or *Wtap* (Figure S15d,e, Supporting Information) or the m^6^A levels of *Foxa1* and *Foxa2* (Figure S15f, Supporting Information).

To investigate whether YTHDC1 directly binds to these mRNAs, particularly *Foxa1* and *Foxa2* transcripts, we performed ribonucleoprotein immunoprecipitation sequencing (RIP‐seq). As shown in Table S4 (Supporting Information), RIP‐seq analysis identified 1641 peak‐related transcripts, with peaks predominantly enriched at the STOP site and 3′‐UTR (Figure 9b; Figure S16a, Supporting Information). GO analysis revealed that these transcripts were associated with regulation of cellular metabolic process, macromolecule biosynthetic process, regulation of RNA metabolic process (Figure 9c). KEGG analysis showed that these 1641 peak‐related genes were involved in pathways such as ascorbate and aldarate metabolism, protein processing in endoplasmic reticulum, metabolism of xenobiotics by cytochrome P450, RNA transport, spliceosome, hepatocellular carcinoma, and mRNA surveillance pathway (Figure 9d). Among the 1641 peak‐related transcripts were *Foxa1* and *Foxa2* (Table S4, Supporting Information), with binding sites closely aligned with m^6^A modification regions (Figure 9e), which promoted us to follow up on *Foxa1* and *Foxa2*.

RIP‐RT‐qPCR data confirmed that YTHDC1 directly binds to the m^6^A‐modified regions in the *Foxa1* and *Foxa2* transcripts in both YTHDC1‐overexpressing hepatocytes (Figure 9f,g) and *Ythdc1* ^flox/flox^ livers, but not in *Ythdc1*‐HKO livers (Figure 9h,i). RNA pull‐down experiments showed that both endogenous and exogenous YTHDC1 bound to the m^6^A‐modified probes of *Foxa1* and *Foxa2*, but not to the m^6^A‐unmodified probes (Figure S16b–e, Supporting Information). YTHDC1 is known to recognize m^6^A modifications and mediates the nuclear decay and nuclear–cytosolic mRNA translocation.^[^ ^13^ ^]^ We showed that YTHDC1 interacted with SRSF3 (a nuclear export adaptor protein) (Figure S17a, Supporting Information), which is consistent with previous observation.^[^ ^13^ ^]^

To explore this role further, we measured mRNA levels in both the cytosol and nucleus. As shown in Figure S17b,c,e–g,i (Supporting Information), cytosolic mRNA levels of *Foxa1* and *Foxa2*, as well as nuclear mRNA levels of *Foxa2*, were significantly decreased in the livers of both embryonic‐ and adult‐onset hepatocyte‐specific *Ythdc1* knockout mice. Notably, nuclear mRNA levels of *Foxa1* were significantly decreased in adult‐onset, but not embryonic‐onset, *Ythdc1* knockout mice (Figure S17d,h, Supporting Information). Moreover, the proportion of cytosolic *Foxa1* and *Foxa2* mRNAs was significantly reduced in the livers of both embryonic‐ and adult‐onset hepatocyte‐specific *Ythdc1* knockout mice, while the proportion of nuclear *Foxa1* and *Foxa2* mRNAs was significantly increased (Figure S17j–q, Supporting Information). This suggests that hepatic deletion of *Ythdc1* impairs the transport of these transcripts from the nucleus to the cytosol. Reintroduction of YTHDC1, but not the YTHDC1 W378A mutant, restored both cytosolic and nuclear levels of *Foxa1* and *Foxa2* mRNAs in *Ythdc1*‐HKO and *Ythdc1*‐adultHKO mice (Figure 9j–q). Additionally, YTHDC1 reintroduction, but not the W378A mutant, also corrected the proportion of cytosolic *Foxa1* and *Foxa2* mRNAs by decreasing the proportion of nuclear *Foxa1* and *Foxa2* mRNAs in both *Ythdc1*‐HKO and *Ythdc1*‐adultHKO mice (Figure S18a–h, Supporting Information).

These findings suggest that YTHDC1 directly binds to the m^6^A‐modified regions of *Foxa1* and *Foxa2* transcripts and is critical for their post‐transcriptional processing, including nuclear RNA decay and nuclear–cytosolic mRNA translocation.

## 3 Discussion

The neonatal liver is relatively immature and undergoes a highly coordinated postnatal development process to achieve full functionality. This developmental immaturity can make neonates more susceptible to various liver diseases.^[^ ^1^, ^2^ ^]^ Understanding the molecular mechanisms driving postnatal liver development is crucial. In this study, we compared gene expression profiles during this developmental period and identified the m^6^A reader protein YTHDC1 as essential for proper postnatal liver development. YTHDC1 is upregulated throughout this process, and hepatocyte‐specific deletion of *Ythdc1* results in impaired liver development, leading to liver injury, inflammation, fibrosis, and pathological changes characteristic of NASH and HCC in mice. YTHDC1 promotes postnatal liver development by enhancing the post‐transcriptional expression of FOXA1 and FOXA2 through m^6^A recognition.

Postnatal liver development is tightly regulated. RNA‐seq analysis shows that genes related to various metabolic processes are gradually upregulated, while those involved in rRNA processing, chromosome segregation, erythrocyte homeostasis, cytoplasmic translation, and cytokine production are progressively downregulated. This regulatory pattern enables the liver to acquire its full range of functions, including the production of albumin, bile acids, and essential nutrients, maintenance of blood glucose, amino acid, and lipid levels, and the detoxification of harmful substances from the bloodstream. TFs and RBPs likely play pivotal roles in this process. Notably, the m^6^A reader proteins YTHDC1 and YTHDF2/3, which are undetected during embryonic development, are significantly upregulated during postnatal liver development, suggesting their critical regulatory roles.

Hepatocyte‐specific deletion of *Ythdc1* impairs postnatal liver development, leading to smaller liver size, reduced liver glycogen levels, and lower body weight starting at four weeks of age. Adult *Ythdc1*‐HKO mice exhibit impaired glucose homeostasis, severe liver injury, inflammation, and fibrosis, which further promote the pathogenesis of NASH and HCC. Similarly, adult‐onset hepatocyte‐specific *Ythdc1* knockout mice show impaired glucose homeostasis, severe liver injury, inflammation, and fibrosis, but no changes in liver or body weight. These findings suggest that YTHDC1 may have distinct roles at different stages of liver development. In adult liver, YTHDC1 likely contributes to maintaining homeostasis and regulating the function of mature hepatocytes. In contrast, during postnatal development, YTHDC1 may influence liver progenitors and liver growth, potentially accounting for the reduced liver size observed in *Ythdc1*‐HKO mice. Additionally, YTHDC1 may be involved in hepatocyte maturation and the establishment of liver function. The phenotypes observed in *Ythdc1*‐HKO mice are likely due to the immaturity of hepatocytes, which are unable to perform normal functions such as dietary compound metabolism, blood glucose regulation, clotting factor production, bile synthesis, and biotransformation of xenobiotics and metabolic by‐products. Consistent with this, metabolism‐related genes are downregulated, while genes related to angiogenesis, extracellular matrix organization, positive regulation of cell motility, chemotaxis, response to wounding, T cell activation, mitotic sister chromatid segregation, positive regulation of cytokine production, and actin filament organization are upregulated. Among the 554 upregulated transcription factors during postnatal development, 46 were downregulated in *Ythdc1*‐HKO livers, including FOXA. FOXA transcription factors, particularly FOXA1 and FOXA2, are critical for liver development and function.^[^ ^3^, ^28^ ^]^ ATAC‐seq analysis indicates that the binding motifs of FOXA1 and FOXA2 are less abundant in *Ythdc1*‐HKO livers. Analysis of RNA‐seq, ATAC‐seq, and FOXA1/2 ChIP‐seq datasets reveals partial overlap, suggesting that YTHDC1 regulates gene expression, at least in part, through FOXA1 and FOXA2. However, re‐expression of FOXA1 or FOXA2 in four‐week‐old *Ythdc1*‐HKO mice rescues the observed phenotypes, demonstrating that downregulation of FOXA1 and FOXA2 contributes to the impaired liver development and function in *Ythdc1*‐HKO mice. ATAC‐seq analysis also shows that chromatin accessibility at the promoter regions of *Foxa1* and *Foxa2* remains unchanged in *Ythdc1*‐HKO livers, indicating that YTHDC1 regulates *Foxa1* and *Foxa2* expression at the post‐transcriptional level. YTHDC1 directly binds to m^6^A‐modified regions of *Foxa1* and *Foxa2* transcripts and mediates their posttranscriptional RNA processing. Full phenotypic rescue is observed in both embryonic‐ and adult‐onset hepatocyte‐specific *Ythdc1* knockout mice upon re‐expression of YTHDC1, but not with the YTHDC1 W378A mutant. This finding underscores the necessity of m^6^A recognition for YTHDC1's liver function. The role of m^6^A recognition by YTHDC1 is also crucial in other cell types; for example, YTHDC1 W378A mutant ES cells exhibit phenotypes similar to *Ythdc1*‐CKO ES cells.^[^ ^27^, ^29^ ^]^ The function and regulated genes of YTHDC1 and METTL3 in islet β cells are similar,^[^ ^29^, ^30^ ^]^ suggesting that YTHDC1 and METTL3 may operate through a similar pathway, likely involving m^6^A mRNA modification. Additionally, the partial phenotypic rescue observed in embryonic‐onset, but not in adult‐onset, hepatocyte‐specific *Ythdc1* knockout mice with YTHDC1 W378A suggests that mechanisms beyond m^6^A recognition may also contribute to postnatal liver development. YTHDC1 directly binds to 1641 transcripts related to metabolism, RNA transport, the spliceosome, and the mRNA surveillance pathway. It is possible that YTHDC1 may influence the fate of specific transcripts independently of its m^6^A recognition. Furthermore, nuclear RNA‐binding proteins often bind to chromatin as well. In the liver, both METTL3 and WTAP can bind to gene promoters and regulate chromatin accessibility.^[^ ^7^, ^8^ ^]^ Whether YTHDC1 regulates gene expression by binding to chromatin warrants further investigation.

m^6^A‐related proteins play a critical role in regulating liver function. Our current and previous studies demonstrate that the deficiency of *Ythdc1*, *Mettl3*, or *Wtap* in the liver leads to liver injury and inflammation,^[^ ^8^, ^20^ ^]^ contributing to the pathogenesis of NASH and HCC,^[^ ^7^, ^8^, ^21^, ^31^ ^]^ indicating that maintaining the expression or activity of YTHDC1, METTL3, and WTAP by small molecules or genetic methods might be an approach to ameliorate liver injury and NASH. In contrast, our recent study demonstrated that the deletion of *Alkbh5* (the gene encoding ALKBH5, an m^6^A eraser protein) in the liver enhances glucose and lipid homeostasis while maintaining normal liver function.^[^ ^32^ ^]^ Additionally, targeted inhibition of hepatic ALKBH5 prevents HFD‐induced liver injury and steatosis,^[^ ^32^ ^]^ offering an alternative therapeutic approach.

YTHDC1, METTL3, and WTAP are essential for maintaining liver function, and their dysregulation leads to liver injury and inflammation, contributing to the pathogenesis of both NASH and HCC. However, their molecular mechanisms differ. METTL3 and WTAP support liver function through both m^6^A‐dependent and m^6^A‐independent mechanisms.^[^ ^7^, ^8^, ^20^, ^21^, ^31^, ^33^ ^]^ While RNA‐seq data from *Mettl3*‐HKO and *Wtap*‐HKO livers, along with other studies, show that hepatic deficiency of *Mettl3* or *Wtap* decreases the expression of HNF4α,^[^ ^8^, ^20^, ^33^ ^]^ hepatic deletion of *Ythdc1* does not affect HNF4α expression. Other m^6^A readers, such as YTHDF2 and YTHDF3, may recognize m^6^A methylation on *Hnf4a* and contribute to liver development. Instead, hepatic deficiency of *Ythdc1* significantly reduces the expression of FOXA1 and FOXA2 by impairing their mRNA transport from the nucleus to the cytosol. YTHDC1 binds to m^6^A‐modified *Foxa1* and *Foxa2* transcripts and exerts its function in the liver through m^6^A recognition, without affecting their m^6^A modification levels. In contrast, hepatic loss of *Mettl3* or *Wtap* decreases both m^6^A modification and the expression of *Foxa1* and *Foxa2*. These findings demonstrate that m^6^A writers METTL3 and WTAP add m^6^A modifications to *Foxa1* and *Foxa2* transcripts, while the m^6^A reader YTHDC1 recognizes these modifications and facilitates their nucleus‐to‐cytosol transport. Collectively, our results highlight the crucial role of m^6^A modifications in maintaining the expression of *Foxa1* and *Foxa2*.

*Mettl3* and *Mettl14* expression declines and global m^6^A levels significantly drop starting from the third week,^[^ ^33^, ^34^ ^]^ while the m^6^A reader proteins YTHDC1 and YTHDF2/3, which are undetected during embryonic development, are significantly upregulated during postnatal liver development. Postnatal upregulation of the m^6^A reader proteins YTHDC1 and YTHDF2/3 may serve as a compensatory mechanism for the decrease in m^6^A methylation. We have demonstrated that YTHDC1 is essential for postnatal liver development and function in this study. Further investigation is needed to determine whether YTHDF2 and YTHDF3 play similar roles or compensate for the function of YTHDC1 in liver development and disease.

## 4 Conclusion

We have demonstrated that YTHDC1 is a critical regulator of hepatocyte maturation and plays a key role in maintaining liver homeostasis. Upregulated in the liver after birth, YTHDC1 promotes postnatal hepatocyte maturation by enhancing the posttranscriptional expression of FOXA1 and FOXA2 through m^6^A recognition. Hepatocyte‐specific deletion of *Ythdc1* results in hepatocyte immaturity, leading to reduced liver weight, liver injury, inflammation, and fibrosis, which in turn promotes the pathogenesis of NASH and HCC. Re‐expression of FOXA1 or FOXA2 rescues the phenotypes observed in *Ythdc1*‐HKO mice. Our study highlights the essential role of YTHDC1 in regulating postnatal liver development and function.

## 5 Experimental Section

### Animal Experiments

Animal experiments were carried out in strict accordance with the Guide for the Care and Use of Laboratory Animals. Animal experiment protocols were approved by the Institutional Animal Care and Use Committee (IACUC) of Harbin Institute of Technology (HIT/IACUC‐2018004). Mice were housed on a 12‐h light/12‐h dark cycle and were provided a normal chow diet with ad libitum access to water. For diet‐induced NASH, mice were fed an MCD (MD12052, Medicience) for 10 days. The *Ythdc1* ^flox/flox^ mice were provided by the Cambridge‐suda Genomic Resource Center, with exons 5–7 of the *Ythdc1* gene flanked by two loxp sites, as described previously.^[^ ^17^ ^]^ Hepatocyte‐specific *Ythdc1* knockout (*Ythdc1‐*HKO) mice were generated by crossing *Ythdc1* ^flox/flox^ mice with *Alb*‐Cre mice. Mice were sacrificed on day 10 after MCD feeding. For DEN‐induced HCC, two‐week‐old male *Ythdc1* ^flox/flox^, and *Ythdc1‐*HKO mice received a single intraperitoneal dose of DEN (50 mg kg^−1^) and were maintained on an NC diet. Mice were sacrificed at 8 months of age for HCC examination. To generate adult‐onset hepatocyte‐specific *Ythdc1* knockout (*Ythdc1*‐adultHKO) mice, 8‐week‐old *Ythdc1* ^flox/flox^ mice were injected with AAV8–TBG–Cre or AAV8–TBG–βGal (2 × 10^11^ vp per mouse) via tail vein for 16 days, as described previously.^[^ ^35^ ^]^ For rescue experiments, four‐week‐old male *Ythdc1* ^flox/flox^ mice were injected with AAV8–TBG–βGal (2 × 10^11^ vp per mouse) via tail vein. Four‐week‐old male *Ythdc1‐*HKO mice were injected with equal amounts of AAV8–TBG–βGal, AAV8–TBG–FOXA1, AAV8–TBG–FOXA2, AAV8–TBG–YTHDC1 (WT), or AAV8–TBG–YTHDC1(W378A) via tail vein. Phenotypes were measured, and mice were sacrificed three weeks later. In another set of rescue experiments, 8‐week‐old male *Ythdc1* ^flox/flox^ mice were injected with equal amounts of AAV8–TBG–βGal, AAV8–TBG–Cre and AAV8–TBG–βGal, AAV8–TBG–Cre and AAV8–TBG–YTHDC1, or AAV8–TBG–Cre and AAV8–TBG–YTHDC1(W378A) viruses via tail vein. Phenotypes were measured, and mice were sacrificed sixteen days later. Blood samples were collected from orbital sinus. The serum ALT activities were measured with an ALT reagent set.^[^ ^36^ ^]^ The serum AST activities were measured with an AST reagent set (C010‐2‐1, Nanjing Jiancheng Bioengineering Institute).

### Nuclear and Cytosolic Extract Preparation

Nuclei isolation was following a published method.^[^ ^37^ ^]^ Briefly, liver tissues were homogenized in NP‐40 lysis buffer (10 mM Tris‐HCl, pH 7.4, 10 mM NaCl, 3 mM MgCl_2_, and 0.5% (vol/vol) NP‐40 in DEPC H_2_O) and centrifuged sequentially at 300 × *g* at 4 °C. The supernatant is the cytosolic extract. Nuclei pellets were washed once in 1 mL NP‐40 lysis buffer. Total RNA was extracted from nuclei and cytosol using the TriPure Isolation Reagent (Roche, Mannheim, Germany).

### Primary Hepatocyte Isolation

Primary hepatocytes were isolated from C57BL/6 wild‐type (WT) mice at postnatal days 20 and 60 by liver perfusion with type II collagenase (Worthington Biochem, Lakewood, NJ), following a previously published protocol.^[^ ^7^, ^8^ ^]^ For hepatocyte isolation from C57BL/6 WT mice at postnatal days 1, 5, and 10, mouse livers were harvested, minced into small pieces, and incubated at 37 °C for 10 min in Hanks’ Balanced Salt Solution (pH 7.8) supplemented with 1 mg mL^−1^ collagenase II (Roche Diagnostics).

### Nonhepatocyte (Mainly Liver Lymphocytes) Isolation

For the isolation of nonhepatocytes (primarily liver lymphocytes), mouse livers were pressed through a 70 µm sterile cell strainer using a 3 mL syringe plunger into a 6‐well plate. The first 5 mL of the resulting cell suspension was transferred into a 50 mL conical tube using a new cell strainer for filtration. To maximize cell recovery, an additional 5 mL of wash buffer was added to the same well and pressed through the same strainer. This second 5 mL suspension was also added to the same 50 mL conical tube. After discarding the liver cell strainer, the well and conical tube were washed twice with 2–5 mL of wash buffer per wash. The washes were added to the 50 mL conical tube, which now contained the final cell suspension. Red blood cells were removed using red cell lysis buffer (155 mM NH_4_Cl, 10 mM KHCO_3_, 0.1 mM EDTA, pH: 7.3). The remaining nonhepatocytes (primarily liver lymphocytes) were then processed for RNA isolation and immunoblotting.

### Real Time Quantitative PCR (RT‐qPCR)

RT‐qPCR was performed as shown previously.^[^ ^38^ ^]^ Briefly, total RNA was extracted using the TriPure Isolation Reagent (Roche, Mannheim, Germany), and first‐strand cDNA was synthesized using random primers and M‐MLV reverse transcriptase (Promega, Madison, WI). RT‐qPCR was performed using a Roche LightCycler 480 real‐time PCR system (Roche, Mannheim, Germany). The expression of individual genes was normalized to the expression of 36B4. Primers for real time RT‐qPCR were listed in Table S5 (Supporting Information).

### Immunoblotting

Liver tissues were homogenized in a RIPA buffer (50 mM Tris‐HCl, pH 7.5, 150 mM NaCl, 1% Triton X100, 0.1% SDS, 1% deoxycholate, 2 mM EDTA, 1 mM PMSF). These lysates were then immunoblotted using the indicated antibodies. Antibody dilutions were as follows: Tubulin (sc‐5286, Santa cruz), 1:5000; YTHDC1 (CST, 54737S) 1:2500; YTHDF2 (Proteintech, 24744‐1‐AP) 1:2500; YTHDF3 (Proteintech, 25537‐1‐AP) 1:2500; FOXA1 (Proteintech, 20411‐1‐AP) 1:1000; FOXA2 (Proteintech, 22474‐1‐AP) 1:2000; β‐Actin (Bimake, A5538) 1:5000.

### RNA Pull‐Down Assay

For the RNA pull‐down assay, biotinylated RNA probes targeting *Foxa1* and *Foxa2* were synthesized with the following sequences: *Foxa1*‐A (5′ to 3′): Biotin‐GGUUGGACGGCGCGUACGCCAUGGGACUCAUGCA, *Foxa1*‐m^6^A (5′ to 3′): Biotin‐GGUUGGACGGCGCGUACGCCAUGGG/m^6^A/CUCAUGCA, *Foxa2*‐A (5′ to 3′): Biotin‐GGGCCUCUGGUGGCAGGCCUGGGGACUCAGCGC, and *Foxa2*‐m^6^A (5′ to 3′): Biotin‐GGGCCUCUGGUGGCAGGCCUGGGG/m^6^A/CUCAGCGC. To begin the assay, 60 µL of streptavidin beads (SA053005, Smart‐Lifesciences) was washed once with RIPA buffer and then incubated with 300 µL of RIPA buffer, 3 µL of recombinant RNase inhibitor (2313Q, Takara), and 1 nmol of the biotinylated probe at 4 °C for 2 h with gentle rotation. Protein lysates were prepared by homogenizing 40 mg of either wild‐type mouse liver tissue or Ad‐FLAG‐YTHDC1 adenovirus‐mediated overexpression mouse liver tissue in 400 µL of RIPA buffer. Forty microliters of the probe‐bound beads were then added to the lysate and incubated at 4 °C for 1 h with rotation. After centrifuging the samples at 7000 rpm for 2 min, the supernatant was collected as the input to remove nonspecifically bound proteins. The probe‐bound beads were then washed once with RIPA buffer and incubated with 350 µL of the prepared lysate at 4 °C for 2 h with rotation to allow RNA–protein interactions. After incubation, the beads were washed three times with RIPA buffer to remove unbound material. Finally, protein complexes were analyzed using immunoblotting to assess the enrichment of specific protein–RNA interactions.

### RNA‐Sequencing

Liver tissues were collected from WT mice during postnatal development (day 1, day 10, day 20, and day 60 after birth), *Ythdc1* ^flox/flox^ and *Ythdc1*‐HKO mice at 9 weeks old. Four‐week‐old male *Ythdc1* ^flox/flox^ mice were injected with AAV8‐TBG‐βGal (2 × 10^11^ vp per mouse) via tail vein. Four‐week‐old male *Ythdc1‐*HKO mice were injected with equal amounts of AAV8–TBG–βGal, AAV8–TBG–FOXA1, or AAV8–TBG–FOXA2 via tail vein. Mice were sacrificed three weeks later. Total RNA was extracted using Tripure Isolation Reagent (Roche, Mannheim, Germany) from collected liver tissues. Three independent biological replicates for each group were used for RNA‐seq. RNA‐seq was performed by deep sequencing using Illumina Novaseq 6000 platform or Illumina novaseq x plus. Paired‐end clean reads were aligned to the mouse reference genome (GRCm38/mm10, GRCm38.p6, and GRCm39, respectively) with Hisat2 (v2.0.5 or V2.2.1), and the aligned reads were used to quantify mRNA expression by using featureCounts (v1.5.0‐p3 or v2.0.6). RNA‐seq analysis were performed as described previously.^[^ ^38^, ^39^, ^40^ ^]^ RNA‐seq data that support the findings of this study have been deposited in GEO under accession code GSE227284, GSE227172, and GSE297453.

### ATAC‐Sequencing

For Assay for Transposase‐Accessible Chromatin with high‐throughput sequencing (ATAC‐seq), liver samples were collected from *Ythdc1*‐HKO and *Ythdc1* ^flox/flox^ mice (*n* = 4 for each group). Nuclei were extracted and resuspended in the Tn5 transposase reaction mix. The transposition reaction was incubated at 37 °C for 30 min. Following transposition, equimolar amounts of adapter1 and adapter2 were added, and PCR was performed to amplify the library. Post‐PCR, libraries were purified using AMPure beads and assessed for quality with Qubit. The index‐coded samples were clustered on a cBot Cluster Generation System using the TruSeq PE Cluster Kit v3‐cBot‐HS (Illumina) as per the manufacturer's instructions. The libraries were then sequenced on an Illumina NovaSeq 6000 platform, generating 150 bp paired‐end reads. Raw data (raw reads) of fastq format were firstly processed using fastp (v 0.20.0). In this step, clean data (clean reads) were obtained by removing reads containing adapter, reads containing ploy‐N and low‐quality reads (reads with a mass value of less than 15 bases more than 40% of the number of bases of the reads and pruned reads shorter than 18 bp) from raw data. At the same time, Q20, Q30, and GC content of the clean data were calculated. All the downstream analyses were based on the clean data. Reference genome and gene annotation files were downloaded from genome website directly. Index of the mouse reference genome (grcm38_p6) was built using BWA (v 0.7.12) and clean reads were aligned to the reference genome using BWA mem. Reads that were derived from mitochondrion DNA and chloroplast DNA were discarded. These reads were then filtered for high quality (MAPQ ≥ 13), reads that were not properly paired and with PCR duplicates were also removed. Only uniquely mapped (MAPQ ≥ 13) and deduplicated reads were used for further analysis. All peak calling was performed with MACS2 (v 2.1.0) using “macs2 ‐q 0.05 –call‐summits –nomodel –shift ‐100 –extsize 200 –keep‐dup all”. By default, peaks with *q*‐value threshold of 0.05 were used for all data sets. Peaks were adjusted to the same size (500 bp) centered peak summits and motif discoveries of these loci sequences were performed using findMotifsGenome.pl program in HOMER (v 4.9.1) checksoftware with “‐len 8,10,12,14 ‐gc ‐size given ‐p 2 ‐S 25 ‐homer2 ‐dumpFasta”. The position of peak summit around transcript start sites of genes can predict the interaction sites between protein and gene. ChIPseeker was used to retrieve the nearest genes around the peak and annotate genomic region of the peak. Peak‐related genes can be confirmed by ChIPseeker, and then GO enrichment analysis was performed to identify the function enrichment results. GO enrichment analysis was implemented by the GOseq R package, in which gene length bias was corrected. GO terms with corrected *P*‐value less than 0.05 were considered significantly enriched by peak‐related genes. KEGG is a database resource for understanding high level functions and utilities of the biological system, such as the cell, the organism and the ecosystem, from molecular‐level information, especially large‐scale molecular datasets generated by genome sequencing and other high‐throughput experimental technologies (http://www.genome.jp/kegg/). KOBAS software was used to test the statistical enrichment of peak related genes in KEGG pathways. ATAC‐seq data that support the findings of this study have been deposited in GEO under accession code GSE273788.

### RNA m^6^A Immunoprecipitation (MeRIP)

MeRIP was performed as shown previously.^[^ ^40^, ^41^ ^]^ Briefly, total RNA (300 µg) was extracted from the livers of *Ythdc1*‐HKO and *Ythdc1* ^flox/flox^ mice, followed by fragmentation to obtain RNA fragments of suitable size. The RNA concentration was adjusted to 1 µg µL^−1^, and 33.3 µL of 10× Fragmentation Buffer (100 mM Tris‐HCl, 100 mM ZnCl₂) was added. The RNA was incubated at 94 °C for 3 min, and the fragmentation reaction was halted by the addition of 37 µL of 0.5 m EDTA. The mixture was then gently vortexed, briefly centrifuged, and placed on ice. For input preparation, 5 µL of the fragmented RNA was immediately used for reverse transcription (RT). The remaining 90 µL of fragmented RNA was incubated with 2.5 µL of RNase inhibitor, 100 µL of 5 × IP buffer, and 12.5 µL of m^6^A‐specific antibody (202003, Synaptic System) at 4 °C for 2 h with rotation to allow antibody binding. In parallel, 60 µL of Protein A beads per sample was blocked by resuspending in blocking solution and rotating for 1 h at 4 °C, followed by washing three times with 1 × IP buffer. The antibody‐incubated RNA was then transferred to the blocked beads and incubated at 4 °C for 2 h with rotation for immunoprecipitation. After incubation, the beads were washed three times with 1 × IP buffer (1 mL per wash) to remove nonspecifically bound material. Finally, RNA was eluted from the beads and subjected to RT‐qPCR analysis to assess the m^6^A modification levels.

### RNA Immunoprecipitation Sequencing (RIP‐Seq)

Primary hepatocytes were isolated from C57BL/6 WT mice via liver perfusion with type II collagenase (Worthington Biochem, Lakewood, NJ) and cultured at 37 °C with 5% CO_2_ in RPMI 1640 medium supplemented with 3% FBS. These hepatocytes were infected overnight with Ad‐FLAG‐YTHDC1 adenoviruses. Cells were harvested in a lysis buffer (100 mM KCl, 5 mM MgCl_2_, 10 mM HEPES (pH 7.0), 0.5% NP40, 1 mM DTT, 100 units mL^−1^ RNaseOut) prepared in DEPC H_2_O. Ten percent of the cell lysate was used as input, and the remaining lysate was immunoprecipitated with 100 µL anti‐FLAG M2 magnetic beads (M8823, Millipore) at 4 °C for 2 h. The immunoprecipitated samples were washed four times with lysis buffer. Total RNA was extracted from both the immunoprecipitated and input samples using the TriPure Isolation Reagent (Roche, Mannheim, Germany). mRNA was purified using the Hieff NGS mRNA Isolation Master Kit (12603, Yeasen Biotechnology). The fragment distribution and concentration of RNAs (after immunoprecipitation) were detected using an Agilent 2100 bioanalyzer (Agilent) and simpliNano spectrophotometer (GE Healthcare). Then, immunoprecipitated RNA or Input was used for library construction with NEB NextR Ultra RNA Library Prep Kit (New England Biolabs). Library quality was assessed on the Agilent Bioanalyzer 2100 system. The libraries were sequenced on an Illumina Novaseq 6000 platform with a paired‐end read length of 150 bp according to the standard protocols. Raw data (raw reads) of fastq format were first processed using fastp software. In this step, clean data (clean reads) were obtained by removing reads containing adapter, reads containing ploy‐N and low‐quality reads from raw data. At the same time, Q20, Q30, and GC content of the clean data were calculated. All the downstream analyses were based on the clean data with high quality. Index of the reference genome (GRCm38/mm10) was built using BWA (v 0.7.12) and clean reads were aligned to the reference genome using BWA mem (v 0.7.12). After mapping reads to the reference genome, the MACS2 (version 2.1.0) peak calling software was used to identify regions of IP enrichment over background. A *q*‐value threshold of 0.05 was used for all data sets. After peak calling, the distribution of chromosome distribution, peak width, fold enrichment, significant level, and peak summit number per peak were all displayed. The interaction between intracellular RNA and protein binding were not random, while they show some specific sequence preference. Homer (v4.9.1) was used to detect the de novo sequence motif and the matched known motifs. Peak related genes can be confirmed by Peak Annotator, and then GO enrichment analysis was performed to identify the function enrichment results. GO enrichment analysis was implemented by the GOseq R package, in which gene length bias was corrected. GO terms with corrected *p*‐value less than 0.05 were considered significantly enriched by peak related genes. KEGG is a database resource for understanding high‐level functions and utilities of the biological system, such as the cell, the organism, and the ecosystem, from molecular‐level information, especially large‐scale molecular datasets generated by genome sequencing and other high throughput experimental technologies (http://www.genome.jp/kegg/). KOBAS (3.0) software was used to test the statistical enrichment of peak related genes in KEGG pathways. RIP‐seq data that support the findings of this study have been deposited in GEO under accession code GSE273790. 

### Statistical Analysis

Data were presented as means ± SEM. The Shapiro‐Wilk test was employed to assess the normality of the data. When all groups were normally distributed (*P* > 0.05), the parametric two‐tailed Student's *t*‐tests were used to detect the statistical differences between the two groups. The parametric one‐factor analysis of variance (ANOVA), and Tukey was used to detect the statistical differences among four groups. When at least one of the two groups were not normally distributed (*P* < 0.05), the nonparametric Mann–Whitney test was adopted to compare the statistical differences between the two groups. The nonparametric Kruskal–Wallis and Dunn's was adopted to compare the statistical differences among four groups. All statistical analyses were performed using GraphPad Prism version 9.5.1 (GraphPad Software Inc., San Diego, CA, USA). *P* < 0.05 was considered statistically significant. *, *P* < 0.05. **, *P* < 0.01. ***, *P* < 0.001. ****, *P* < 0.0001.

## Conflict of Interest

The authors declare no conflict of interest.

## Author Contributions

X.L., X.L., and C.L. contributed equally to this work. Z.L., K.D., Y.W., N.G., and L.X. researched data. Z.C. conceived and designed the project, researched data, and wrote manuscript.

## Supporting information



Supporting Information

Supporting Information

Supplemental Table 1

Supplemental Table 2

Supplemental Table 3

Supplemental Table 4

Supplemental Table 5

## Data Availability

The data that support the findings of this study are available from the corresponding author upon reasonable request.
