# Supplementary material for: YTHDC1 Is Essential for Postnatal Liver Development and Homeostasis
Source: Adv Sci (Weinh). 2025 Jun 19;12(35):e05725. doi: 10.1002/advs.202505725 (PMC12462980; doi:10.1002/advs.202505725)
Supplement: Supplementary file 1 — Supporting Information [file ADVS-12-e05725-s007.docx]

Supporting Information

YTHDC1 is essential for postnatal liver development and homeostasis

Xinzhi Li, Xueying Li, Chunhong Liu, Zhenzhi Li, Kaixin Ding, Yuxin Wang, Ning Gu, Liwei Xie, and Zheng Chen^*^

**Figure S1. Global gene expression profiles in the liver change during postnatal development.**

(a-d) RNA-seq analysis was performed on the mouse livers at postnatal day 1, 10, 20, and 60.

Differentially expressed genes (DEGs) were categorized into four sub-clusters (Sub-cluster 1, 2, 3, and 4). (a) Sub-cluster 1 included genes that are gradually upregulated during postnatal liver development. (b) Sub-cluster 2 included genes that gradually downregulated during postnatal liver development. (c) Sub-cluster 3 included genes that dramatically downregulated during postnatal liver development. (d) Sub-cluster 4 included genes that dramatically upregulated during postnatal liver development.


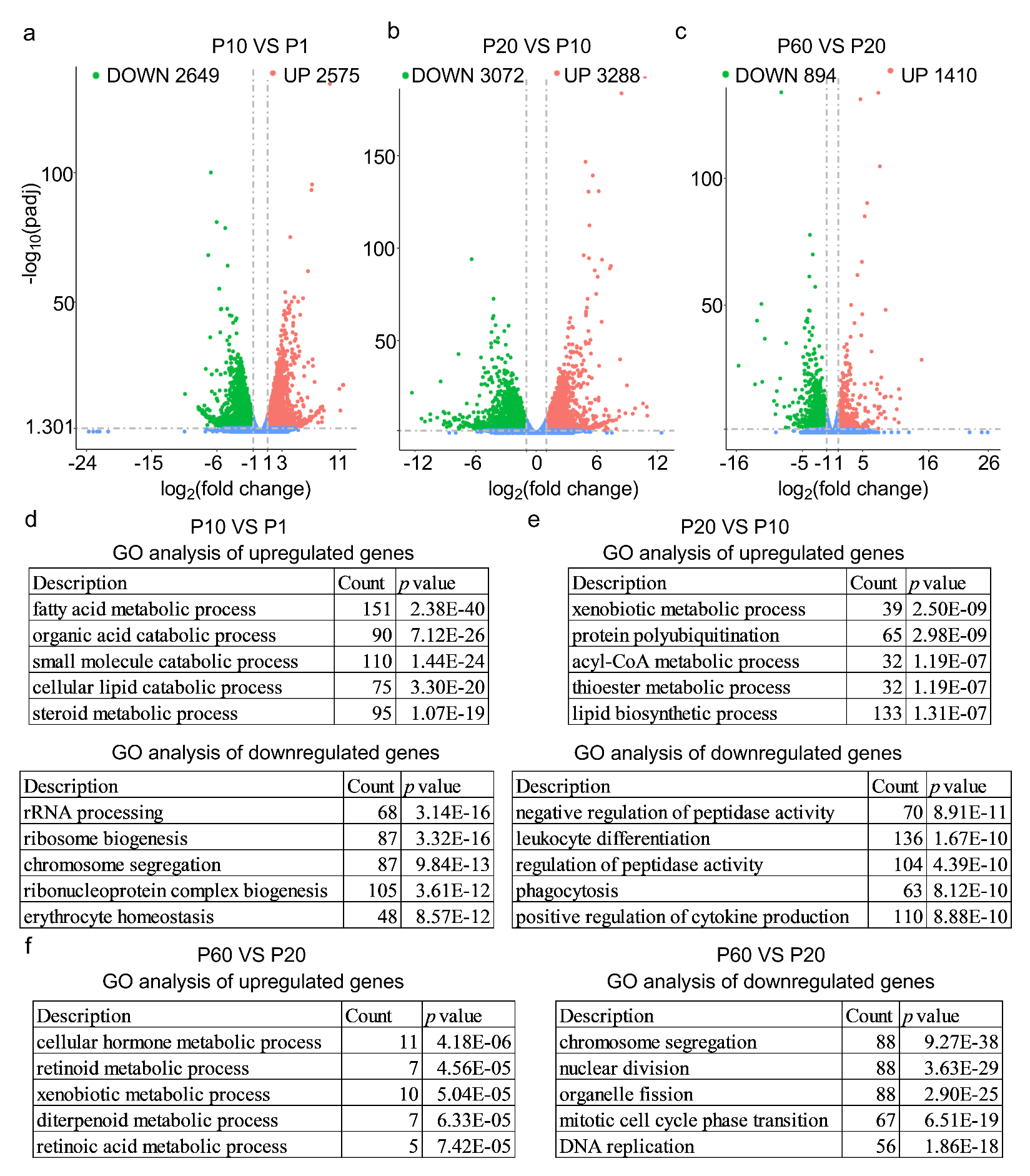


**Figure S2. Global gene expression profiles change in the liver during postnatal development.**

RNA-seq analysis was performed in the mouse livers at postnatal day 1, 10, 20 and 60.

(a) The differentially expressed genes (DEGs) between P10 and P1, including 2649 downregulated and 2575 upregulated genes, were displayed in a volcano plot (|log2foldchange|>1 and padj<0.05).

(b) The differentially expressed genes (DEGs) between P20 and P10, including 3072 downregulated and 3288 upregulated genes, were displayed in a volcano plot (|log2foldchange|>1 and padj<0.05).

(c) The differentially expressed genes (DEGs) between P60 and P20, including 894 downregulated and 1410 upregulated genes, were displayed in a volcano plot (|log2foldchange|>1 and padj<0.05).

(d) GO biological process terms enriched in upregulated and downregulated genes as shown in (a).

(e) GO biological process terms enriched in upregulated and downregulated genes as shown in (b).

(f) GO biological process terms enriched in upregulated and downregulated genes as shown in (c).


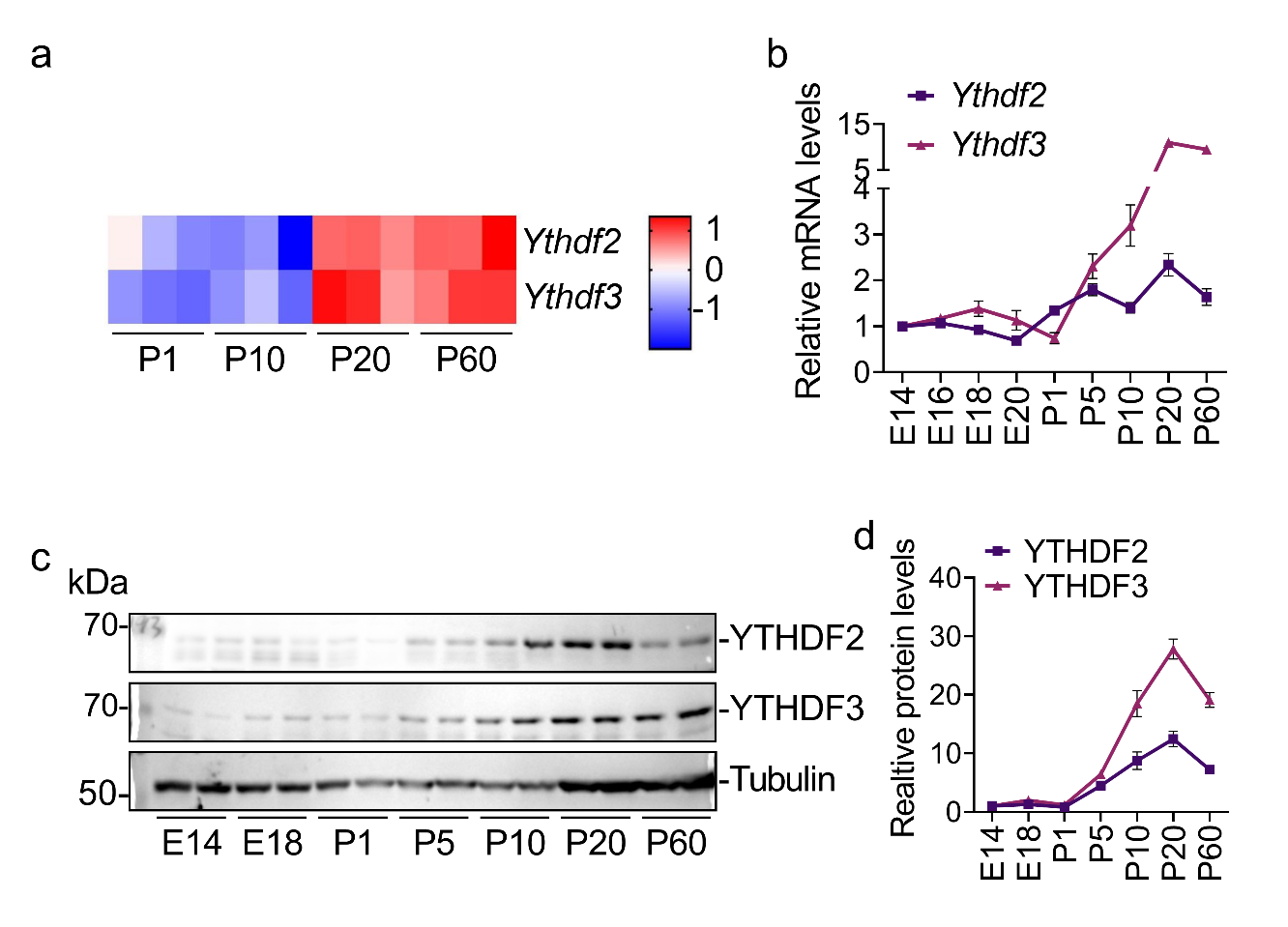


**Figure S3. *Ythdf2* and *Ythdf3* are upregulated during postnatal liver development.**

(a) A heatmap showed that *Ythdf2* and *Ythdf3* were significantly upregulated in the liver during postnatal development.

(b) *Ythdf2* and *Ythdf3* mRNA levels at embryonic day 14, 16, 18, 20, postnatal day 1, 5, 10, 20 and 60 were measured by RT-qPCR (n=7 per group).

(c-d) YTHDF2 and YTHDF3 protein levels at embryonic day 14, 18, postnatal day 1, 5, 10, 20 and 60 were measured by immunoblotting and quantified using ImageJ (n=2 for representative, n=8 for quantification).

n was the number of biologically independent mice. Data represent the mean ± SEM.


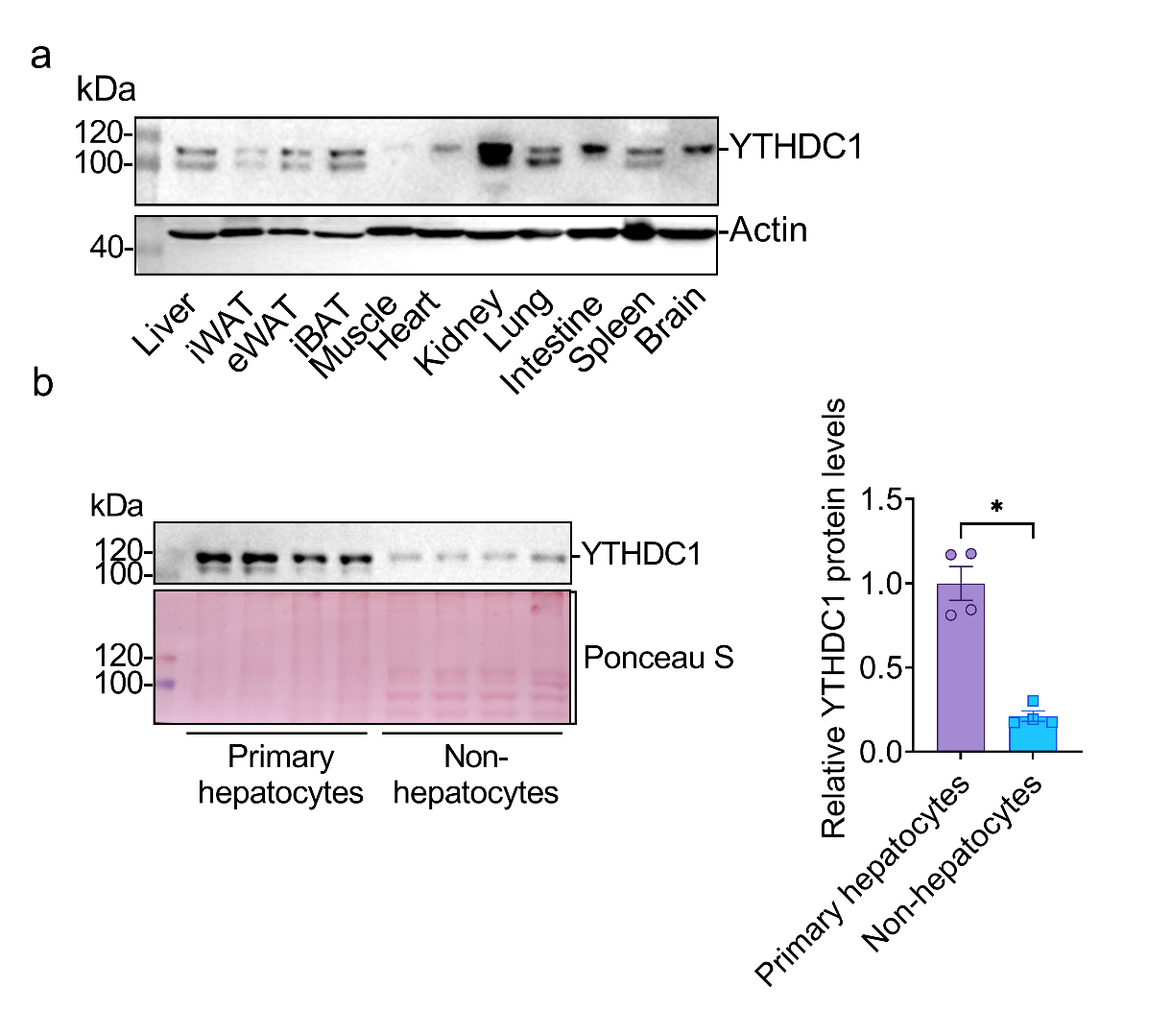


**Figure S4. YTHDC1 is highly expressed in the liver and hepatocytes.**

(a) YTHDC1 and Actin protein levels were measured in the liver, iWAT, eWAT, iBAT, skeletal muscle, heart, kidney, lung, small intestine, spleen, and brain of WT mice by immunoblotting.

(b) Primary hepatocytes and non-hepatocytes were isolated from WT mice. YTHDC1 protein levels were measured by immunoblotting and quantified using Image J (n=4 per group). n was the number of biologically independent cell samples. Data represent the mean ± SEM. The Shapiro-Wilk test was employed to assess the normality of the data. One of the two groups was not normally distributed (*P* < 0.05), the non-parametric Mann-Whitney test was adopted to compare the statistical differences between the two groups. *, *P*< 0.05.


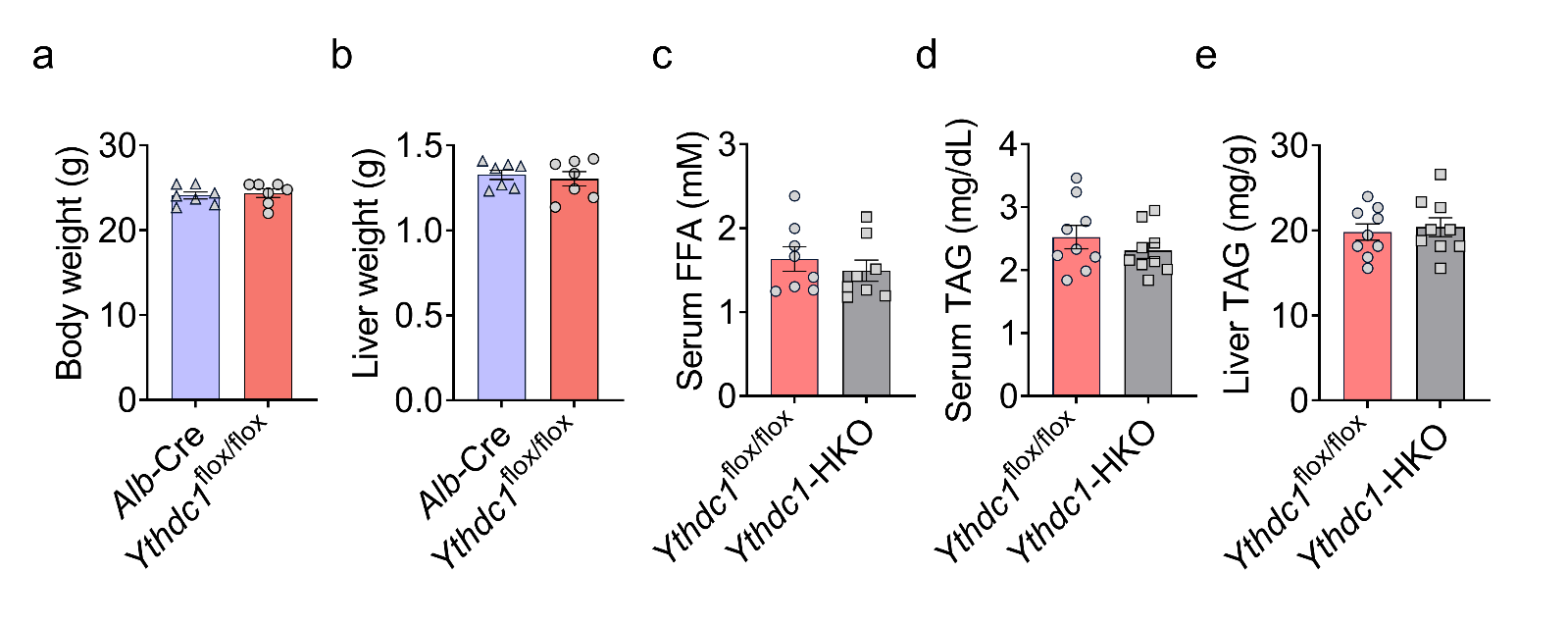


**Figure S5. Phenotypes of *Alb*-Cre, *Ythdc1*^flox/flox^ and *Ythdc1*-HKO mice.**

(a-b) Body and liver weights were measured in 8-week-old *Ythdc1*^flox/flox^ and *Alb*-Cre mice fed a normal chow diet (n=7 per group).

(c-d) Serum FFA and TAG levels were measured in 9-week-old *Ythdc1*^flox/flox^ and *Ythdc1*-HKO mice (n=9 per group).

(e) Liver TAG levels were measured in 9-week-old *Ythdc1*^flox/flox^ and *Ythdc1*-HKO mice (n=9 per group).

n was the number of biologically independent mice. Data represent the mean ± SEM.


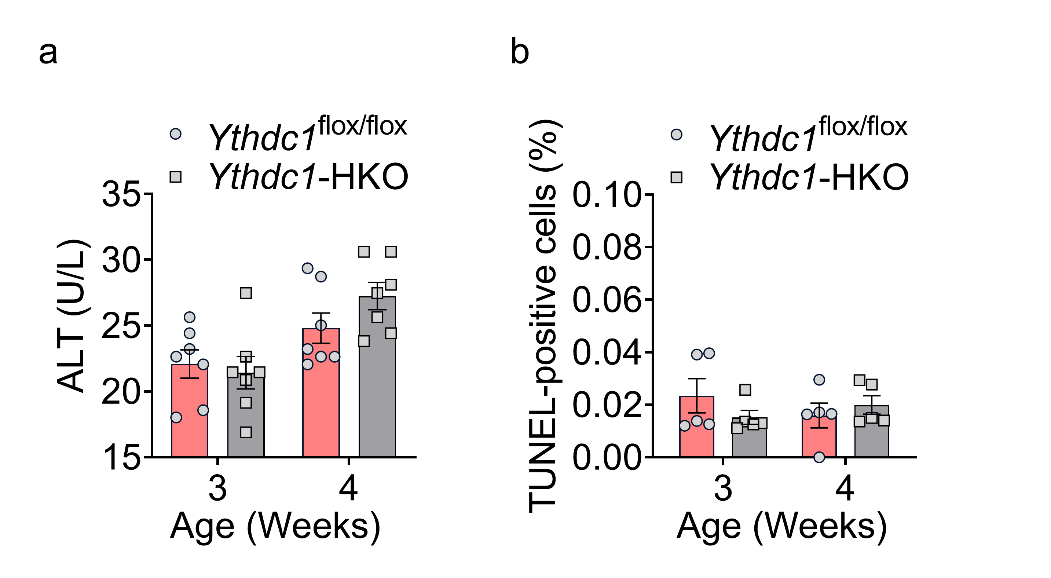


**Figure S6. *Ythdc1*-HKO mice show normal serum ALT levels and similar numbers of TUNEL-positive cells at 3 and 4 weeks old.**

(a) Serum ALT activity was measured in 3- and 4-week-old *Ythdc1*^flox/flox^ and *Ythdc1*-HKO mice (n=7 per group).

(b) TUNEL-positive cells in 3- and 4-week-old *Ythdc1*^flox/flox^ and *Ythdc1*-HKO liver sections were quantified (n=5 per group).

n was the number of biologically independent mice. Data represent the mean ± SEM.


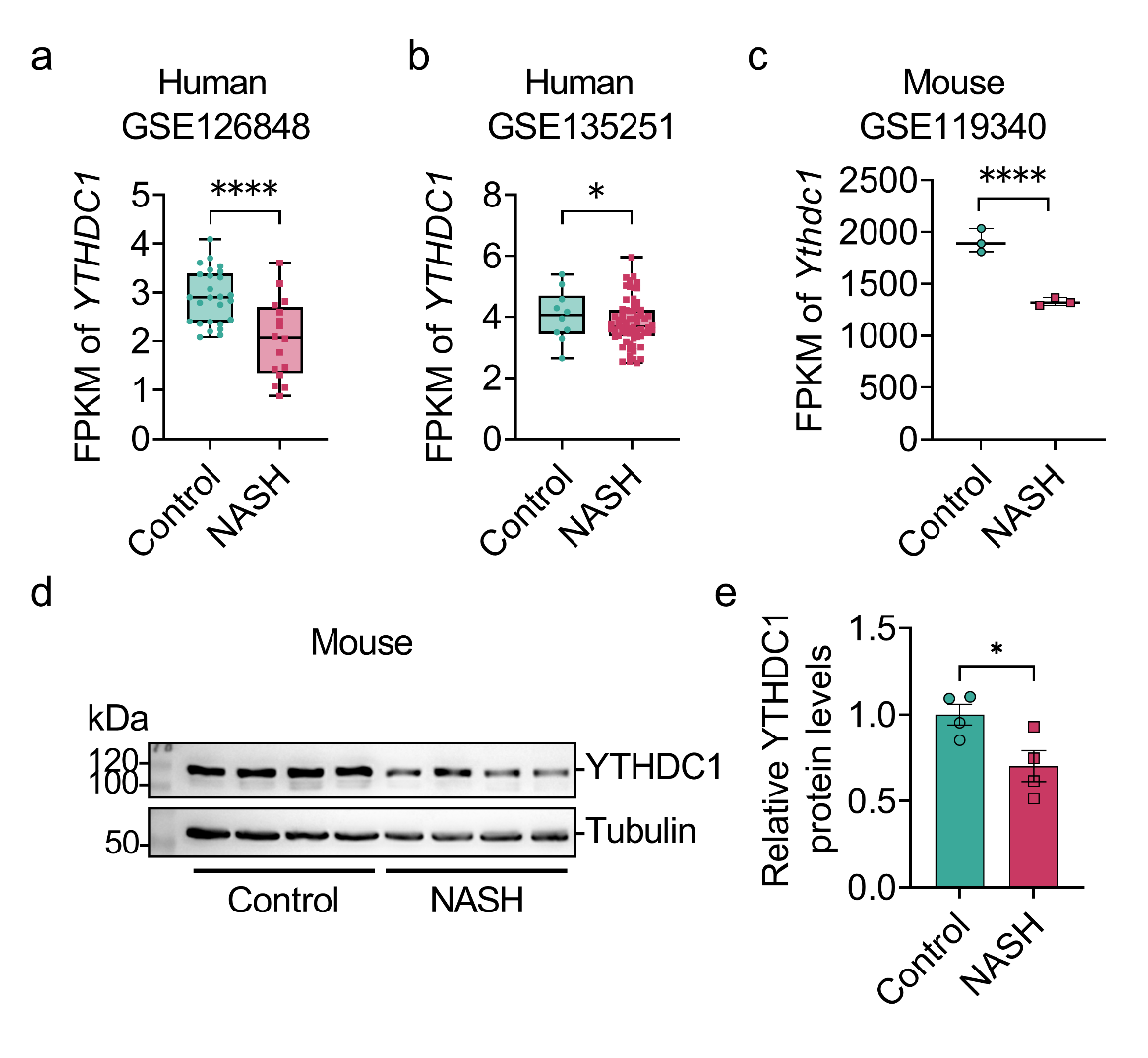


**Figure S7. *YTHDC1* is downregulated in both human samples and mouse models of NASH.**

(a) FPKM of *YTHDC1* in Control and NASH samples (GSE126848) (n=26 for Control; n=16 for NASH). (b) FPKM of *YTHDC1* in control and NASH samples (GSE135251) (n=10 for Control; n=68 for NASH). (b) FPKM of *Ythdc1* in control and NASH samples (GSE119340) (n=3 per group). n was the number of biologically independent samples. Data represent the mean ± SEM. *, *P*< 0.05. ****, *P*< 0.0001.


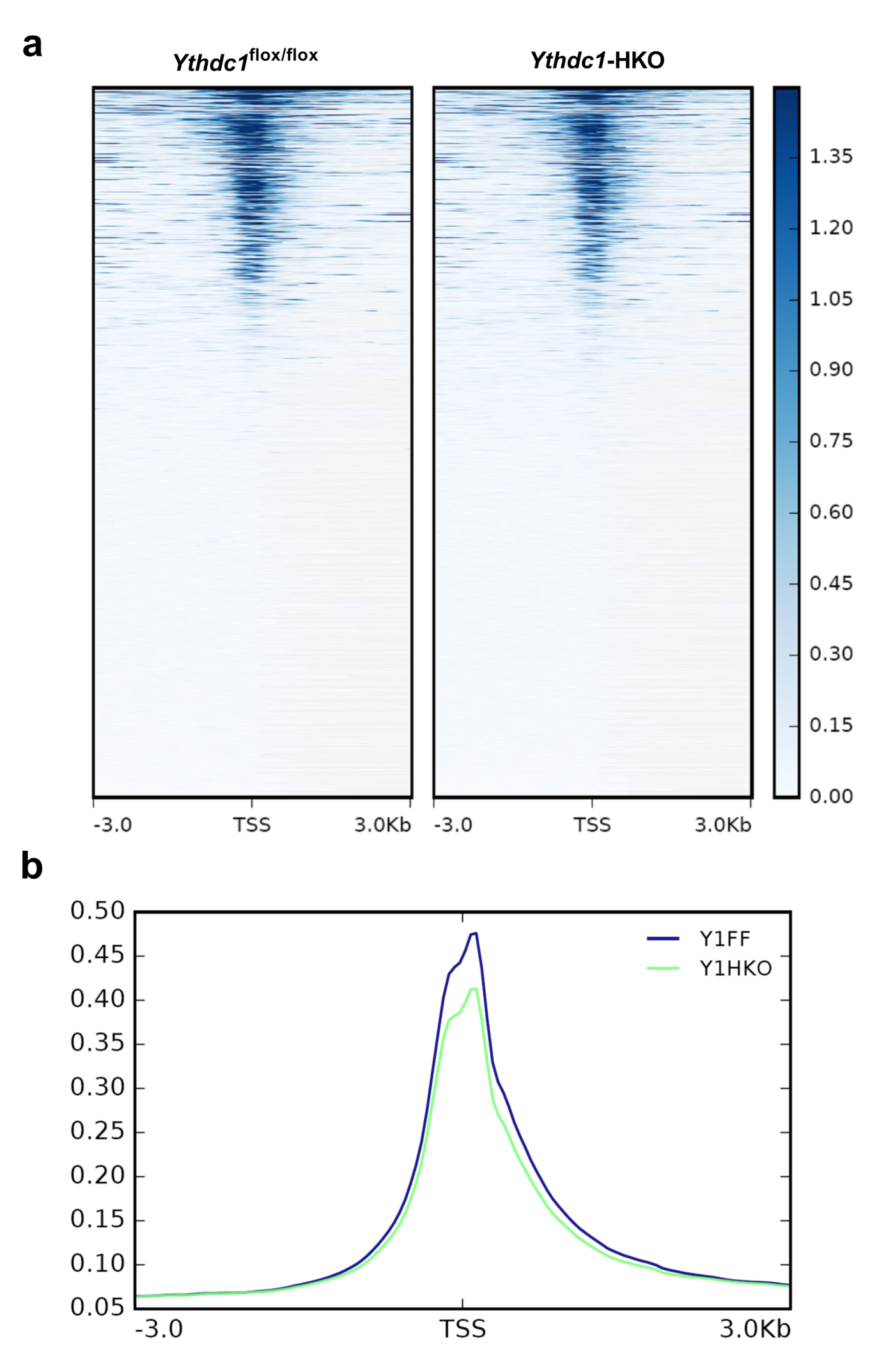


**Figure S8. Enrichment of open chromatin in the gene promoters and the ATAC-seq peaks near the TSS in the livers of *Ythdc1*^flox/flox^ and *Ythdc1*-HKO mice.**

(a) Reads heatmap around TSS. (b) Reads profile around TSS.


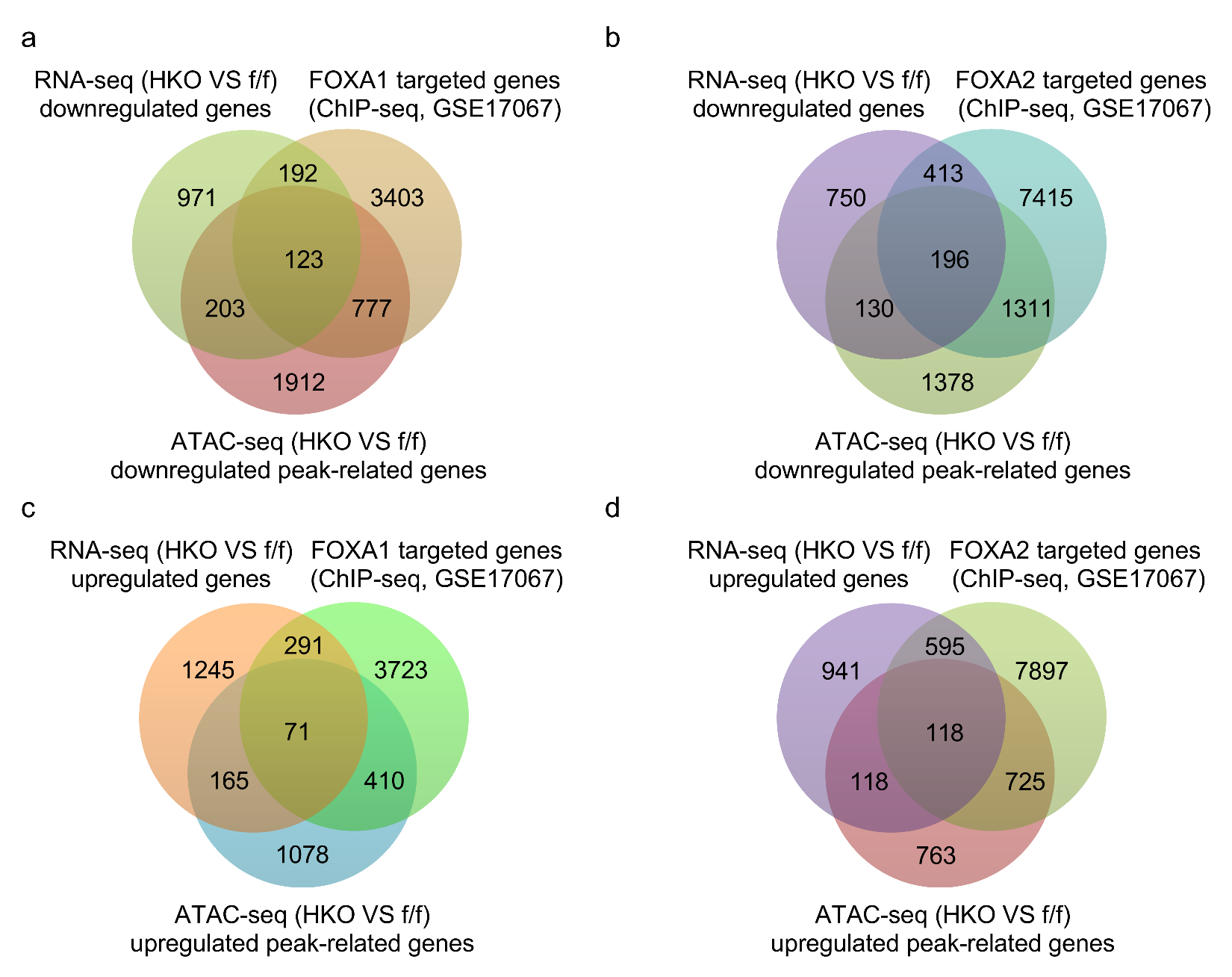


**Figure S9. Combined analysis of RNA-seq data, ATAC-seq data, FOXA1 targeted genes, and FOXA2 targeted genes.**

(a) Venn diagram analysis of RNA-seq (HKO VS f/f) downregulated genes, ATAC-seq (HKO VS f/f) downregulated peak-related genes, and FOXA1 targeted genes (ChIP-seq, GSE17067). (b) Venn diagram analysis of RNA-seq (HKO VS f/f) downregulated genes, ATAC-seq (HKO VS f/f) downregulated peak-related genes, and FOXA2 targeted genes (ChIP-seq, GSE17067).

(c) Venn diagram analysis of RNA-seq (HKO VS f/f) upregulated genes, ATAC-seq (HKO VS f/f) upregulated peak-related genes, and FOXA1 targeted genes (ChIP-seq, GSE17067).

(d) Venn diagram analysis of RNA-seq (HKO VS f/f) upregulated genes, ATAC-seq (HKO VS f/f) upregulated peak-related genes, and FOXA2 targeted genes (ChIP-seq, GSE17067).


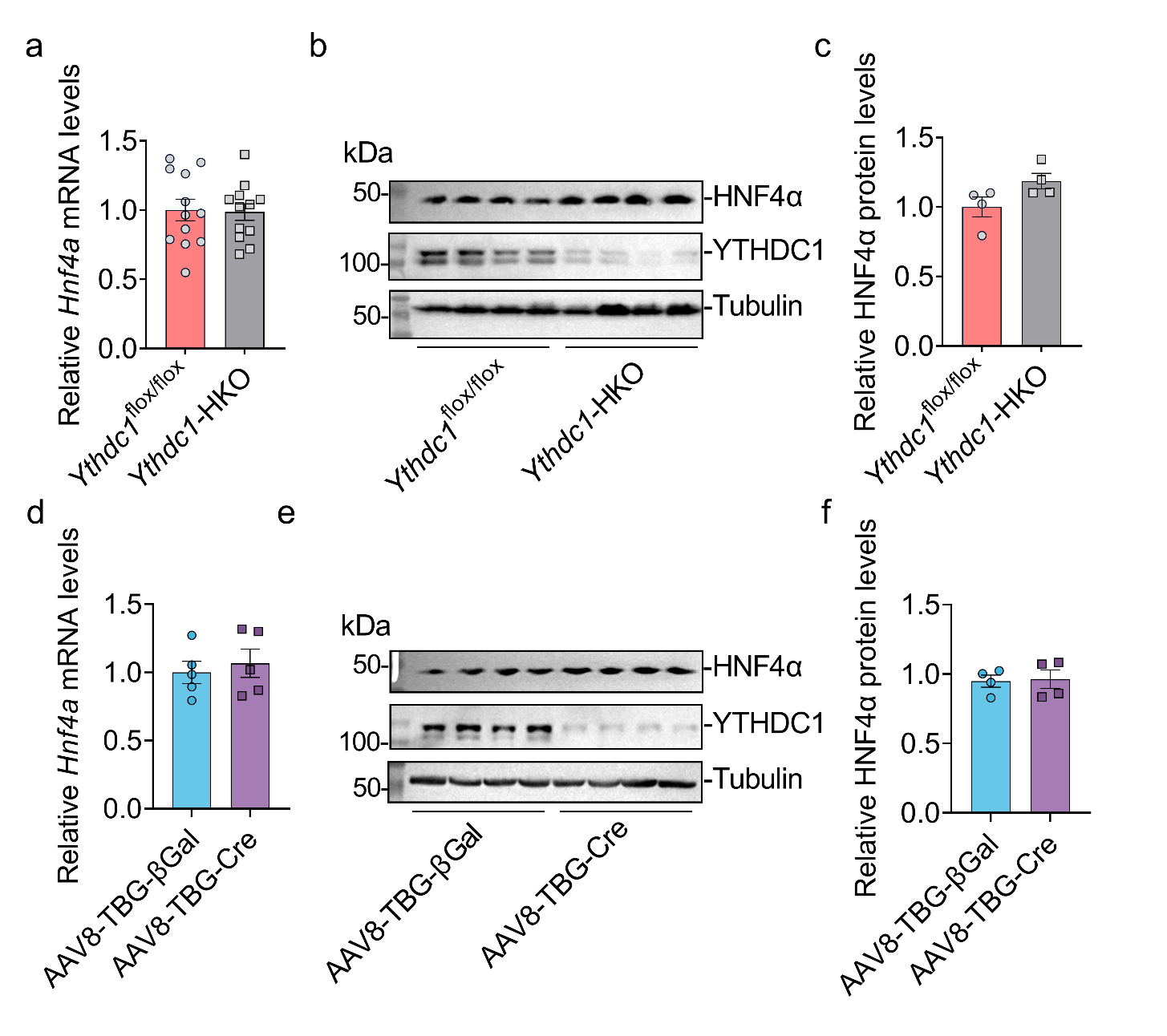


**Figure S10. The expression of HNF4α is not altered in either *Ythdc1*-HKO or *Ythdc1*-adultHKO livers.**

(a) Relative *Hnf4a* mRNA levels in *Ythdc1*^flox/flox^ and *Ythdc1*-HKO livers were measured by RT-qPCR (n=12 per group).

(b-c) HNF4α, YTHDC1, and Tubulin protein levels in *Ythdc1*^flox/flox^ and *Ythdc1*-HKO livers were measured by immunoblotting and quantified using Image J (n=4 per group).

(d-f) *Ythdc1*^flox/flox^ mice at 8 weeks old were injected with AAV8-TBG-Cre or AAV8-TBG-βGal via tail vein for 16 days. (d) Relative *Hnf4a* mRNA levels were measured by RT-qPCR (n=5 per group). (e-f) HNF4α, YTHDC1, and Tubulin protein levels in the livers of these mice were measured by immunoblotting and quantified using Image J (n=4 per group). n was the number of biologically independent mice. Data represent the mean ± SEM.


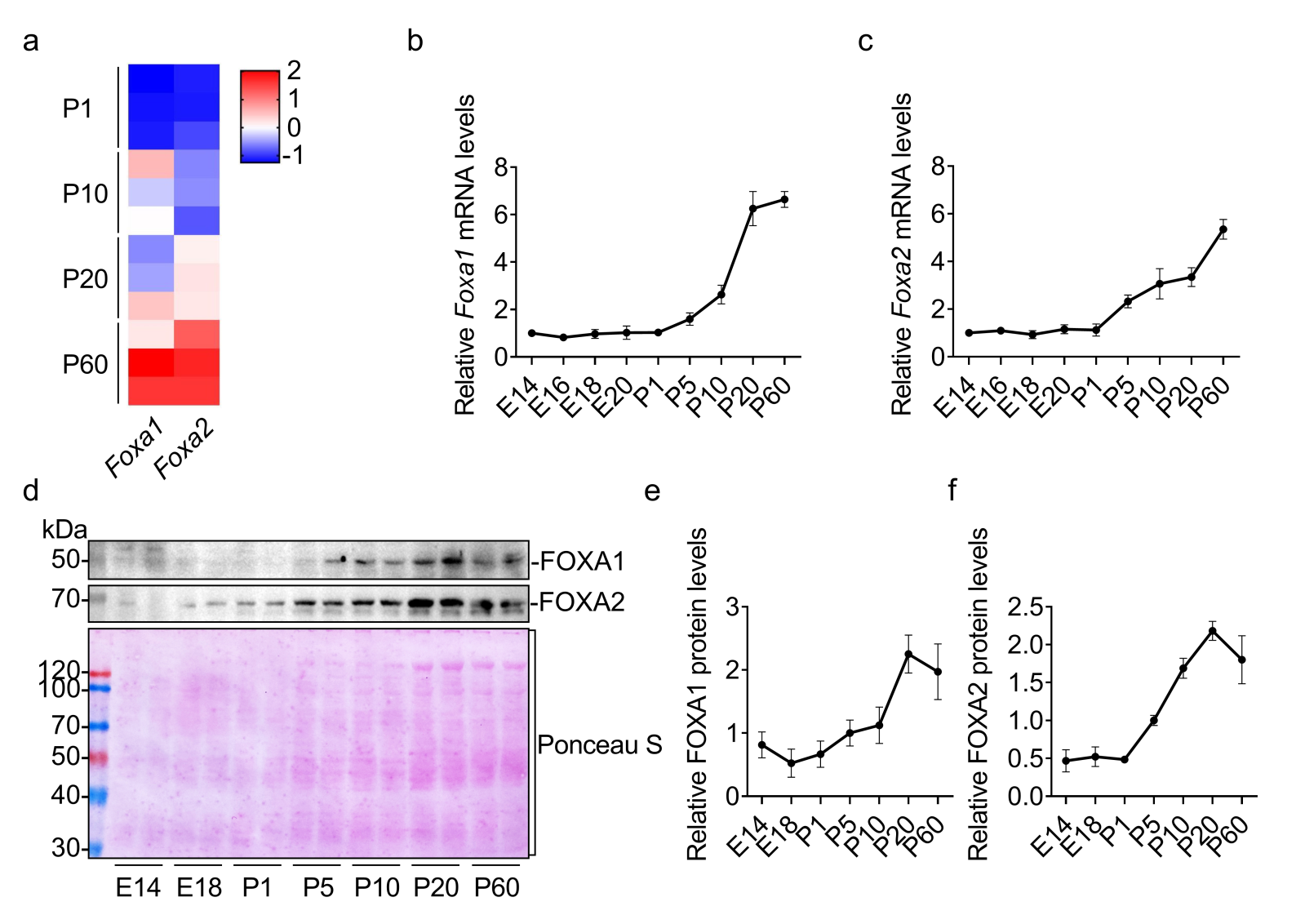


**Figure S11. FOXA1 and FOXA2 were upregulated in the livers during postnatal development.**

(a) A heatmap showed that *Foxa1* and *Foxa2* were significantly upregulated in the liver during postnatal development.

(b-c) *Foxa1* and *Foxa2* mRNA levels at embryonic day 14, 16, 18, 20, postnatal day 1, 5, 10, 20 and 60 were measured by RT-qPCR (n=7 per group).

(d-f) FOXA1 and FOXA2 protein levels at embryonic day 14, 18, postnatal day 1, 5, 10, 20 and 60 were measured by immunoblotting and quantified using ImageJ (n=2 for representative, n=8 for quantification).

n was the number of biologically independent mice. Data represent the mean ± SEM.


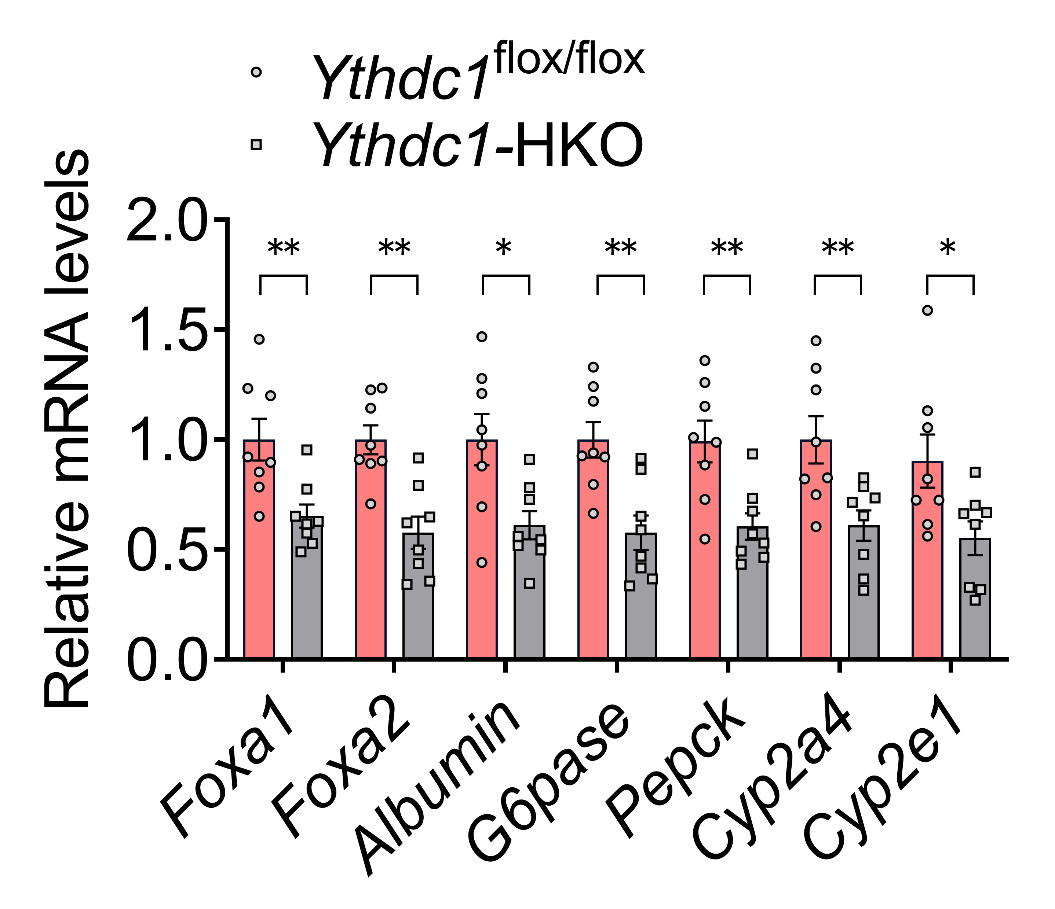


**Figure S12. The expression of transcription factors (FOXA1, FOXA2), the liver marker Albumin, gluconeogenesis-related genes (e.g., *G6pase*, *Pepck*), and cytochrome P450 enzymes (e.g., Cyp2a4, Cyp2e1) in** **the livers of 3-week-old *Ythdc1*^flox/flox^ and** ***Ythdc1*-HKO mice.**

Relative mRNA levels of *Foxa1*, *Foxa2*, *Albumin*, *G6pase*, *Pepck*, *Cyp2a4*, and *Cyp2e1* in the livers of 3-week-old *Ythdc1*^flox/flox^ and *Ythdc1*-HKO mice were measured by RT-qPCR (n=8 per group).

n was the number of biologically independent mice. Data represent the mean ± SEM. The Shapiro-Wilk test was employed to assess the normality of the data. When both groups were normally distributed (*P* > 0.05), the parametric two-tailed Student’s t tests were used to detect the statistical differences between the two groups. When at least one of the two groups were not normally distributed (*P* < 0.05), the non-parametric Mann-Whitney test was adopted to compare the statistical differences between the two groups.*, *P*< 0.05. **, *P*< 0.01.


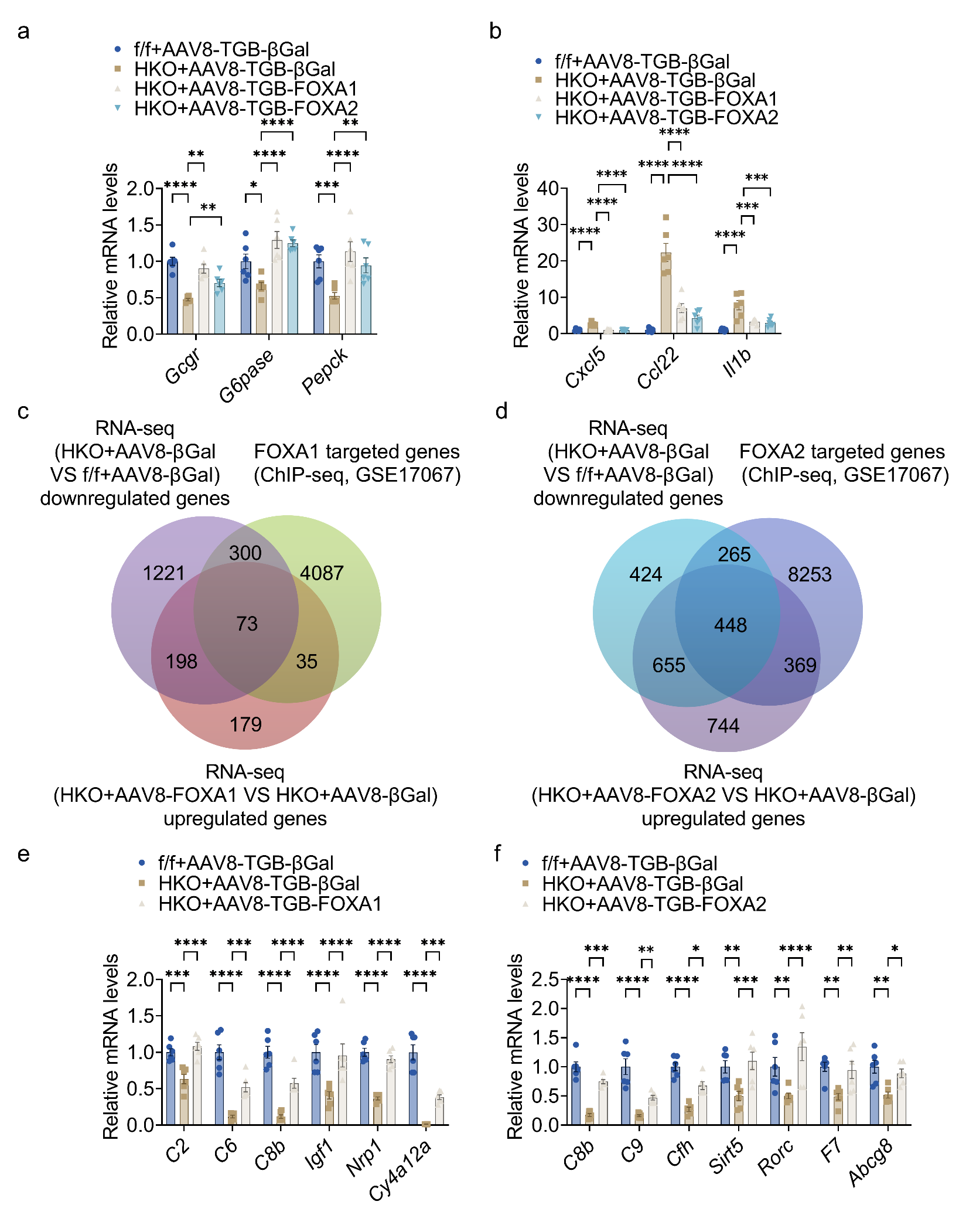


**Figure S13. Hepatocyte-specific overexpression of FOXA1 or FOXA2 rescues impaired liver function in *Ythdc1*-HKO mice.**

Four-week-old male *Ythdc1*^flox/flox^ mice were injected with AAV8-TBG-βGal (2×10^11 vp/mouse) via tail vein. Four-week-old male *Ythdc1-*HKO mice were injected with equal amounts of AAV8-TBG-βGal, AAV8-TBG-FOXA1, or AAV8-TBG-FOXA2 via tail vein. Phenotypes were measured, and mice were sacrificed three weeks later. RNA-seq and RT-qPCR analysis were performed (n=3 per group for RNA-seq; n=6 per group for RT-qPCR).

(a) *Gcgr*, *G6pase*, and *Pepck* mRNA levels were measured by RT-qPCR (n=6 per group).

(b) *Cxcl5*, *Ccl22*, and *Il1b* mRNA levels were measured by RT-qPCR (n=6 per group).

(c) Venn diagram analysis of RNA-seq (HKO+AAV8-TBG-βGal VS f/f+AAV8-TBG-βGal) downregulated genes, RNA-seq (HKO+AAV8-TBG-FOXA1 VS HKO+AAV8-TBG-βGal) upregulated peak-related genes, and FOXA1 targeted genes (ChIP-seq, GSE17067).

(d) Venn diagram analysis of RNA-seq (HKO+AAV8-TBG-βGal VS f/f+AAV8-TBG-βGal) downregulated genes, RNA-seq (HKO+AAV8-TBG-FOXA2 VS HKO+AAV8-TBG-βGal) upregulated peak-related genes, and FOXA2 targeted genes (ChIP-seq, GSE17067).

(e) *C2*, *C6*, *C8b*, *Igf1*, *Nrp1*, and *Cy4a12a* mRNA levels were measured by RT-qPCR (n=6 per group).

(f) *C8b*, *C9*, *Cfh*, *Sirt5*, *Rorc*, *F7*, and *Abcg8* mRNA levels were measured by RT-qPCR (n=6 per group).

n was the number of biologically independent mice. Data represent the mean ± SEM. The Shapiro-Wilk test was employed to assess the normality of the data. When all groups were normally distributed (*P* > 0.05), the parametric one-factor analysis of variance (ANOVA), and Tukey was used to detect the statistical differences. When at least one of the two groups were not normally distributed (*P* < 0.05), the non-parametric Kruskal-Wallis and Dunn's was adopted to compare the statistical differences. *, *P*< 0.05. **, *P*< 0.01. ***. *P*< 0.001. ****, *P*< 0.0001.


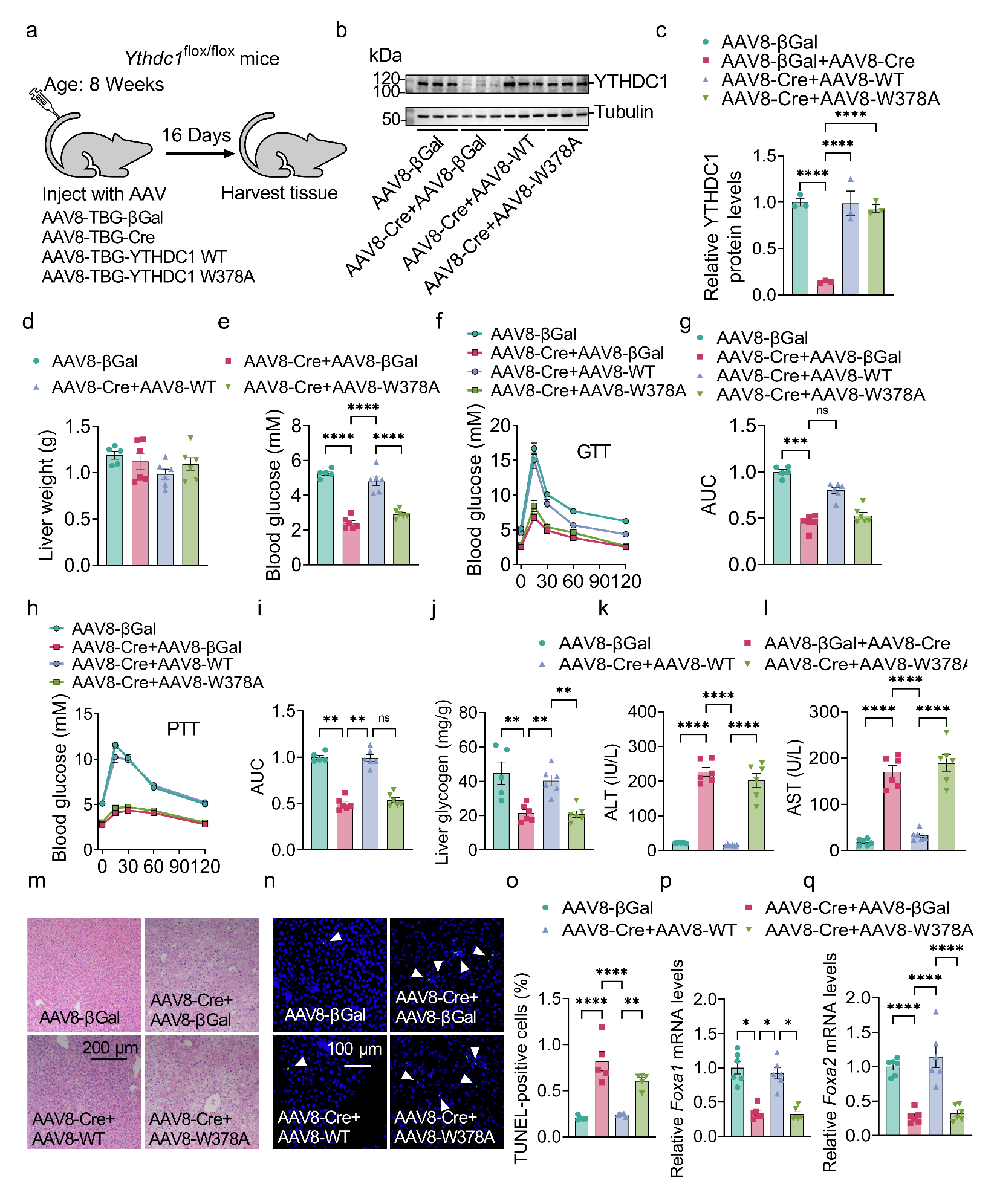


##### **Figure S14. YTHDC1, but not YTHDC1 W378A mutation, fully rescues the impaired liver function in *Ythdc1*-adultHKO mice**

(a) Eight-week-old male *Ythdc1*^flox/flox^ mice were injected with AAV8-TBG-βGal, AAV8-TBG-Cre＆AAV8-TBG-βGal, AAV8-TBG-Cre＆AAV8-TBG-YTHDC1 or AAV8-TBG-Cre＆AAV8-TBG- YTHDC1(W378A) viruses via tail vein. Phenotypes were measured, and mice were sacrificed sixteen days later.

(b-c) YTHDC1 and Tubulin protein levels were measured by immunoblotting and quantified using Image J (n=3 per group).

(d) Liver weights were measured (n=5-6 per group).

(e) Blood glucose levels were determined (n=5-6 per group).

(f-g) GTT and relative AUC were determined (n=5-6 per group).

(h-i) PTT and relative AUC were measured (n=5-6 per group).

(j) Liver glycogen levels were determined (n=5-6 per group).

(k-l) Serum ALT and AST activities were determined (n=5-6 per group).

(m) H&E staining was performed. Representative images were shown.

(n-o) TUNEL-positive cells were quantified (n=5 per group).

(p-q) *Foxa1* and *Foxa2* mRNA levels were measured by RT-qPCR (n=6 per group).

n was the number of biologically independent mice. Data represent the mean ± SEM. The Shapiro-Wilk test was employed to assess the normality of the data. When all groups were normally distributed (*P* > 0.05), the parametric one-factor analysis of variance (ANOVA), and Tukey was used to detect the statistical differences. When at least one of the two groups were not normally distributed (*P* < 0.05), the non-parametric Kruskal-Wallis and Dunn's was adopted to compare the statistical differences. *, *P*< 0.05. **, *P*< 0.01. ***. *P*< 0.001. ****, *P*< 0.0001.


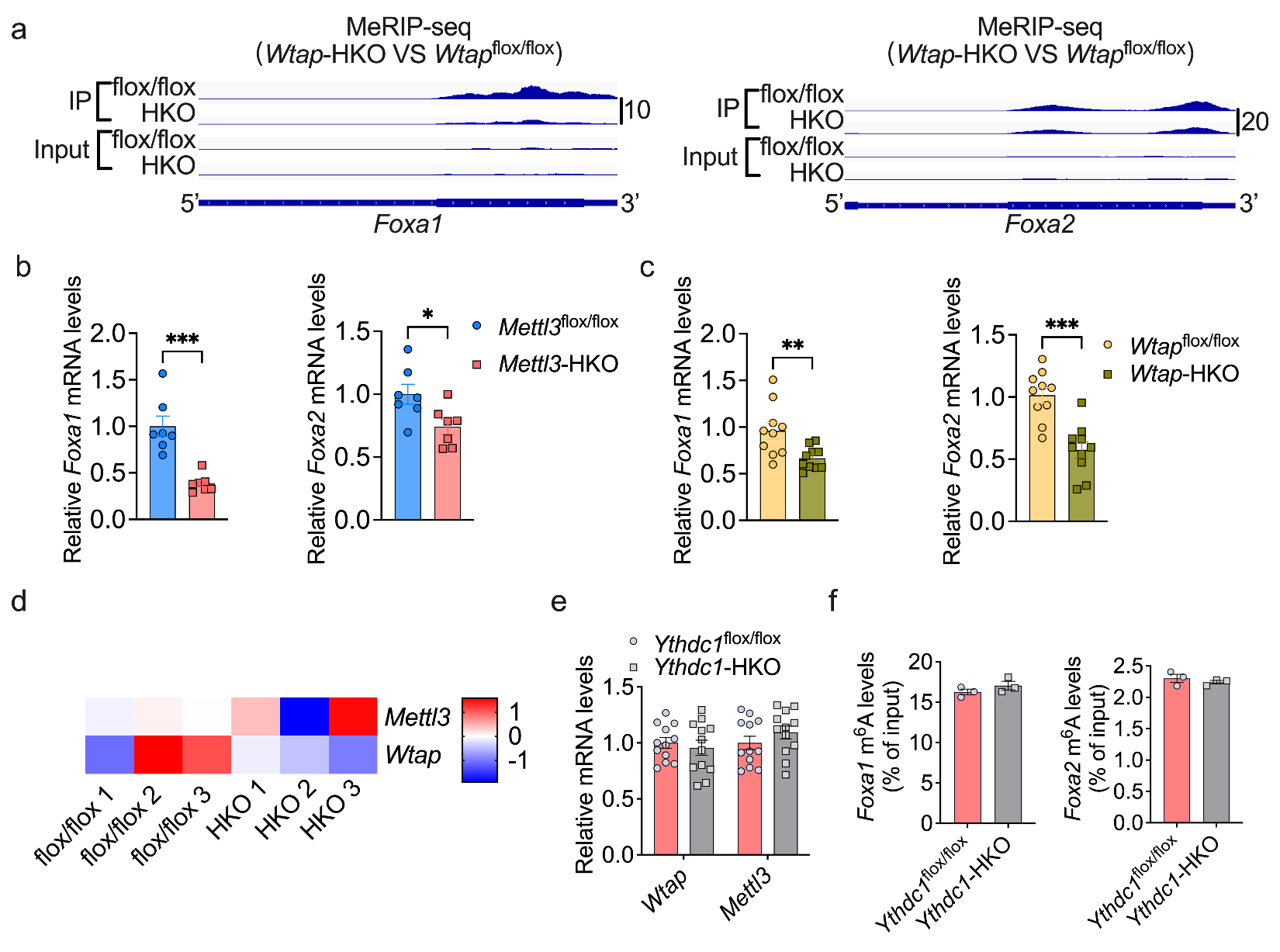


**Figure S15. The m^6^A modification of *Foxa1* and *Foxa2* is mediated by the m^6^A writer proteins METTL3 and WTAP.**

(a) The read density from MeRIP-seq experiment comparing *Wtap*-HKO to *Wtap*^flox/flox^ shows the m^6^A peaks identified in *Foxa1* and *Foxa2* transcripts.

(b) Relative *Foxa1* and *Foxa2* mRNA levels were measured by RT-qPCR in *Mettl3*-HKO and *Mettl3*^flox/flox^ livers (n=7 per group).

(c) Relative *Foxa1* and *Foxa2* mRNA levels were measured by RT-qPCR in *Wtap*-HKO and *Wtap*^flox/flox^ livers (n=10 per group).

(d) A heatmap showed the relative *Mettl3* and *Wtap* mRNA levels in *Ythdc1*^flox/flox^ and *Ythdc1*-HKO livers (n=3 per group).

(e) Relative *Mettl3* and *Wtap* mRNA levels were measured by RT-qPCR in *Ythdc1*^flox/flox^ and *Ythdc1*-HKO livers (n=12 per group).

(f) The m^6^A levels of *Foxa1* and *Foxa2* in *Ythdc1*^flox/flox^ and *Ythdc1*-HKO livers were measured by MeRIP-RT-qPCR (n=3 per group).

Data represent the mean ± SEM. n was the number of biologically independent mice. The Shapiro-Wilk test was employed to assess the normality of the data. When both groups were normally distributed (*P* > 0.05), the parametric two-tailed Student’s t tests were used to detect the statistical differences between the two groups. When at least one of the two groups were not normally distributed (*P* < 0.05), the non-parametric Mann-Whitney test was adopted to compare the statistical differences between the two groups. *, *P*< 0.05. **, *P*< 0.01. ***. *P*< 0.001.


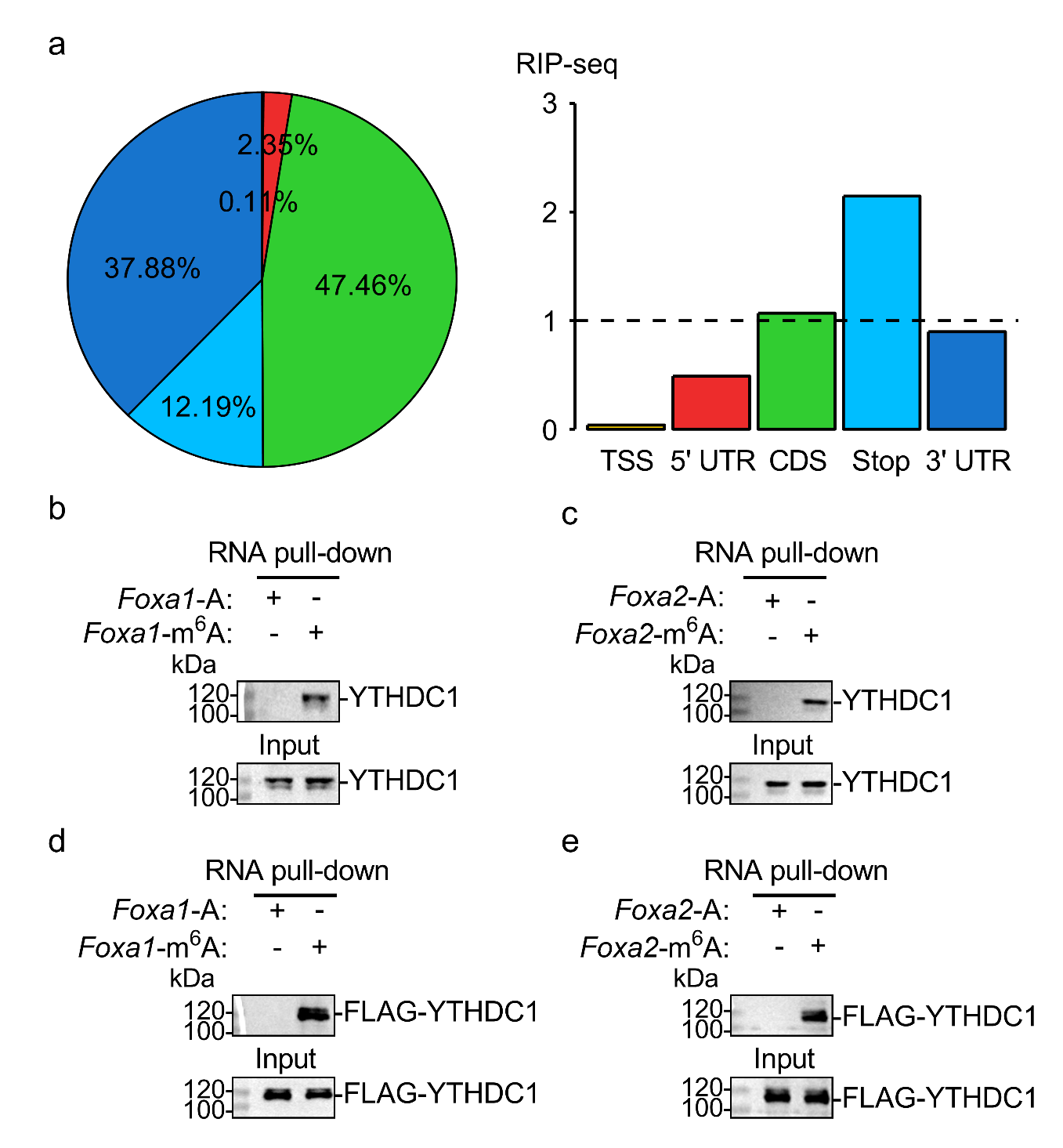


**Figure S16. YTHDC1 binds to the m^6^A modified *Foxa1* and *Foxa2* transcripts.**

(a) Primary hepatocytes were infected overnight with Ad-FLAG-YTHDC1 adenovirus. The enrichment of RIP-seq peaks in transcription start site (TSS), 5´-UTR, CDS, stop codon and 3´-UTR was analyzed.

(b-c) RNA pull-down experiments were performed to detect the binding of endogenous YTHDC1 to the m^6^A-modified probes of *Foxa1*/*Foxa2*. The m^6^A-unmodified probes served as negative controls.

(d-e) RNA pull-down experiments were performed to detect the binding of exogenous YTHDC1 to the m^6^A-modified probes of *Foxa1*/*Foxa2*. The m^6^A-unmodified probes served as negative controls. The cell culture experiments were repeated independently three times, yielding similar results each time.


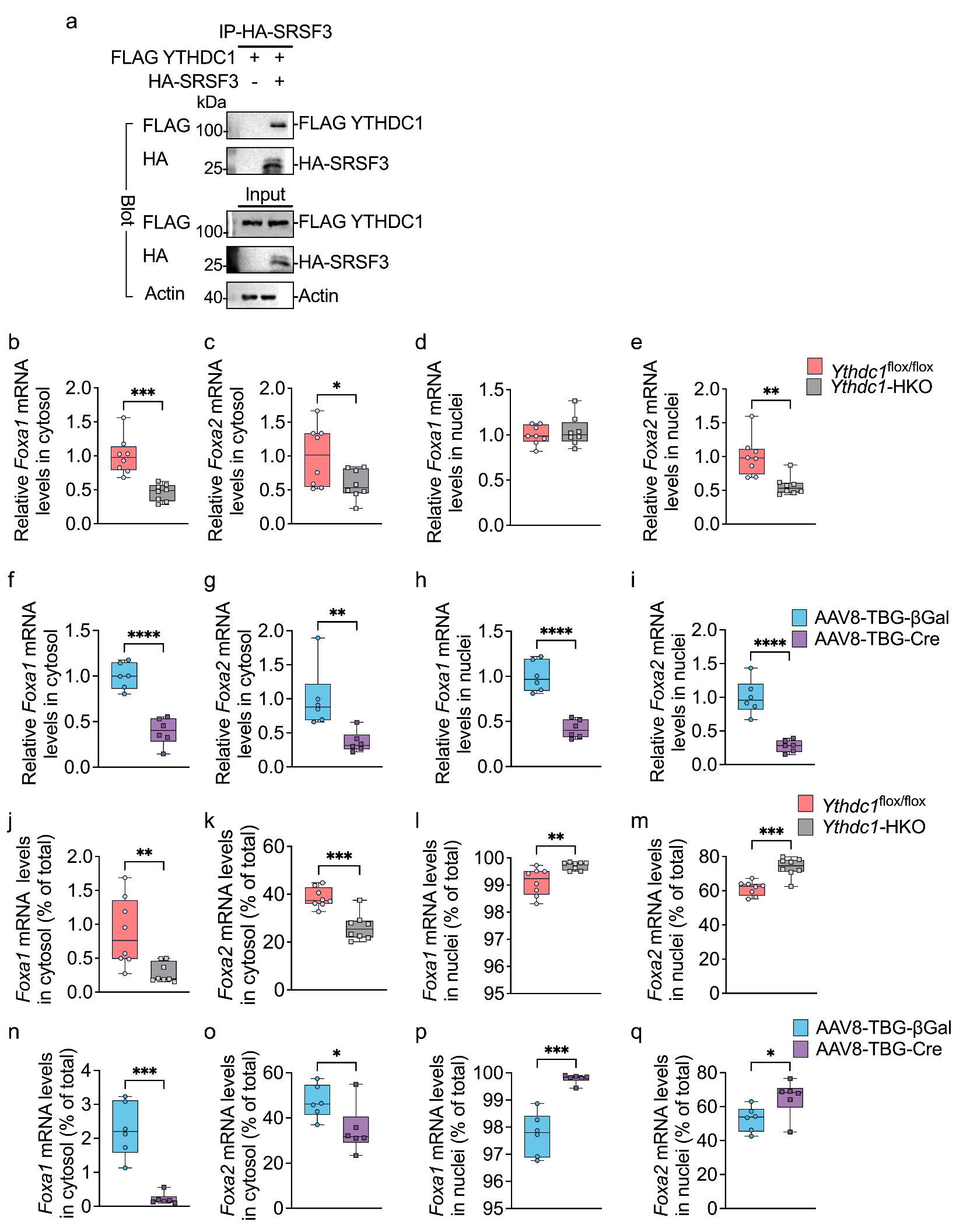


**Figure S17. YTHDC1 regulates mRNA transport from the nucleus to the cytosol.**

(a) FLAG-YTHDC1 expression vector was co-transfected with or without HA-SRSF3 expression vector in HEK293T cells. These lysates were immunoprecipitated with HA beads and then immublotted with anti-FLAG antibody. This cell culture experiment was repeated independently three times, yielding similar results each time.

(b-e) Cytosolic and nuclear *Foxa1* and *Foxa2* mRNA levels in the livers of 9-week-old *Ythdc1*^flox/flox^ and *Ythdc1*-HKO mice were measured by RT-qPCR (n=8 per group).

(f-i) *Ythdc1*^flox/flox^ mice at 8 weeks old were injected with AAV8-TBG-Cre or AAV8-TBG-βGal via tail vein. Mice at 16 days after AAV injection were sacrificed and liver tissues were collected. Cytosolic and nuclear *Foxa1* and *Foxa2* mRNA levels were measured by RT-qPCR (n=6 per group).

(j-m) Based on the data in b-e, the proportion of cytosolic and nuclear *Foxa1* and *Foxa2* mRNAs were calculated (n=8 per group).

(n-q) Based on the data in f-i, the proportion of cytosolic and nuclear *Foxa1* and *Foxa2* mRNAs were calculated (n=6 per group).

Data represent the mean ± SEM. n was the number of biologically independent mice. The Shapiro-Wilk test was employed to assess the normality of the data. When both groups were normally distributed (*P* > 0.05), the parametric two-tailed Student’s t tests were used to detect the statistical differences between the two groups. When at least one of the two groups were not normally distributed (*P* < 0.05), the non-parametric Mann-Whitney test was adopted to compare the statistical differences between the two groups. *, *P*< 0.05. **, *P*< 0.01. ***. *P*< 0.001.


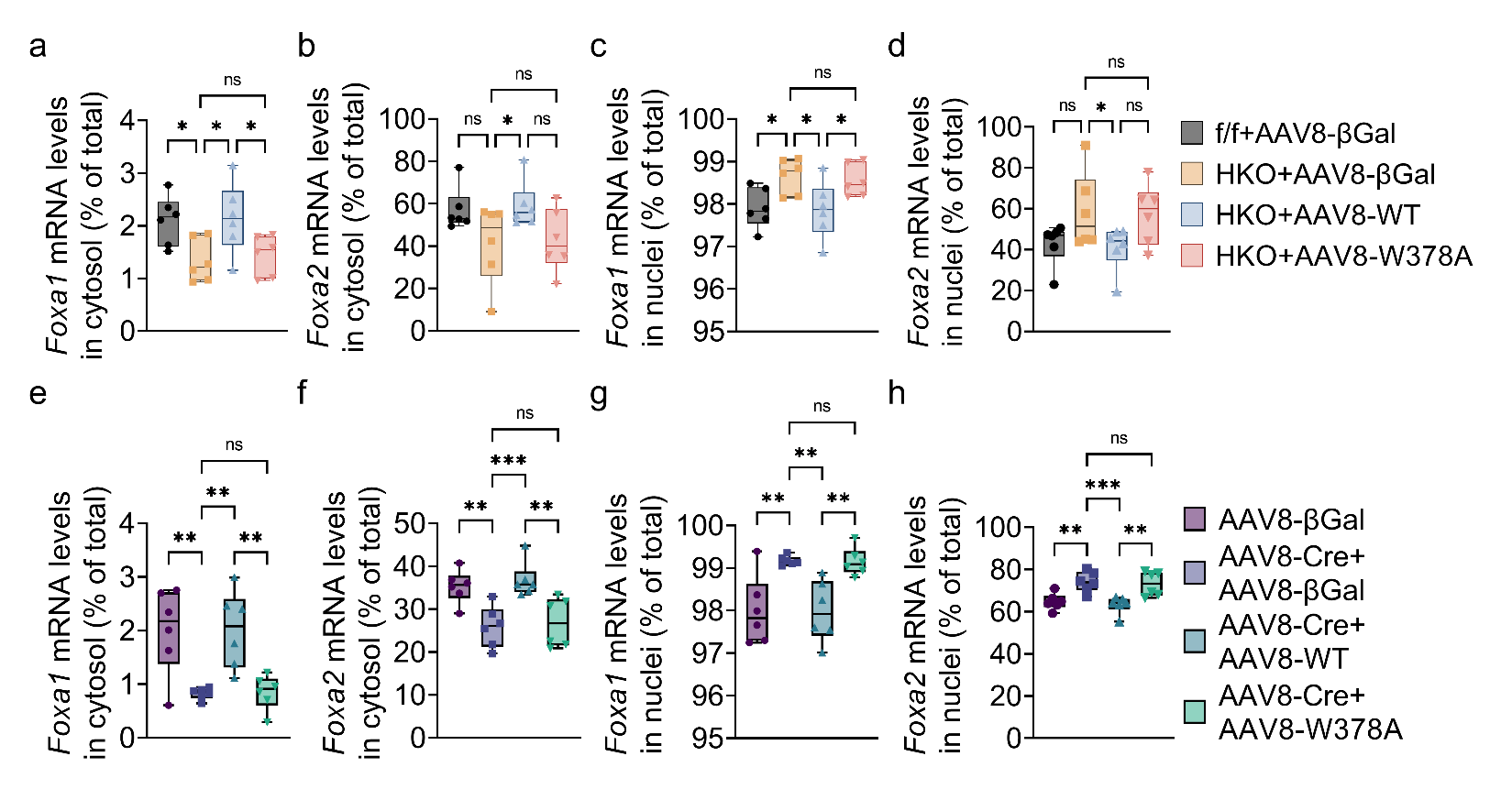


**Figure S18. YTHDC1 regulates mRNA transport from the nucleus to the cytosol through its m^6^A recognition.**

(a-d) Four-week-old male *Ythdc1*^flox/flox^ mice were injected with AAV8-TBG-βGal (2×10^11 vp/mouse) via tail vein. Four-week-old male *Ythdc1-*HKO mice were injected with equal amounts of AAV8-TBG-βGal, AAV8-TBG-YTHDC1 (WT), or AAV8-TBG-YTHDC1(W378A) via tail vein. Mice were sacrificed three weeks later, and liver tissues were collected. The proportion of cytosolic and nuclear *Foxa1* and *Foxa2* mRNAs were calculated based on the data in Figure 9j-m (n=6 per group).

(e-h) Adult-onset hepatocyte-specific *Ythdc1* knockout (*Ythdc1*-adultHKO) mice were generated by tail-vein injection of purified AAV8-TBG-Cre virus into adult *Ythdc1*^flox/flox^ mice. Re-expressing YTHDC1 or YTHDC1 W378A was achieved by tail-vein injection of purified AAV8-TBG-YTHDC1＆AAV8-TBG-Cre or AAV8-TBG-YTHDC1(E378A)＆AAV8-TBG-Cre virus into adult *Ythdc1*^flox/flox^ mice. Adult *Ythdc1*^flox/flox^ mice injected equal amounts of AAV8-TBG-βGal virus served as the control. Sixteen days later, mice were sacrificed, and liver tissues were collected. The proportion of cytosolic and nuclear *Foxa1* and *Foxa2* mRNAs were calculated based on the data in Figure 9n-q (n=6 per group). Data represent the mean ± SEM. n was the number of biologically independent mice. The Shapiro-Wilk test was employed to assess the normality of the data. When all groups were normally distributed (*P* > 0.05), the parametric one-factor analysis of variance (ANOVA), and Tukey was used to detect the statistical differences. When at least one of the two groups were not normally distributed (*P* < 0.05), the non-parametric Kruskal-Wallis and Dunn's was adopted to compare the statistical differences. *, *P*< 0.05. **, *P*< 0.01. ***. *P*< 0.001.
