# Supplementary material for: YTHDC1 Is Essential for Postnatal Liver Development and Homeostasis
Source: Adv Sci (Weinh). 2025 Jun 19;12(35):e05725. doi: 10.1002/advs.202505725 (PMC12462980; doi:10.1002/advs.202505725)

ADVANCED SCIENCE
Open Access

Supporting Information

for *Adv. Sci.*, DOI 10.1002/advs.202505725

YTHDC1 Is Essential for Postnatal Liver Development and Homeostasis

*Xinzhi Li, Xueying Li, Chunhong Liu, Zhenzhi Li, Kaixin Ding, Yuxin Wang, Ning Gu, Liwei Xie and Zheng Chen**

Figure 1g

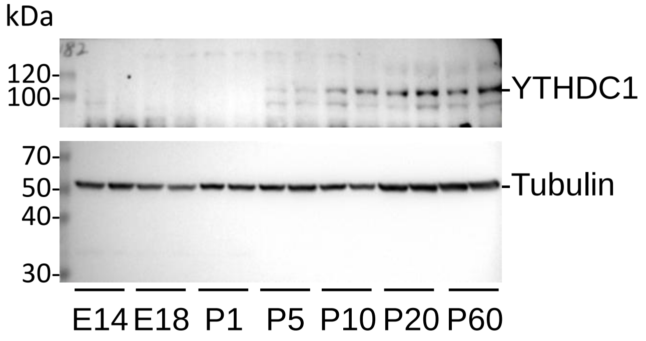

Figure 1k

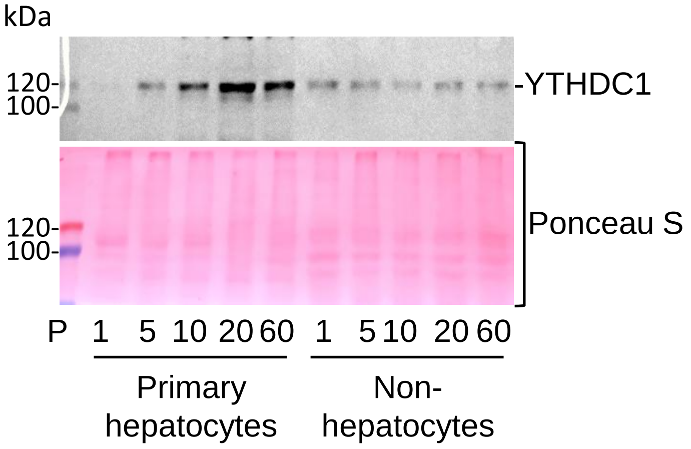

Figure 2a

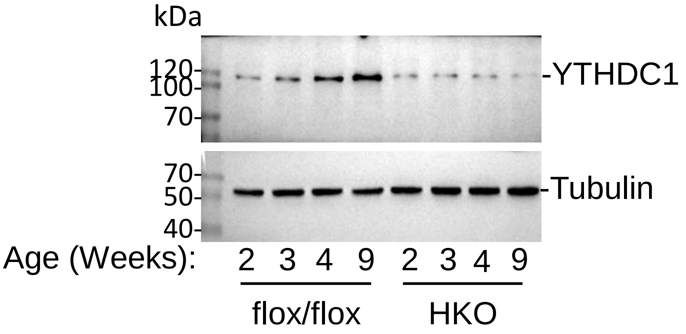

Figure 2b up

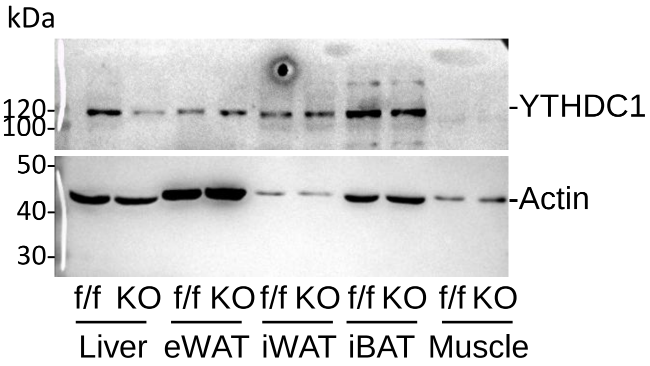

Figure 2b down

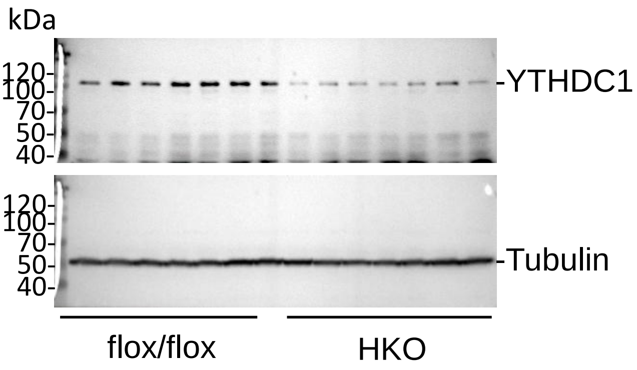

Figure 2o

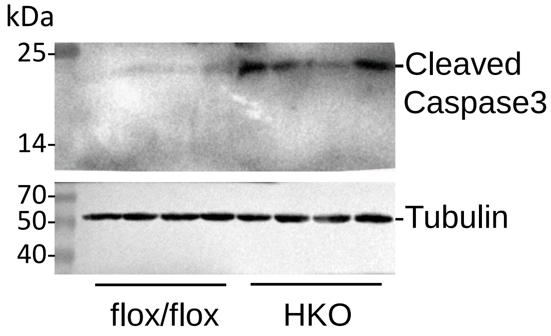

Figure 3a up

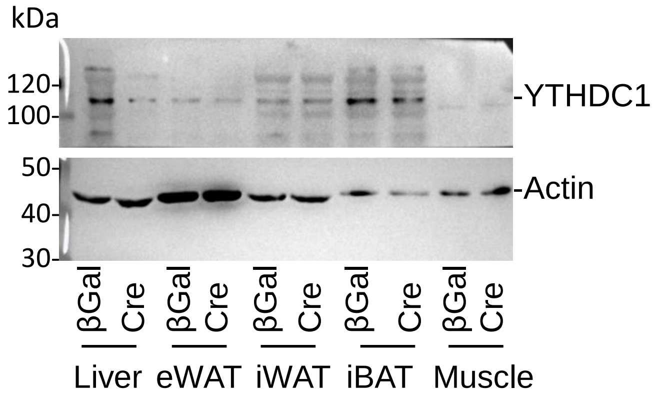

Figure 3a down

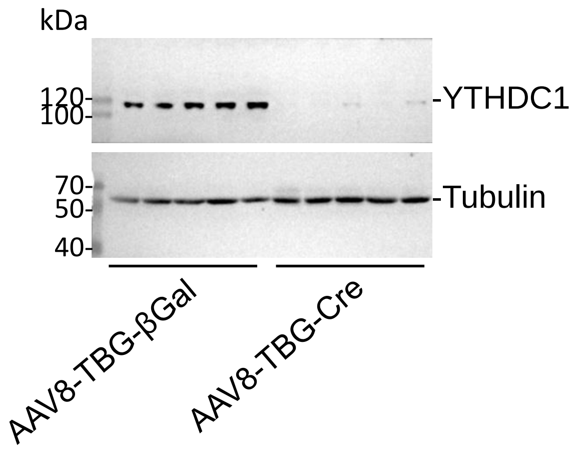

Figure 3p

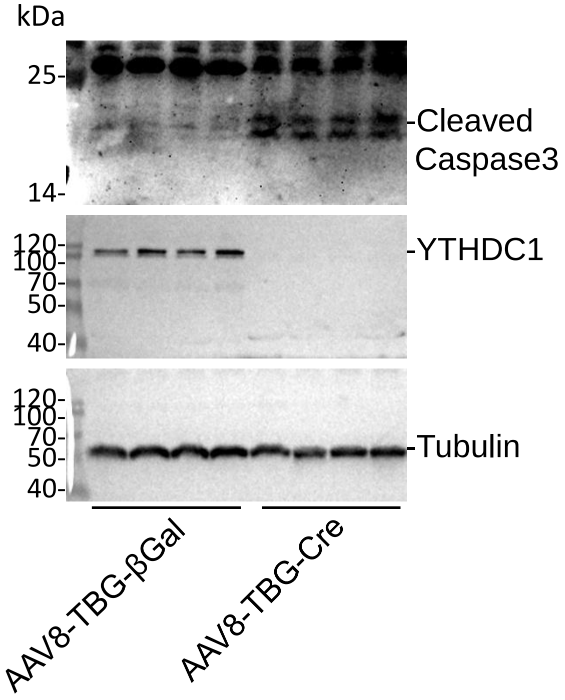

Figure 6f

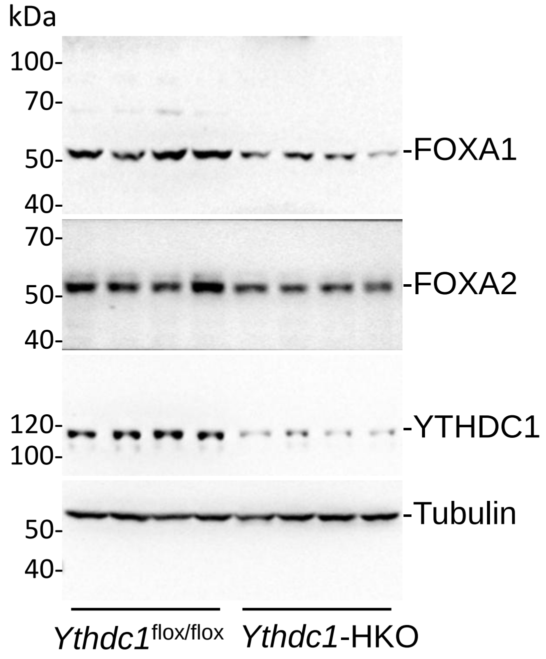

Figure 6i

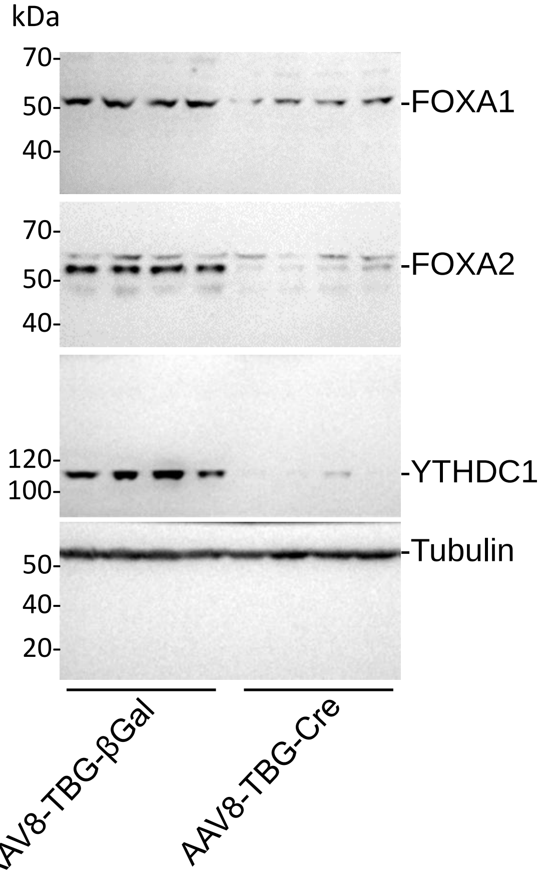

Figure 7b

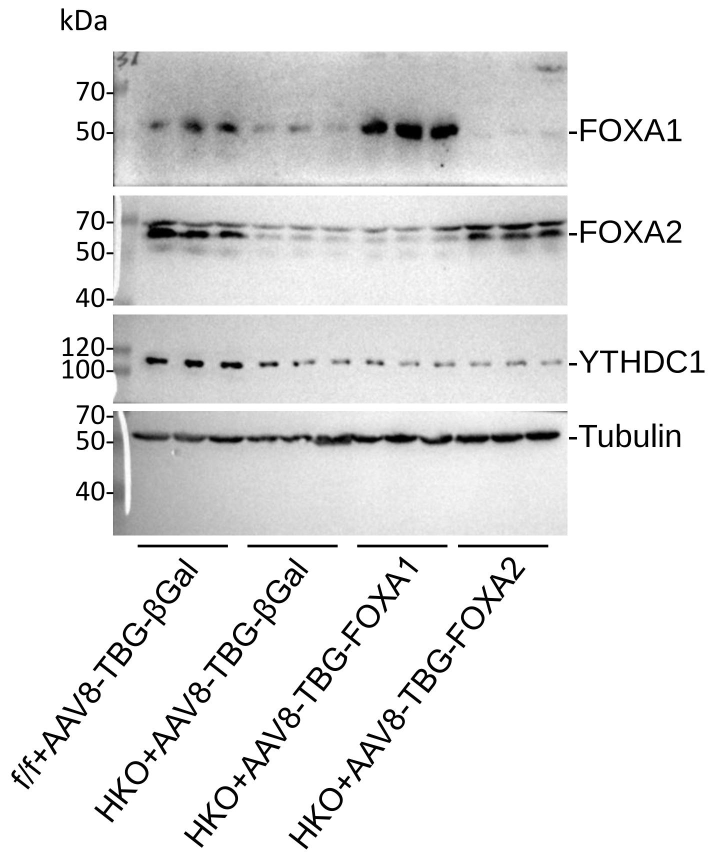
kDa
70-
50-
-FOXA1
70-
50-
40-
-FOXA2
120-
100-
-YTHDC1
70-
50-
40-
-Tubulin
f/f+AAV8-TBG-βGal
HKO+AAV8-TBG-βGal
HKO+AAV8-TBG-FOXA1
HKO+AAV8-TBG-FOXA2

Figure 8b

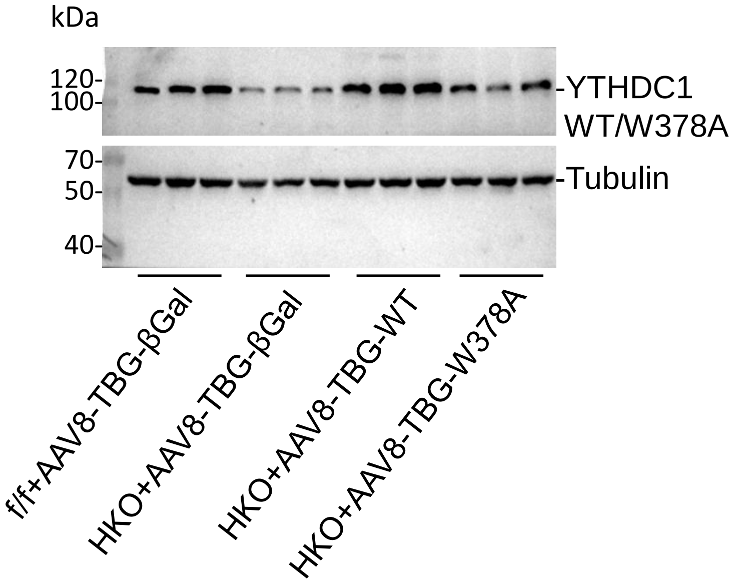

Figure 8m

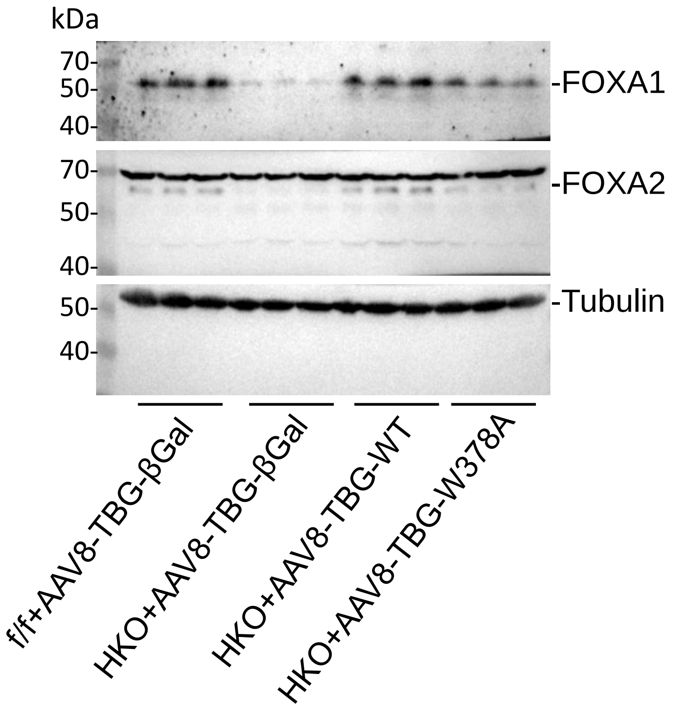

Supplementary Figure 3c

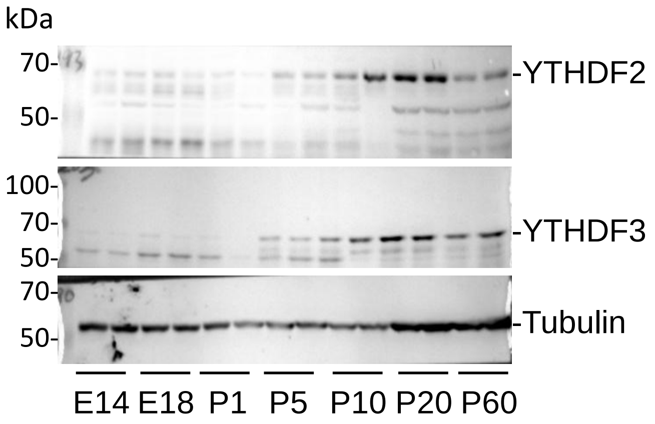

Supplementary Figure 4a

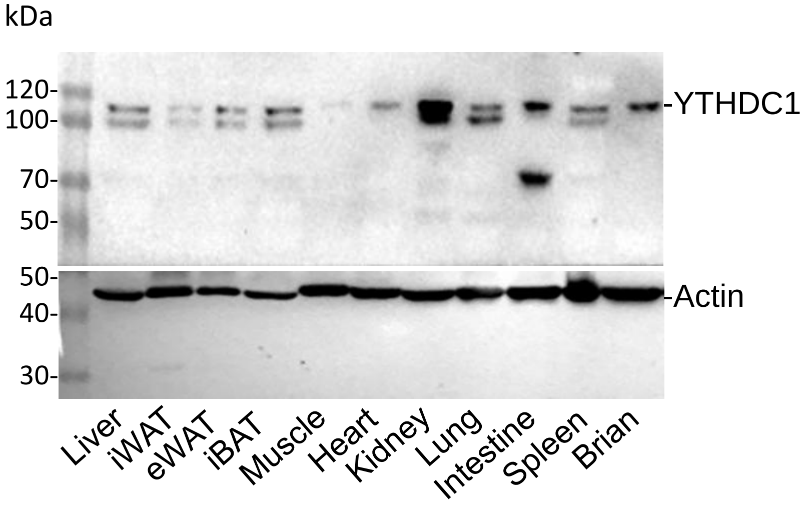

Supplementary Figure 4b

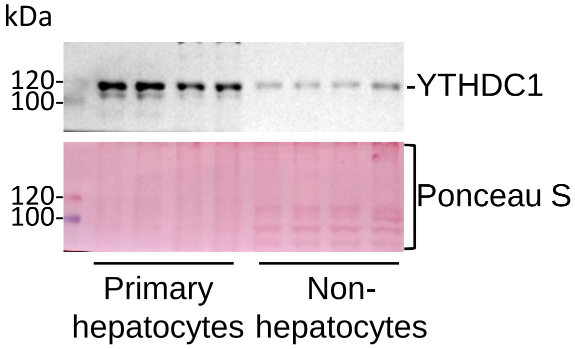

Supplementary Figure 10b

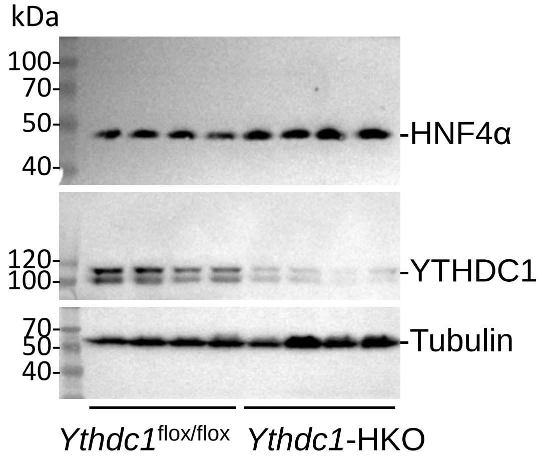

Supplementary Figure 10e

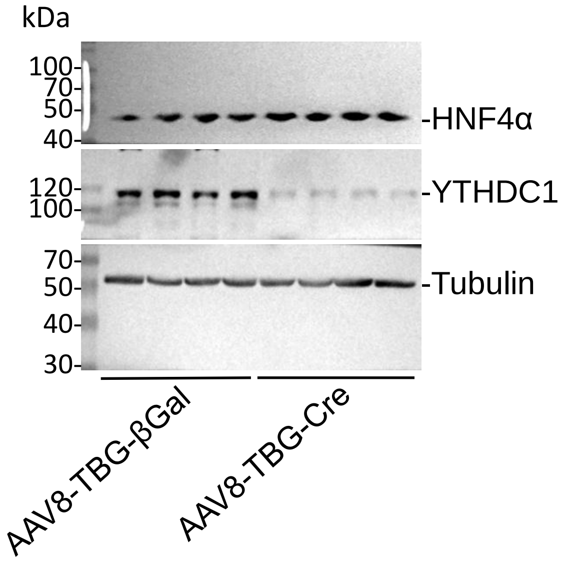

Supplementary Figure 11d

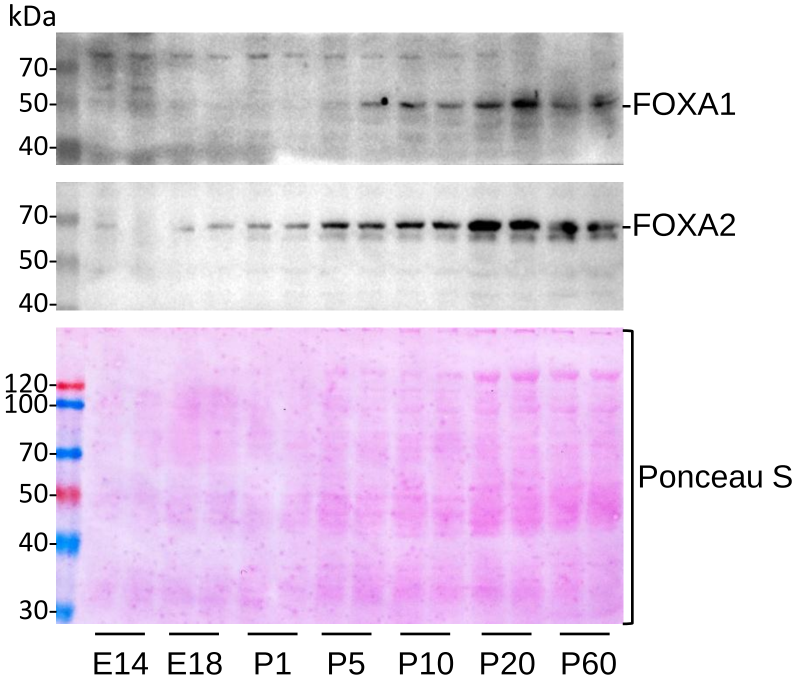

Supplementary Figure 13b

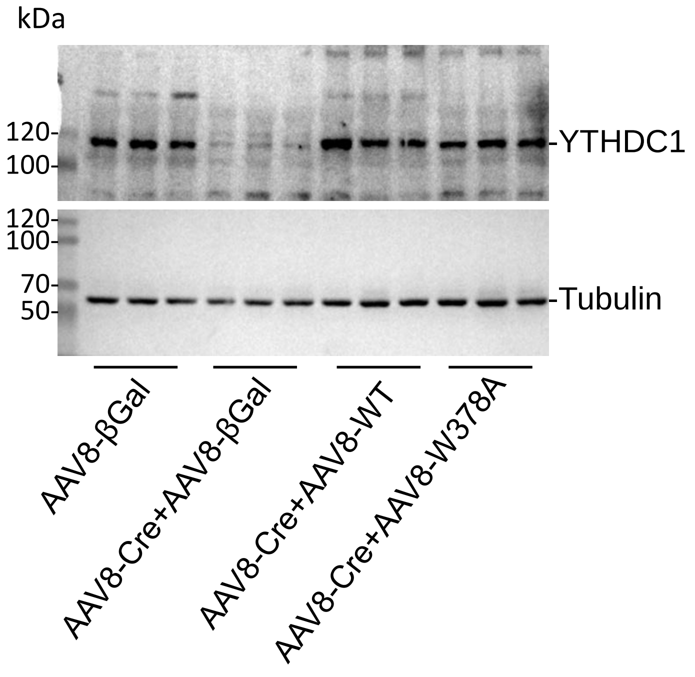

Supplementary Figure 15b

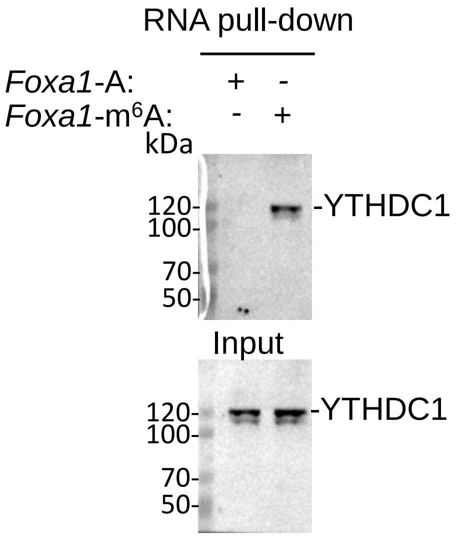

Supplementary Figure 15c

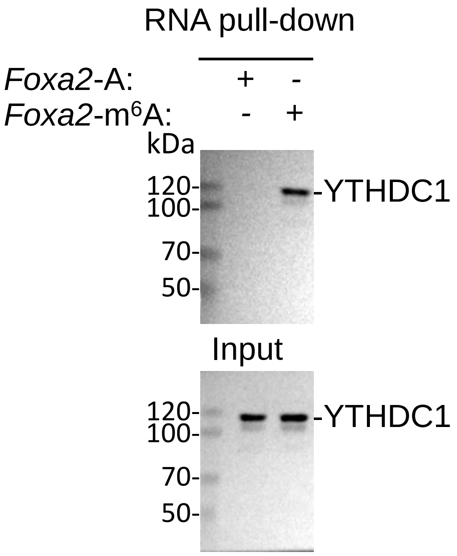

Supplementary Figure 15d

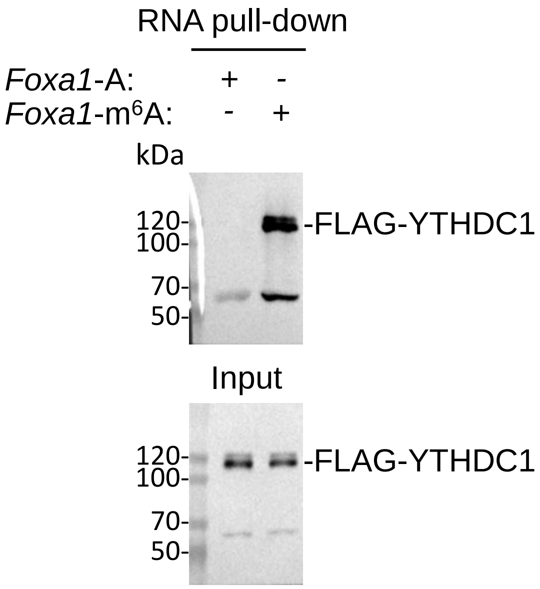

Supplementary Figure 15e

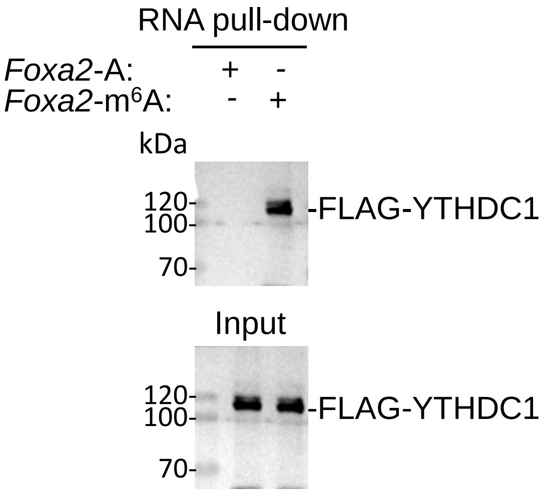

Supplementary Figure 16a

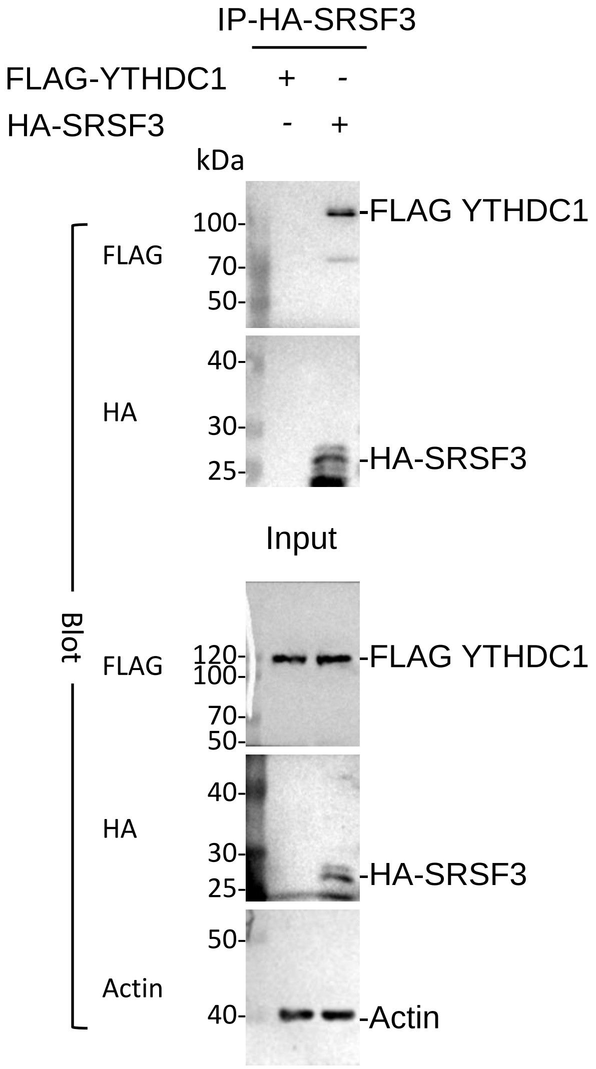

Supplement: Supplementary file 2 — Supporting Information [file ADVS-12-e05725-s001.pdf]
