# Supplementary material for: YTHDC1 Is Essential for Postnatal Liver Development and Homeostasis
Source: Adv Sci (Weinh). 2025 Jun 19;12(35):e05725. doi: 10.1002/advs.202505725 (PMC12462980; doi:10.1002/advs.202505725)
Supplement: Supplementary file 7 — Supplemental Table 5 [file ADVS-12-e05725-s004.docx]

**Table S5 Primers for qPCR**

| **Genes** | **Forward** | **Reverse** |
| --- | --- | --- |
| *36B4* | 5'-AAGCGCGTCCTGGCATTGTCT-3' | 5'-CCGCAGGGGCAGCAGTGGT-3' |
| *Ythdc1* | 5'- CGGGAGGAGAAAGATGGGGA-3' | 5'- TGTCGCTTGGTGTCAGTAGAC-3' |
| *G6pase* | 5'- CCGGTGTTTGAACGTCATCT-3' | 5'- CAATGCCTGACAAGACTCCA-3' |
| *Pepck* | 5'- ATCATCTTTGGTGGCCGTAG-3' | 5'- ATCTTGCCCTTGTGTTCTGC-3' |
| *Ythdf2* | 5'-AGCCAATGAGGAAAGGGCATT-3' | 5'-CTCCCCAAACACAGAGACTCAA-3' |
| *Ythdf3* | 5'-TGTTCTATCTTGATTTGACTTTGCT-3' | 5'-ATAGCTGTTATTCTGATTTGTCTGG-3' |
| *Ccl2* | 5'-ACTGAAGCCAGCTCTCTCTTCCTC-3' | 5'-TTCCTTCTTGGGGTCAGCACAGAC-3' |
| *Tnfα* | 5'-CATCTTCTCAAAATTCGAGTGACAA-3' | 5'-TGGGAGTAGACAAGGTACAACCC-3' |
| *Il1b* | 5'-GCCTTGGGCCTCAAAGGAAAGAATC-3' | 5'-GGAAGACACGGATTCCATGGTGAAG-3' |
| *Il6* | 5'-AGCCAGAGTCCTTCAGA-3' | 5'-GGTCCTTAGCCACTCCT-3' |
| *Ifng* | 5'-GCTACACACTGCATCTTGGC-3' | 5'-CATGTCACCATCCTTTTGCCAG-3' |
| *Collagen1a1* | 5'-TCACCTACAGCACCCTTGTG-3' | 5'-GGTGGAGGGAGTTTACACGA-3' |
| *Ccl5* | 5'-CCACTTCTTCTCTGGGTTGG-3' | 5'-GTGCCCACGTCAAGGAGTAT-3' |
| *Tgfb1* | 5'-TTGCTTCAGCTCCACAGAGA-3' | 5'-TGGTTGTAGAGGGCAAGGAC-3' |
| *Mmp9* | 5'-CGTCGTGATCCCCACTTACT-3' | 5'-AACACACAGGGTTTGCCTTC-3' |
| *αSma* | 5'-GGAGAAGCCCAGCCAGTCGC-3' | 5'-AGCCGGCCTTACAGAGCCCA-3' |
| *iNos* | 5'-CAGGGCCACCTCTACATTTG-3' | 5'-TGCCCCATAGGAAAAGACTG-3' |
| *Cxcl5* | 5'-TGCATTCCGCTTAGCTTTCT-3' | 5'-CAGAAGGAGGTCTGTCTGGA-3' |
| *Ccl22* | 5'-GGTGGCTCTCGTCCTTCTTG-3' | 5'-GTGACGGATGTAGTCCTGGC-3' |
| *Gcgr* | 5'- GATCCGAGTACGCTCGAGGA-3' | 5'-CATCTGGCAGAGGTTGCACA-3' |
| *Foxa1* | 5'-TGTGTATTCCAGACCCGTGC-3' | 5'-ACTGGGGAAAATTGTGCGTG-3' |
| *Foxa2* | 5'-TGCACTCGGCTTCCAGTATG-3' | 5'-TCATGCCATTCATCCCCAGG-3' |
| *C2* | 5'-ACAAACGTTAGCGAGGTGGT-3' | 5'-GCTCCCCCAGATTCTCCTTT-3' |
| *C6* | 5'-CAACTAGGTCGATTTGAAGGGTC-3' | 5'-AGAACAGCTGGACCAGTGAG-3' |
| *C8b* | 5'-GGCTGGCCAGTGGGATAAAT-3' | 5'-GAGGAACTTGCTTTGCTGTGG-3' |
| *Igf1* | 5'-ATCTGCCTCTGTGACTTCTTGA-3' | 5'-TAGCCTGTGGGCTTGTTGAA-3' |
| *Nrp1* | 5'-CACAGTGGCACAGGTGATGA-3' | 5'-ACCGTATGTCGGGAACTCTGA-3' |
| *Cyp4a12a* | 5'-AGTGTCCTCTAATGGCTGCAAG-3' | 5'-GATTTGATCACTTGGTCTGTGTG-3' |
| *C9* | 5'-ACCCGTTTCCAGAGAAGAACA-3' | 5'-TGCTTCTTGAGCGAAACCTTTG-3' |
| *Cfh* | 5'-GGATCCACCACATGTGCCAA-3' | 5'-ATTTCCCTGTTGAGTCTCGGC-3' |
| *Sirt5* | 5'-CCTGTGTCTAGTGGTGGGAAC-3' | 5'-CACAGGGTCCGGGAAAATGA-3' |
| *Rorc* | 5'-TGGAGCAGAGCTTAAACCCC-3' | 5'-GGGATCACTTCAATTTGTGTTCTC-3' |
| *F7* | 5'-GGACCATGTAGGGACCAAGC-3' | 5'-TTTTCAGCACAGCCTGCCAT-3' |
| *Abcg8* | 5'-TGGGACTGTACTTCAGGATGC-3' | 5'-GAGGCGATGTCCACCTGGTA-3' |
| *Albumin* | 5'-TGTCAACCCCAACTCTCGTG-3' | 5'-AACATGCTCACTCACTGGGG-3' |
| *Cyp2a4* | 5'-CTTCCTCAGCGTCCTGGTTT-3' | 5'-ACAATTCGGCGAGATCCCAG-3' |
| *Cyp2e1* | 5'-GGCTACAAGGCTGTCAAGGA-3' | 5'-TCCCCAGTCACGGAGGATAC-3' |
